# Live single-cell laser tag

**DOI:** 10.1038/ncomms11636

**Published:** 2016-05-20

**Authors:** Loïc Binan, Javier Mazzaferri, Karine Choquet, Louis-Etienne Lorenzo, Yu Chang Wang, El Bachir Affar, Yves De Koninck, Jiannis Ragoussis, Claudia L. Kleinman, Santiago Costantino

**Affiliations:** 1 Research Center of the Maisonneuve-Rosemont Hospital, Montreal, Quebec, Canada; 2 Department of Ophthalmology, Université de Montréal, Montreal, Quebec, Canada; 3 Department of Human Genetics, McGill University, Montreal, Quebec, Canada; 4 Lady Davis Institute for Medical Research, Jewish General Hospital, Montreal, Quebec, Canada; 5 Institut Universitaire en Santé Mentale de Québec, Québec, Quebec, Canada; 6 McGill University and Genome Quebec Innovation Centre, Montreal, Quebec, Canada; 7 Department of Medecine, Université de Montréal, Montreal, Quebec, Canada; 8 Department of Psychiatry and Neuroscience, Université Laval, Québec, Quebec, Canada; 9 Center of Innovation in Personalized Medicine, Cancer and Mutagen Unit, King Fahd Center for Medical Research, Department of Biochemistry, King Abdulaziz University, Jeddah, Saudi Arabia

## Abstract

The ability to conduct image-based, non-invasive cell tagging, independent of genetic engineering, is key to cell biology applications. Here we introduce cell labelling via photobleaching (CLaP), a method that enables instant, specific tagging of individual cells based on a wide array of criteria such as shape, behaviour or positional information. CLaP uses laser illumination to crosslink biotin onto the plasma membrane, coupled with streptavidin conjugates to label individual cells for genomic, cell-tracking, flow cytometry or ultra-microscopy applications. We show that the incorporated mark is stable, non-toxic, retained for several days, and transferred by cell division but not to adjacent cells in culture. To demonstrate the potential of CLaP for genomic applications, we combine CLaP with microfluidics-based single-cell capture followed by transcriptome-wide next-generation sequencing. Finally, we show that CLaP can also be exploited for inducing transient cell adhesion to substrates for microengineering cultures with spatially patterned cell types.

## Abstract (web summary)

Cell labelling in a non-invasive and genetic engineering-free manner is crucial to cell biology applications. Here the authors develop cell labelling via photobleaching (CLaP), that uses laser illumination to label individual cells for genomics, cell-tracking, flow cytometry or ultra-microscopy.

## Graphical abstract

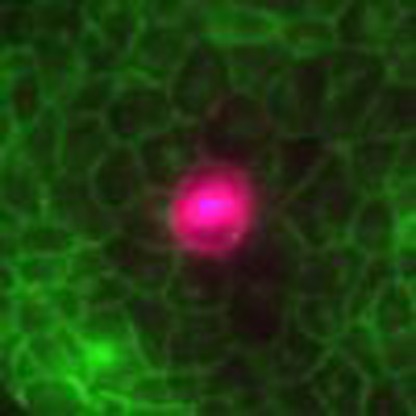

Cellular labels are essential components in the toolbox to build our current understanding of biological function. Yet, a versatile, efficient and non-invasive approach to tag individual cells chosen upon observation is still lacking. The vast majority of methods for generating fluorescently labelled cells rely on biochemical characteristics that are common to an ensemble of cells in a sample, and lack the specificity given by imaging. Widely used methods include transfection of genes encoding fluorescent proteins, membrane-permeable dyes or antibody labelling. These approaches do not allow targeting specific cells among a large population of the same type. Furthermore, their efficiency and specificity are highly dependent on stochastic events and molecular affinity properties, often yielding a sub-optimal fraction of correctly labelled cells. Spatially targeted methods, such as single-cell electroporation^1^,^2^, microinjection^3^, laser capture microdissection^3^,^4^,^5^ or transfection of photo-switchable proteins that change properties upon illumination^6^,^7^,^8^ are often invasive, labour-intensive or lack accuracy, rendering them impractical for a wide range of applications^9^,^10^.

Here we introduce a novel laser-based technique, cell labelling via photobleaching (CLaP), for labelling individual cells in culture. Specific cells can be chosen based on their morphological characteristics, dynamic behaviour, localization in the sample at a given time, or any visible feature that distinguishes the cells of interest from an ensemble. CLaP allows combining the accuracy and versatility of image-based selection with the high throughput of automated cell-sorting methods, thus permitting experiments that account for cellular context or temporal dynamics, such as transcriptomic profiling preserving spatial information. The method does not require previous knowledge of cell surface markers, uses off-the-shelf reagents, and may be implemented on a standard confocal microscope without hardware or software modification.

## Results

### Cell labelling

**Figure 1:**
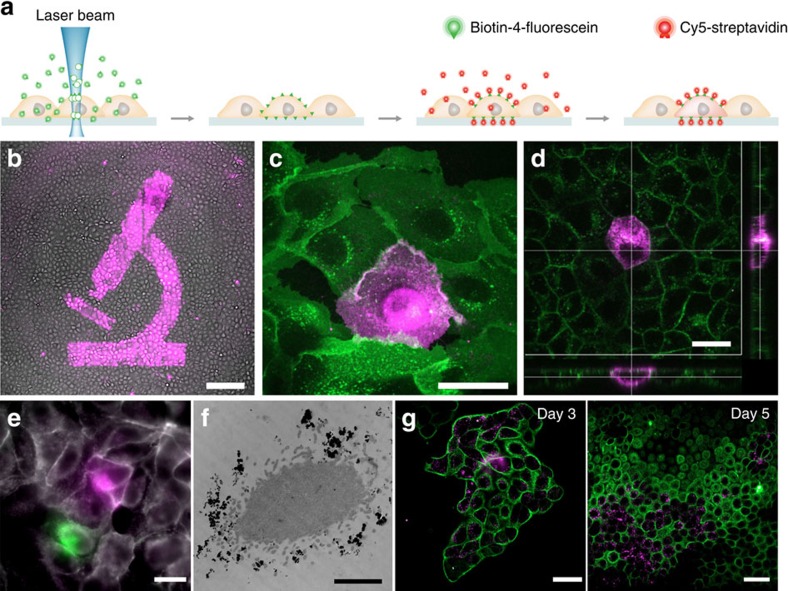
Cell labelling. (**a**) Outline of the method. Cells are incubated with B4F, a small molecule that can easily reach the cell membrane, including the space between the glass surface and the cell. A laser beam photobleaches and crosslinks fluorescein-conjugated biotin. After rinsing, only illuminated cells retain biotin molecules on their plasma membrane and are revealed with fluorescent streptavidin. Biotin molecules attached to the plasma membrane freely diffuse along the lipid bilayer to yield a rather uniform distribution of fluorophores throughout the cell. (**b**) Examples of labelled cells. Low-magnification image of confluent MDCK cells labelled with Alexa-647-Streptavidin (magenta) overlaid on the bright-field image. Scale bar, 200 μm. (**c**) Average confocal projection of a tagged single MDCK cell. The bright circle observed inside the cell boundaries corresponds to streptavidin bound to the glass, marking the region scanned by the laser. Scale bar, 20 μm. Green corresponds to Wheat Germ Agglutinin-Alexa-488, magenta corresponds to Alexa-647-Streptavidin. (**d**) Confocal image and *X-Z* and *Y-Z* projections at day 0 illustrating membrane fluorescence distribution. Scale bar, 20 μm. (**e**) Two-colour CLaP obtained by repeating the procedure sequentially and using ARPE-19 cells stained with Alexa647-Streptavidin in magenta, Alexa555-Streptavidin in green and WGA-Alexa350 in grey. Scale bar, 10 μm. (**f**) Single labelled cell electron microcopy image, where HRP-Streptavidin was revealed with DAB, mostly concentrated in filopodia. Scale bar, 2 μm. Additional images can be found in Supplementary Fig 1. (**g**) Fluorescent puncta become visible one day after CLaP and proliferating cells can be tracked for up to at least 5 days. Scale bar, 20 μm. Magenta: CLaP-labelled cells, Alexa-647-Streptavidin. Green: non-tagged cells, Wheat Germ Agglutinin-Alexa-488. DAB, 3–3´diaminobenzidine tetrahydrochloride .

CLaP is related to laser-assisted protein adsorption by photobleaching^11^,^12^,^13^, a method developed to engineer cell culture substrates by creating protein patterns of optical resolution at a high dynamic range of concentrations. In LAPAP, a laser is used to bind fluorescent biotin conjugates to solid surfaces and hydrogels via free radicals generated by photobleaching. Instead of focusing on inert surfaces, CLaP tethers biotin molecules to the plasma membrane of living cells using a low-intensity laser beam (Fig. 1a). Biotin-4-fluorescein (B4F) is added to the cell culture medium and a laser, tuned near the absorption peak of the dye, is then focused on individual cells of choice, generating reactive oxygen species in close vicinity of the plasma membrane that lead to biotin crosslinking (Supplementary Note 1). Since the entire process occurs in a small region outside the cell, significant phototoxicity is avoided. The irradiated cells are then revealed by incubating the culture with streptavidin conjugates. By choosing among different types of such streptavidin conjugates, cells can be tagged with fluorescence (Fig. 1b–e), electron-dense molecules (Fig. 1f and Supplementary Fig. 1) or other labels. The procedure can be repeated sequentially using different colour streptavidin conjugates to obtain distinct colour tags within the same sample (Fig. 1e). Tethered biotin spreads along the cell surface via lateral diffusion in the plasma membrane, resulting in a relatively uniform cell staining (Fig. 1d).

Biotin tags can be created with high precision at the single-cell level (Fig. 1). The incorporated mark is well suited for monitoring cell location, movement and progeny, since it displays convenient tracking properties: stable, non-toxic, well retained in cells for at least 5 days (Fig. 1g), and transfers by cell division (Fig. 1g) but not to adjacent cells in a population (Fig. 1c–g). Moreover, the label is resistant to routine cell culture procedures. Cells tagged with biotin, resuspended from the substrate with trypsin and reseeded were revealed with fluorescent streptavidin and identified among a large population of unstained cells after 24 h (Supplementary Fig. 2). Fluorescence becomes fainter in time as cells divide, and possibly as proteins are degraded, but subsequent generations of fluorescent daughter cells can be found in the dish (Fig. 1g). Streptavidin tags are mostly restricted to the plasma membrane during the first hours. They are later gradually internalized, forming bright cytoplasmic puncta (Fig. 1g).

The amount of biotin-streptavidin complexes bound to the cell membrane scales with the number of molecules that have been photobleached. Hence, the intensity of the cellular stain can be controlled by modulating the total laser energy. We typically deliver 200 μJ over 2 s within a small central area of the cells (∼100 μm^2^, smaller than the cell itself) to obtain a clear staining. Restricting this area to the centre of the cells decreases the likelihood of covering neighbouring cells, which would result in lower specificity. The average fluorescence intensity of the label is proportional to the laser beam energy, in the range between 150 and 400 μW (Supplementary Fig. 3).

**Figure 2:**
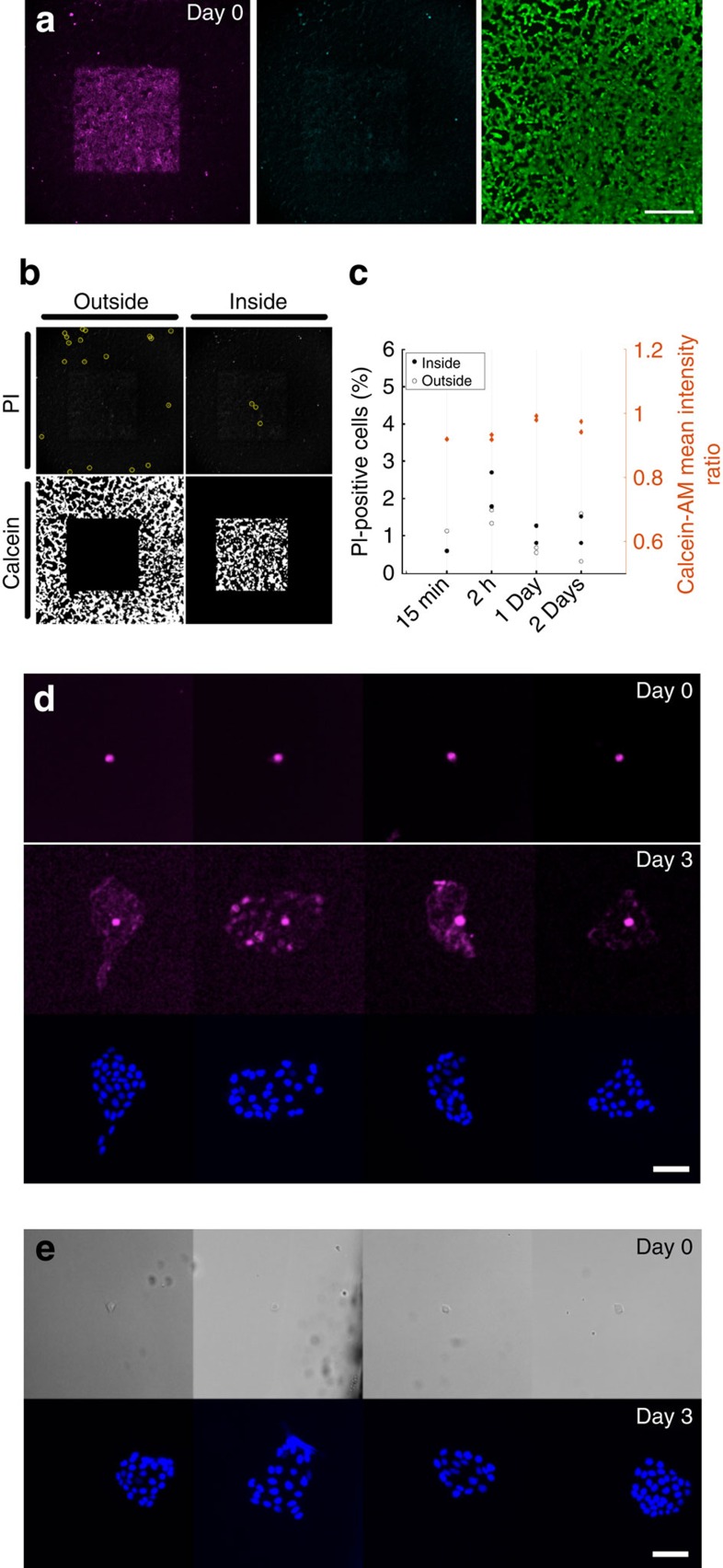
CLaP-labelled cell viability and proliferation. (**a**) Epifluorescence images of CLaP-labelled cells. (left, magenta) Fluorescent streptavidin channel. (middle, blue) Propidium iodide, indicating dead cells. (right, green) Calcein, indicating viable cells. Scale bar, 300 μm. (**b**) Images were segmented to assess the fraction of dead cells inside and outside the illuminated region using the PI images. The mean Calcein-AM intensity was computed within tagged and non-tagged cells on the segmented images and the ratio between these values was used for quantification. (**c**) Cell viability was quantified at four different time points using both stains, inside and outside the illuminated regions. No significant difference was observed within illuminated (inside) and non-illuminated (outside) cells. The complete series of images used for this quantification can be found in Supplementary Fig. 12. (**d**) Isolated MDCK cells were tagged with CLaP using streptavidin-Alexa Fluor 647, and imaged immediately (top). After 3 days in culture, cells were fixed, stained with DAPI, and imaged (bottom). Single cells proliferated to 28 cells in average with s.d.=5.3. Scale bar, 50 μm. (**e**) As a control, non-tagged MDCK cells (top) were kept in culture for 3 days, fixed and stained with DAPI. Cells proliferated to 28.5 cells in average with s.d.=6.2. Scale bar, 50 μm.

The minimal invasiveness of the procedure is based on photobleaching taking place extracellularly, and cell viability and proliferation do not seem to be affected. To assess cell viability, we used CLaP to tag a square region in two confluent cultures of ARPE-19 cells with streptavidin-Alexa Fluor 647. Viability staining with Calcein-AM and propidium iodide (Fig. 2a), followed by image segmentation and quantification, showed no increase in cell death after 2 days, measured at four different time points (Fig. 2b-c). To assess effects on cell proliferation, we tagged isolated cells and quantified the progeny after 3 days (Fig. 2d,e). Once again, no differences were observed between tagged and untagged cells. Finally, the safety of CLaP was confirmed by gene expression profiling of individually isolated cells, where no significant differences in gene expression were observed upon tagging (see below).

### Single-cell isolation and genomics

**Figure 3:**
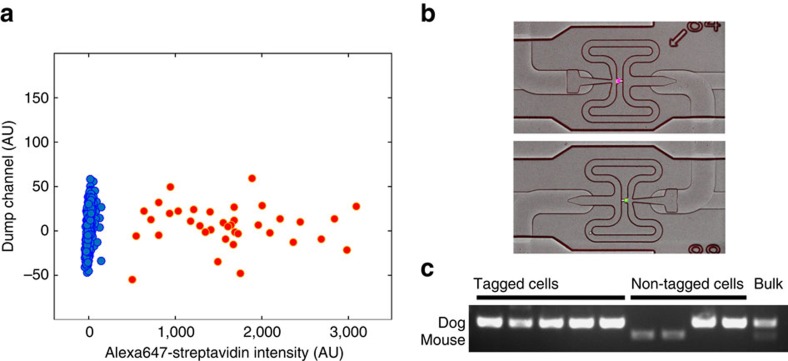
Sorting of single CLaP-labelled cells. (**a**) FACS identification of CLaP-labelled cells. After gating on live singlet cells to distinguish laser-tagged cells, the scatter plot shows two populations of cells separated in the fluorescent streptavidin channel, with colour automatically assigned using a hierarchical clustering algorithm (code available as Supplementary Software). Autofluorescence was used to spread the cells in the vertical axis in order to visualize individual cells. (**b**) MDCK (dog) cells were tagged by CLaP in a co-culture experiment, where ∼5% of all cells were targeted. A mix of cells were then individually captured on a Fluidigm C1 chip and visualized. Positive Alexa-647 CLaP tagged cell (magenta) and negative non-tagged cell (green) are shown. (**c**) PCR amplification confirms species of origin of each tagged cell, isolated with the C1 platform. Non-tagged cells, also isolated with C1, consist, as expected, of a mixture of dog and mouse cells. Rightmost lane corresponds to DNA from a bulk extraction on the rest of the sample. The complete gel and molecular markers are shown in Supplementary Fig. 7.

To be studied, tagged cells need to be accurately identified and captured. We first tested CLaP for widely-available fluorescence-activated flow cytometry (FACS), a common technology used to sort cells. Three standard gates were defined to count exclusively events originated from isolated viable cells (see Supplementary Fig. 4 for details). CLaP-tagged cells were detected using the Alexa Fluor 647 signal solely from events going through the three previous gates versus a dump channel (Fig. 3a).

Next, to demonstrate the potential of the method for single-cell genomic applications, we combined CLaP with microfluidics-based single-cell capture followed by PCR assays and transcriptome-wide next-generation sequencing. We first evaluated the specificity of CLaP and subsequent capture by a co-culture experiment where we tagged only one cell type, captured individual cells, visualized them and analysed their DNA by PCR. For this, we co-cultured mouse 3T3 fibroblasts expressing mNeonGreen and dog MDCK cells (Supplementary Fig. 5). We exclusively tagged MDCK cells with a far-red fluorescent streptavidin label, targeting ∼5% of the total number of cells in the dish. Viability test using calcein-AM/ethydium homodimer before loading the microfluidic device confirmed the non-toxicity of CLaP (Supplementary Fig. 6). We isolated 96 cells using the Fluidigm C1 microfluidic system, among which five were CLaP tagged (Fig. 3a). On-chip cell lysis, followed by DNA amplification, allowed us to confirm by PCR the correct species of origin for all tagged cells (Fig. 3b and Supplementary Fig. 7).

**Figure 4:**
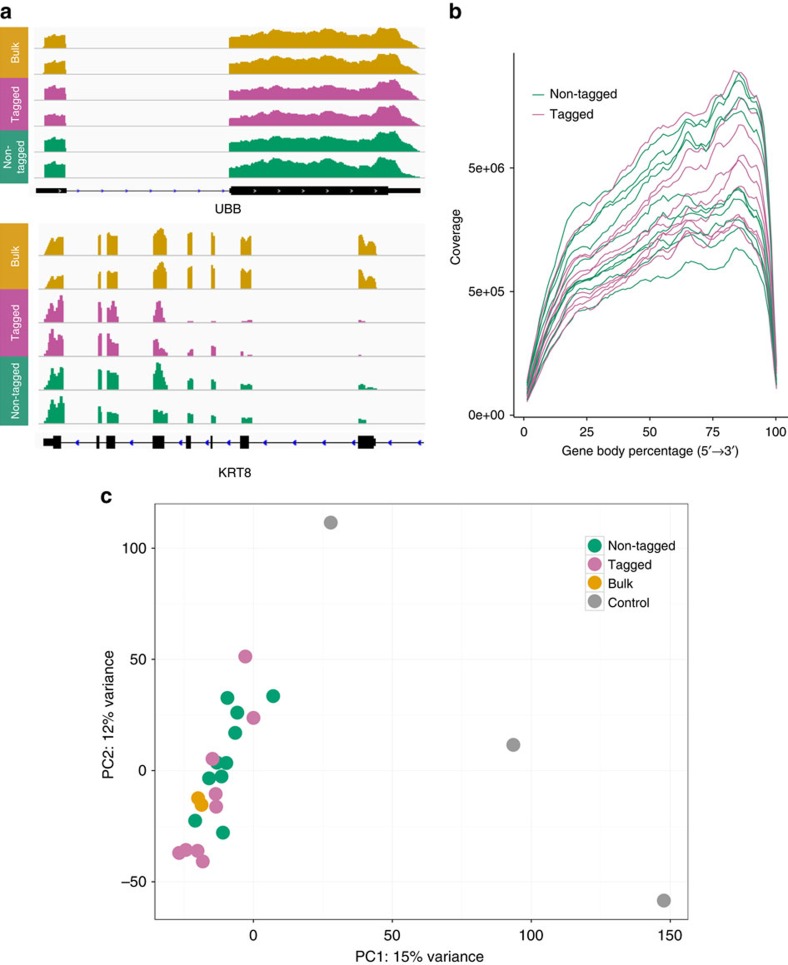
Single-cell CLaP-labelled RNA transcriptome analysis. (**a**) Example of RNA-Seq data obtained for one highly expressed gene (UBB) and one RPE marker (KRT8) from bulk (yellow), tagged single cells (orange) and non-tagged single cells (green). All cells are shown in Supplementary Fig. 8. (**b**) Coverage uniformity over gene body. Using RSeqC, all transcripts were scaled to 100 nt and the number of reads covering each nucleotide position was computed. The slight 3′ bias, reported for SMARTer Ultra Low RNA kit, is expected. Magenta: CLaP tagged cells. Green: non-tagged cells. (**c**) Global effects of CLaP on cells were evaluated by unsupervised clustering of samples based on expression profiles, which consistently groups together tagged and untagged cells. Negative controls were derived from empty wells (no cells captured), to account for potential contamination, cell debris and other factors and an empty well containing RNA spike-in mix only. Different conditions for the clustering, including subsampling genes, bootstrapping or excluding control samples were assessed, with consistent results (Supplementary Figs 10 and 11).

We then demonstrated that single-CLaP-positive cells can be isolated from a large population for single-cell transcriptomic analyses, using a human retinal pigment epithelial cell line. After single-cell capture, RNA-Seq was obtained from CLaP-tagged (*n*=9) and untagged (*n*=10) individual cells, in addition to bulk samples consisting of (i) 200 cells pooled and (ii) cDNA from 5 ng total RNA extracted from the bulk culture. We computed a number of quality control metrics to verify that CLaP does not interfere with protocols of sample preparation for transcriptomic experiments. Comparisons of tagged versus untagged cells revealed no significant differences regarding total coverage, GC content and coverage distribution over the genome structure (Supplementary Data and Supplementary Figs 8 and 9). More importantly, gene expression profiling indicates no major changes associated with the procedure, as unsupervised clustering of samples based on expression profiles (principal component analysis (PCA) and hierarchical clustering with bootstrapping) consistently groups tagged and untagged cells together (Fig. 4 and Supplementary Figs 10 and 11). More subtle effects on gene expression levels were assessed through a differential expression analysis, which showed statistically significant differences in only 24 genes, representing 0.34% of all detected genes (Supplementary Data). Altogether, these results are consistent with the viability and proliferation assays, indicating that CLaP does not affect regular cell function.

### Laser-controlled spatial distribution of cells

**Figure 5:**
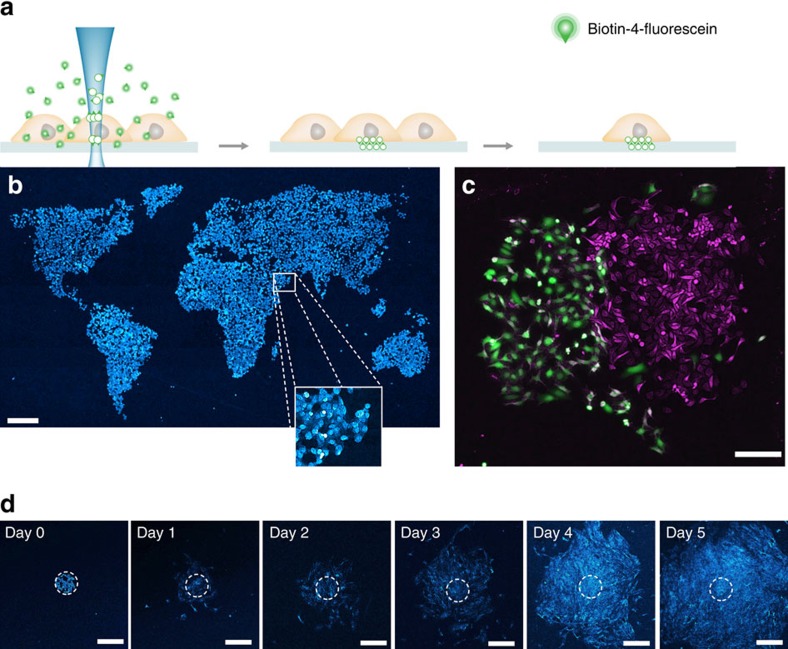
Induced transient cell adhesion. (**a**) B4F is a small molecule that can easily reach the space between the glass surface and the cell membrane. Reactive species induced by photobleaching B4F create transient adhesions between the cell basal membranes and the substrate. (**b**) Bright-field, contrast-enhanced image of a miniature world map created using ARPE-19 cells. Scale bar, 400 μm. (**c**). Spatially segregated U2OS cells expressing GFP (green) and bright-field contrast-enhanced ARPE-19 cells (magenta). Scale bar, 200 μm. (**d**) Cell proliferation after being transiently adhered to the substrate is demonstrated by daily bright-field illumination images. Image contrast was enhanced using the method described in the methods. Scale bar, 250 μm.

The same photochemistry used to tether biotin molecules to the plasma membrane induces transient adhesions of cells to the substrate, which are resistant to EDTA and trypsin treatment. As previously shown^14^, small fluorescent molecules can diffuse between the glass substrate and the cell membrane, allowing crosslinking of the cells to the cover slip upon photobleaching^14^. These adhesions can thus be exploited to tailor the spatial distribution of cells by automating both laser illumination and sample movement (Fig. 5a,b), and subsequently detaching non-tagged cells using proteases or chelators. Cell adhesions induced by CLaP are transient, and cells keep proliferating and migrating away from the initial region where they were attached, spreading to cover the full field of view of a × 10 objective at day 5 (Fig. 5d). The transient nature of cell adhesions and the limited impact of the procedure on cell proliferation are probably due to the choice of CLaP fluorophores and the use of low-intensity visible light. The method, thus, constitutes a practical way to select clones of proliferating cells based on visual characteristics, which can be both morphologic and behavioural.

We also tested the possibility of combining different cell types in spatially segregated regions of the substrate, for potential use of the technique in tissue engineering. We first illuminated confluent U2OS cells in a square region, detached non-irradiated cells, seeded ARPE-19 cells in the dish and allowed proliferation. After 24 h, we re-illuminated both cell types to create adjacent squares, and detached the rest (Fig. 5c). Of note, to create cell adhesions that withstand treatment with chelators and proteases, the entire cell surface needs to be illuminated, as opposed to the small fraction required to adsorb tags that diffuse along the membrane.

## Discussion

In this work, we introduced a method to tag individual cells with a laser, and demonstrated the potential for fluorescence and electron microscopy, as well as FACS and single-cell next-generation sequencing. The most important characteristic of CLaP is the image-based criteria for cell selection, which presents two critical advantages: versatility and potential for automation. This opens the door for experiments that interrogate the transcriptional profiles of cells depending on their microenvironment and spatiotemporal dynamics, permitting to tag, for instance, only fast, large, round, granular, isolated or distant cells. It also enables to study cell-to-cell communication, in contexts such as immune cell activation or synaptic interactions, where cellular cross-talk induces context-specific molecular changes for which no marker is available. The method allows isolation of cells that have undergone a transient event detectable in a microscopy field. This novel capability is particularly relevant in areas where tissue heterogeneity plays a major role, including development, cancer biology, immune response, stem cells or neurobiology^9^,^15^.

Recently, a few approaches have been developed for transcriptome sequencing preserving spatial information. *In situ* methods include performing mRNA capture (TIVA^16^) or sequencing reactions (FISSEQ^17^) directly inside intact cells. While CLaP does not have the potential of providing subcellular information or *in situ* expression profiling, it presents several advantages that make it a convenient choice for a number of applications. First, the protocol is short and simple, without requiring special probes, and a standard confocal microscope can be used for tagging, making it a very accessible method. Of course, a dedicated system with more powerful lasers is useful to accelerate the procedure, and programmable motorized stages allow creation of arbitrary illumination patterns. Second, the method is not tied to any particular library preparation protocol or sequencing technology, an important feature in a field that is evolving at a very fast pace. While emerging *in situ* approaches have great potential, practical testing and information on biases and reproducibility are still lacking, and limitations on sequencing depth have to be overcome. CLaP-labelled cells, on the other hand, can be analysed using standard, widely tested capture and sequencing protocols.

Several extensions to the method can be envisaged. Tags are not restricted to fluorescent modalities; here we also used electron-dense labels, and bound molecules can easily be extended to magnetic particles, antibodies, nucleotide sequences, drugs or functional macromolecules. Further extensions include colour barcoding, to allow separation of more than one group of cells. We have shown that at least two distinct fluorescent tags can be added to a sample (Fig. 1e). The maximum number of simultaneous labels is, however, limited by emission cross-talk between image channels, non-specific background of B4F, which adds noise to green Streptavidin conjugates, and the diversity of commercially available fluorophores.

We have here demonstrated the use of CLaP on monolayers of cultured cells. The cellular specificity of CLaP is particularly high using cell lines, but it may be slightly decreased in primary cultures, which often include dead cells with permeable membranes and dissection debris that can yield non-specific staining due to adsorbed B4F and streptavidin. Finally, in tissue CLaP would open a number of exciting possibilities, even if diffusion of reagents through the extracellular matrix for both tagging and rinsing may slow down the procedure. Changing continuous laser illumination for ultrashort laser pulses as a way to spatially confine photobleaching by two-photon absorption in three-dimensional environments will potentially extend this approach to *ex vivo* and *in vivo* applications.

## Methods

### Cell culture

ARPE-19 cells were cultured in F12 (Thermofisher Scientific, 11765-062) medium supplemented with Gibco Glutamax supplement, 10% FBS and antibiotics. MDCK cells were cultured in DMEM (Thermofisher Scientific, 12561-056) with 10% FBS, Gibco Glutamax supplement and 1% antibiotics and 3T3 and IMCDs in 45% DMEM, 45% F12 with 10% FBS, Gibco Glutamax supplement and 1% antibiotics. Cells were cultured in flasks for amplification and then plated in glass bottom dishes (Mattek p35GC-1.0-14-C) for optimal optical quality.

### Single-cell labelling

Cell illumination was performed coupling a 473 nm laser (Laserglow technologies, LRS-0473-GFM-00050-05) to an adapted confocal microscopy module (Thorlabs) mounted on an inverted microscope (Olympus, IX71). In order to determine the localization of the focal spot of the laser, B4F (Sigma Aldrich, B9431-5MG) was dissolved at a concentration of 0.04 mg.ml^−1^ in medium and a drop was dried on a cover slip. Photobleaching of this dried B4F upon illumination with the laser allowed precise localization of the focal spot in the microscope field of view.

Dishes containing cells were placed on the microscope and the cells of interest were chosen and illuminated with a power between 100 and 350 μW during 2 s using a 0.7 NA objective (Zeiss LD Plan-NEOFLUAR, 441370-9970). This objective ensures enough lateral resolution for aiming the focused laser spot to a single cell. To maximize cell selectivity the illuminated region was restricted to a small central region of the cell membrane, keeping the beam as far as possible from neighbouring cells, in order to prevent unspecific tagging. Similarly, the focal plane was set near the top membrane of the cell, which ensures optimal crosslinking efficiency, although this is not a critical issue. After crosslinking, biotin molecules diffuse laterally on the membrane and spread out to the rest of the cell surface. After 3 washes with warm PBS, cells were incubated in streptavidin-compounds at a concentration of 0.05 mg.ml^−1^ in medium for 15 min. The dish was then rinsed three times in PBS before imaging. Overall, the procedure takes 30 min of preparation, 30 min of incubations and washings, and approximately one minute for each stained cell to be found, properly placed in the focal region of the laser and illuminated.

Depending on the experiment, we have used Alexa-647-Strepatavidin (ThermoFisher Scientific, S-21374); Alexa-555-Strepatavidin (ThermoFisher Scientific, S-32355) or Alexa-488-Strepatavidin (ThermoFisher Scientific, S-11223) all at 0.05 mg.ml^−1^ in medium. Neighbouring cells are revealed by incubating the dish during 15 min in alexa-350-wheat germ agglutinin (alexa-350-WGA, ThermoFisher Scientific, W11263) 10 μg.ml^−1^ medium. A detailed online protocol is available at http://www.nature.com/protocolexchange/protocols/4707.

### Cell viability

Cells were irradiated by moving the sample at 170 μm s^−1^ with a laser power of 240 μW at the sample, using a 0.4 NA objective. Samples were incubated with PI and calcein at four different time points, either immediately after the 15 min incubation in streptavidin Alexa 647, or 2 h, 1 day and 2 days after illumination (Fig. 3a).

Quantification was programmed in Matlab by segmenting regions inside and outside the CLaP pattern (Supplementary Software). First, a binary image was obtained from the Alexa Fluor 647 image by applying the Otsu algorithm^18^, from which only the largest foreground object was kept. The bounding box around it was used as mask to distinguish tagged and non-tagged cells.

To segment the images of PI labelled cells, we enhanced objects between 5 and 15 μm wide using a spatial band-pass filter. Local maxima separated at least 15 μm from neighbours were detected, but only those with peak intensities larger than the median of all detected peaks by six times the standard deviation were kept (see Fig. 3b). Finally, such peaks were counted inside and outside the CLaP region.

Otsu algorithm^18^ was used once more to discriminate between positive and negative calcein pixels (see Fig. 3b).

### Cell proliferation

MDCK cells were plated at very low density to obtain single cells separated ∼1mm from each other. After 2 h in culture, they were tagged with CLaP, incubated with fluorescent streptavidin and imaged immediately afterwards. Cells were kept in culture for 3 days, fixed with paraformaldehyde 4% in PBS during 15 min, and stained with DAPI. As a control, a second non-labelled dish was kept in culture and cells were monitored daily to check that no nearby cell cluster merged. To match the appropriate cluster of cells after 3 days, the original *X* and *Y* absolute coordinates of the stained cells at day 0 were saved for subsequent imaging of the exact same region.

### Transient cell adhesions

Cells were cultured in a 35 mm polystyrene dish (Falcon, 35 3001) and incubated with 0.04 mg ml^−1^ B4F (sigma Aldrich, B9431-5MG) dissolved in medium. The laser was moved at 0.17 mm s^−1^ with 240 μW at the sample, and a 0.4 NA objective. Cells were washed quickly with pre-warmed PBS (three times) and then incubated in 0.05 mg ml^−1^ streptavidin-cy5 (Jackson 016-170-084) in medium to simplify the search for the pattern in the dish. After three additional washes, cells were placed back in the medium for 4 h before detachment with EDTA 10 mM (sigma Aldrich E9884-100G).

To obtain spatially segregated cell cultures, a square pattern of U2OS cells was first produced on a 35 mm polystyrene dish (Falcon 35 3001), as described above. ARPE-19 cells were seeded on the same dish at high concentration to reach confluence overnight. Next day, a larger square pattern, including the U2OS cells from the first day, was irradiated to produce a culture of spatially segregated U2OS and ARPE-19 cells.

### Cell proliferation after CLaP-induced adherence

Confluent ARPE-19 cells were irradiated within a 200 μm diameter circular region (Fig. 5, dashed white line) as described above. We incubated cells in EDTA 10 mM for 5 min, for detachment outside the irradiated region. Attached cells were kept in culture (37 °C, 5% CO_2_) during 5 days after CLaP-induced adhesion and the sample was imaged daily using a 10XNA0.4 objective with bright-field illumination.

### Laser and movement automation

The automation approach used to create spatial irradiation patterns was described in detail elsewhere^13^. A set of instructions describing the stage motor movements and laser power is generated using Matlab (The MathWorks, Inc.) scripts. These instructions are executed with a program coded in LabVIEW (National Instruments Corporation). Source code is available as supplementary software.

### Flow cytometry

Approximately 10% of cells in a low concentration culture were individually tagged using Alexa-647-Streptavidin. Cells were suspended using Trypsin 0.25% (ThermoFisher, 25200-072), spun and resuspended in PBS containing 5 μM Sytox Blue Dead Cell Stain (ThermoFisher, S34857). Flow cytometry was performed with a BD LSR Fortessa cell analyser. We used a SSC-W over FSC-A graph to discriminate doublets, and the Sytox Blue channel to gate on live cells. Propidium iodide fluorescence was used as a dump channel. Colours were automatically assigned using a hierarchical clustering algorithm (Supplementary Data).

### Electron Microscopy

Cells were cultured on aclar slices to help the preparation of ultrathin slices. Aclar slices were prepared according to previously published protocol^19^. Briefly, 13-mm-diameter circles were cut inside an Aclar 22c sheet (Honeywell p5000HS). After several washes and sonication in distilled water, slices were washed in ethanol, then with 10% nitric acid. Aclar slices were then coated with poly-D-lysine (100 μg ml^−1^, Becton Dickinson, 354210) for 3 h. Cells were placed in culture on these slices, and staining was conducted in the same condition as previously detailed. Samples were then washed in Sorensen's phosphate buffer and fixed 15 min in 4% paraformaldehyde. After three additional washes in Sorensen's buffer, samples were blocked with 1 ml blocking solution (TBS-5% NGS-0.5%) during 45 min. Streptavidin sites were revealed by reacting the cells for 15 min at room temperature in 0.3% 3–3´diaminobenzidine tetrahydrochloride with ammonium chloride and nickel ammonium-sulfate, and then in the presence of 0.01% H_2_O_2_. Alternatively, cell were incubated with Streptavidin EM-grade 6 nm gold particles, (Electron Microscopy Sciences, 25263) 1:100 in a lysine-glycine blocking solution overnight at 4 °C. Silver intensification of gold particles was performed for 15 min using a Silver enhancement kit (GE Healthcare, RPN491).

Cells were washed in ECS (3 × 5 min), rinsed for 5 min in PB and incubated in 1% osmium tetroxide (OsO_4_) in PB. Finally, the cells were dehydrated through ascending ethanol concentrations and propylene oxide. Sections were then flat embedded in Durcupan ACM. The regions of interest were selected using light microscopy and reembedded in Durcupan ACM blocks. Ultrathin sections were cut with an ultramicrotome (Leica EM UC7) in 50 nm sections, and collected on Formvar-coated one-slot copper grids and mesh grids. The sections were counterstained with lead citrate before observation on a Tecnai 12 transmission electron microscope (100 kV; Philips/FEI) equipped with an AMT V700 camera.

### Single-cell whole-genome amplification

Single-cell whole-genome amplified (WGA) DNA was obtained according to Fluidigm protocol ‘using C1 to generate libraries for DNA sequencing' (PN 100-7135 H1). Briefly, viable single cells were captured on a 17–25 μm ‘C1 Single-Cell Auto Prep IFC for DNA Seq' (Fluidigm 100-5764) and visually confirmed using EVOS FL Auto microscope (Life Technologies). The following mixes were made by combining reagents from the Illustra GenomiPhiT V2 DNA Amplification Kit (GE Healthcare Life Sciences, 25-6600-30) and the C1 Single-Cell Auto Prep Reagent Kit for DNA Seq (100-7357); DTT Mix (193.10 μl PCR Water, 2.30 μl GE Kit Sample Buffer, 2.30 μl GE Kit Reaction Buffer, 2.30 μl DTT (1 M, Fluidigm)), Lysis Mix (19.8 μl C1 DNA Seq Lysis Buffer (Fluidigm), 2.2 μl DTT (1M, Fluidigm)), Reaction-Enzyme Mix (45 μl C1 DNA Seq Reaction Mix, 4.5 μl GE Kit Enzyme Mix, 31.5 μl DTT Mix). Different mixes were then loaded onto the appropriate IFC inlets.

Inside the IFC, single cells were lysed with 9 nl of lysis mix. Total lysis reaction volume was 13.5 nl. Next 18 nl of C1 DNA Seq Stop Buffer (Fluidigm) were added to the lysis mix. Total stop lysis reaction volume was 31.5nL. Next WGA using the multiple displacement amplification method were done at 38 °C for 2hrs using 270 nl of Reaction-Enzyme Mix. Total multiple displacement amplification reaction volume was 301.5 nl. Each single-cell reaction was collected into C1 DNA Dilution Reagent (Fluidigm) on a 96-well plate. The final volume per cell was 23 μl. In parallel, Tube controls (TC) DNA from ∼200 cells (TC_200), a positive control (GE supplied control DNA) and a negative control were similarly processed on a BioRad T100 Thermocycler. Briefly, each TC was lysed with 2 μl of lysis mix. Total lysis reaction volume was 3 μl. Next the 3 μl lysis reaction is neutralized using 4 μl of C1 DNA Seq Stop Buffer (Fluidigm). Total stop lysis reaction volume was 7 μl. Then 1.05 μl of the stop lysis reaction was amplified using 8.95 μl of Reaction-Enzyme Mix. Total WGA reaction volume was 10 μl. WGA material was quantified with Qubit dsDNA HS Assay Kit (ThermoFisher, Q32851). The average DNA yield after WGA of single cells, TC200, positive control and negative control was 117, 437, 348 and 3.35 ng, respectively.

### Identification of cells species by PCR

Species confirmation was carried out by PCR using Taq (ThermoFisher 18038-042), and 1 μl of the DNA solution prepared with the C1 sorter (Fluidigm) as template, in 25 μl of total reaction volume. The following two primer pairs were used: *Cytb1L*(5′- CATAGCCACAGCATTCATGG -3′), *Cytb1R*(5′- GGATCCGGTTTCGTGTAGAA -3′), and *Cytb2L*(5′- CCTCAAAGCAACGAAGCCTA -3′), *Cytb2R*(5′- TCTTCGATAATTCCTGAGATTGG -3′), which amplify fragments of 247 nt and 196 nt from the mitochondrial gene *Cytb* of dog and mouse, respectively. The complete gel and molecular markers are shown in Supplementary Fig. 7.

### RNA sequencing

ARPE-19 cells in culture were isolated as described above. Single-cell mRNA-Seq was done according to the Fluidigm protocol ‘Using C1 to Generate Single-Cell cDNA Libraries for mRNA Sequencing' (PN 101-7168 H1). Briefly, viable single cells were captured on a 17–25 μm ‘C1 Single-Cell Auto Prep Integrated Fluid Circuit (IFC)' for mRNA-Seq (Fluidigm 100–5761) and visually confirmed using EVOS FL Auto microscope (Life Technologies). To control for variability in mRNA-seq experiments, 92 ERCC spike-ins (Ambion 4456740) were added to the lysis mix (Fluidigm). The following mixes were made by combining reagents from the SMARTer Ultra Low RNA Kit (Clontech, 634833), C1 Reagent Kit for mRNA Seq (100-6201) and 92 ERCC spike-ins (Ambion 4456740). RNA Spikes mix (0.5 μl of 92 ERCC spike-ins (Ambion 4456740), 24.5 μl of Loading Reagent (Fluidigm)), Lysis Mix (1 μl RNA Spikes mix, 0.5 μl RNase Inhibitor (Clontech), 7.0 μl of 3′ SMART CDS Primer IIA (Clontech), 11.5 μl Clontech Dilution Buffer), Reverse Transcription (RT) Mix (1.2 μl Loading Reagent (Fluidigm), 11.2 μl 5X First-Strand Buffer (Clontech), 1.4 μl Dithiothreitol (Clontech), 5.6 μL dNTP Mix each dNTP at 10 mM (Clontech), 5.6 μl SMARTer IIA Oligonucleotide (Clontech), 1.4 μl RNase Inhibitor (Clontech), 5.6 μl SMARTScribe Reverse Transcriptase (Clontech)). These mixes were then loaded onto the appropriate IFC inlets. Inside the IFC, each single cell was lysed with 9 nl of lysis mix. Total lysis reaction volume was 13.5 nl. Next, mRNA was reverse transcribed at 42 °C for 1.5 h using 18 nl of RT mix. Total RT reaction volume was 31.5 nl. The full-length cDNA was then amplified through 21 cycles of PCR. Total PCR reaction volume was 301.5 nl. Each single-cell reaction was collected into dilution buffer (Fluidigm) on a 96-well plate. The final volume per cell was 23 μl. In parallel, a set of Tube Controls (TC) using mRNA from ∼200 cells (TC_200), 5 ng of purified RNA (Qiagen RNEasy Mini Kit)(TC_RNA) and a negative control (TC_NTC) were similarly processed on a BioRad T100 Thermocycler. Briefly, each single TC was lysed with 2 μl of lysis mix. Total lysis reaction volume was 3 μl. Next, mRNA was reverse transcribed using 4 μl of RT mix. Total RT reaction volume is 7 μl. Then 1 ul RT reaction was amplified through 21 cycles of PCR using 9 μl of PCR mix. Total PCR reaction volume was 10 μl. The average cDNA yield for single cells, TC200, TC_RNA and TC_NTC were 7.78, 134, 52.6 and 29.5 ng, respectively. On benchtops, cDNA samples were converted to sequence ready libraries using the Nextera XT DNA Sample Preparation (Illumina FC-131-1096) and Index Kit (Illumina FC-131-1002). Briefly, 1.25 μl containing 0.375 ng of cDNA from every sample were added to 2.5 μl Nextera Tagment DNA Buffer, 1.25 μl Nextera Amplification Tagment Mix and incubated at 55 °C for 10 min. Then 1.25 μl Nextera NT Buffer was added followed by 5 μl of the Nextera Library Amplification Mix, 1.25 μl of Nextera Index 1 primers (N701–N712) and 1.25 μl of Nextera Index 2 primers (S502–S508). The cDNA was then amplified through 12 cycles of PCR and its profile was assessed using the Caliper DNA High Sensitivity LabChip. Equal amount of tagmented cDNA was then pooled from each sample and sequenced on an Illumina HiSeq2500 with paired-end option. cDNA of bulk cells (200 cells), a positive control consisting cDNA from 5 ng of purified total RNA, and three negative controls were also obtained (two empty wells and one well containing ERCC RNA Spike-In mix only).

Sequencing runs were processed with Illumina CASAVA software. Reads were trimmed using Trimmomatic v0.32 (ref. 20), removing low-quality bases at the ends of reads (phred33<30) and clipping the first three bases in addition to Illumina adaptor sequences using palindrome mode. A sliding window quality trimming was performed, cutting once the average quality of a window of four bases fell below 30. Reads shorter than 32 bp after trimming were discarded.

The resulting high-quality RNA-Seq reads were aligned to the human reference genome build hg19 and the ERCC reference sequences simultaneously using STAR v2.3.0e (ref. 21). Uniquely mapped reads were quantified using featureCounts v1.4.4 and the Ensembl gene annotation set release 70. Read coverage along gene body was computed using RSeQC (ref. 22). Integrative Genomics Viewer (ref. 23) was used for visualization. Multiple quality control metrics (Supplementary Data) were obtained using FASTQC v0.11.2, SAMtools^24^, BEDtools^25^ and custom scripts. Bigwig tracks for visualization were generated with custom scripts, using BEDtools and UCSC tools.

### Analysis of gene expression from RNA-Seq data

Global expression changes were assessed by unsupervised hierarchical clustering of samples and PCA. To this end, expression levels were estimated using exonic reads mapping uniquely within the maximal genomic locus of each gene and its known isoforms. All UCSC genes were used for normalization; normalized (median of ratios), variance-stabilized expression values were derived using DESeq2 (ref. 26). Hierarchical clustering was performed using Pearson's correlation as the distance metric and average linkage as the agglomeration method.

To identify specific genes that changed expression upon CLaP labelling, a differential expression analysis between tagged (*n*=9) and untagged (*n*=10) cells was performed using DESeq2 (ref. 26). Statistical significance was computed using the negative binomial distribution, with the variance and the mean estimated from the data and linked by local regression^26^.

### Imaging

All samples were imaged on an Olympus IX71 microscope (Olympus Corp.) with the appropriate epifluorescence filters and a confocal module (Thorlabs Inc.), or with a FV1000 Olympus microscope. Epifluorescence widefield images were acquired with an Orca Flash 4.0 camera (Hamamatsu Photonics).

### Image Processing

The world map representation image in Fig. 3b is a mosaic of 7 × 4 × 10 bright-field images. Contrast was enhanced in Fig. 3b,c using Matlab by first calculating the morphological opening of the original image with a 5-pixel circular kernel. The result was subtracted from the original image and a colour lookup table was chosen for visualization.

### Code availability

Matlab code for cell viability quantification, hierarchical clustering of FACS data, and bright-field image contrast enhancement are provided as Supplementary Software, under the GNU General Public License (GPL).

## Additional information

**How to cite this article**: Binan, L. *et al*. Live single-cell laser tag. *Nat. Commun.* 7:11636 doi: 10.1038/ncomms11636 (2016).

## Supplementary Material

Supplementary Information Supplementary Figures 1-12, Supplementary Note 1 and Supplementary References.

Supplementary Data 1 RNA-Sequencing statistics

Supplementary Software Matlab code used for image analysis as well as LabVIEW code for microscope control
