# Supplementary material for: Live single-cell laser tag
Source: Nat Commun. 2016 May 20;7:11636. doi: 10.1038/ncomms11636 (PMC4876456; doi:10.1038/ncomms11636)
Supplement: Supplementary Information — Supplementary Figures 1-12, Supplementary Note 1 and Supplementary References. [file ncomms11636-s1.pdf]

[illegible]

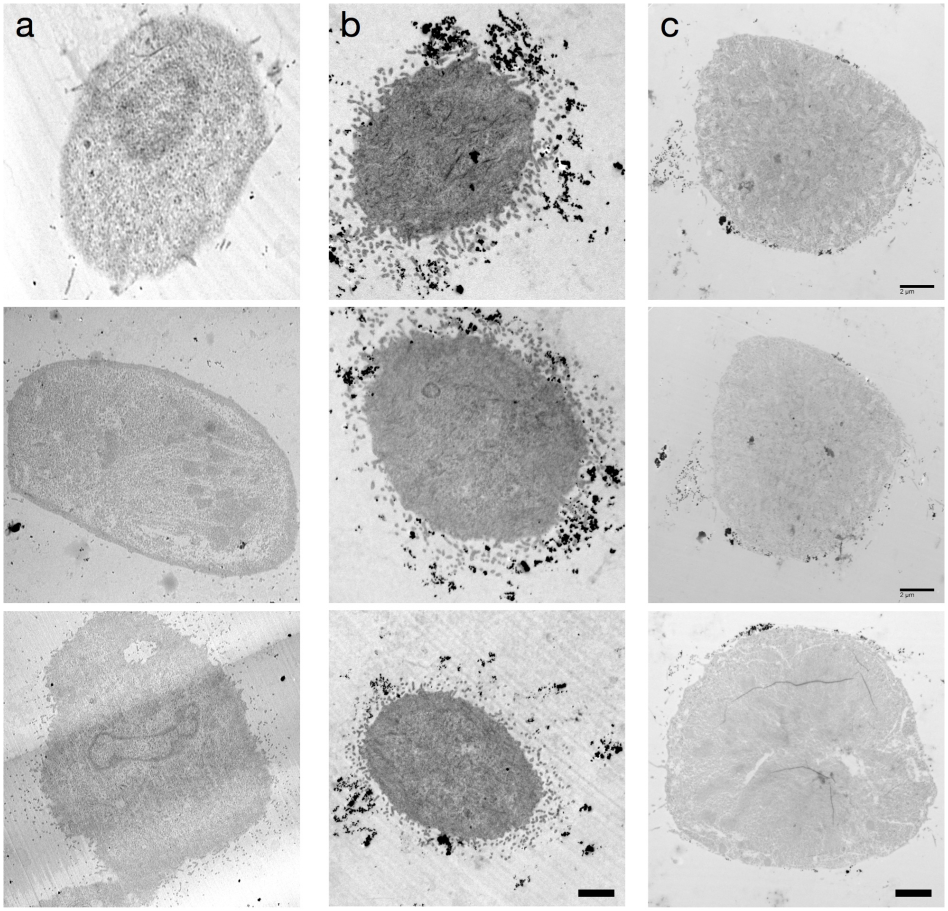

Supplementary Figure 1: Electron dense staining of isolated cells for electron microscopy imaging. CLaP was used to label cells for electron microscopy. (a) Control, non-tagged cells. (b) Positively stained cells with streptavidin-HRP are surrounded by a number of dark DAB precipitates, close to their filopodia where we expect the streptavidin HRP to be bound. (c) In an independent experiment, we used silver-enhanced 6nm immunoGold-Streptavidin to reveal cells. Scale bars: 2µm.

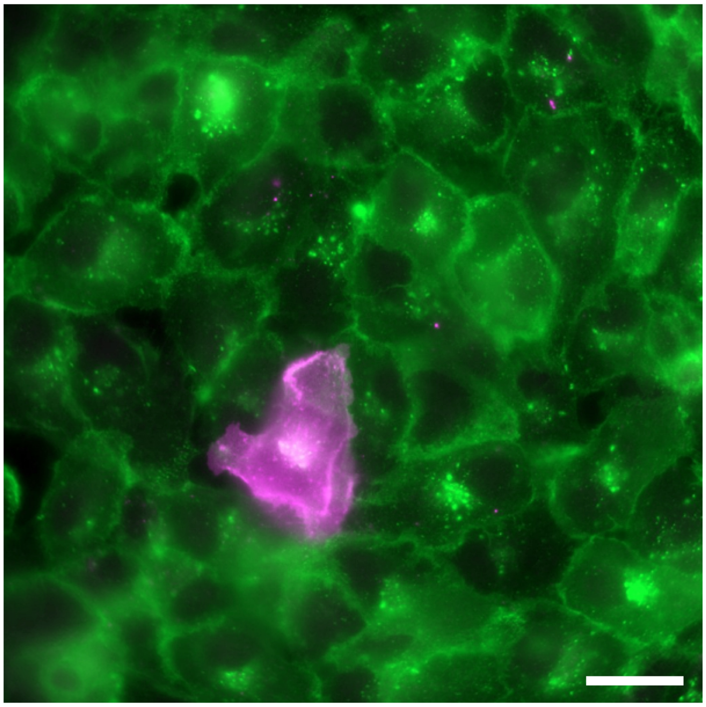

Supplementary Figure 2: CLaP tags are resistant to routine cell culture procedures. In order to test whether biotin tags withstand routine cell culture protocols, we illuminated individual ARPE-19 cells to crosslink biotin on their membrane. We then incubated cells in trypsin to detach them from the substrate. Cells were then plated in a new dish and let overnight in an incubator before adding Streptavidin-Alexa-647 to the medium and imaging on the following day. Isolated labeled cells (magenta) were readily identified among the large population of untagged cells stained with WGA (green). Scale bar, 20 µm.

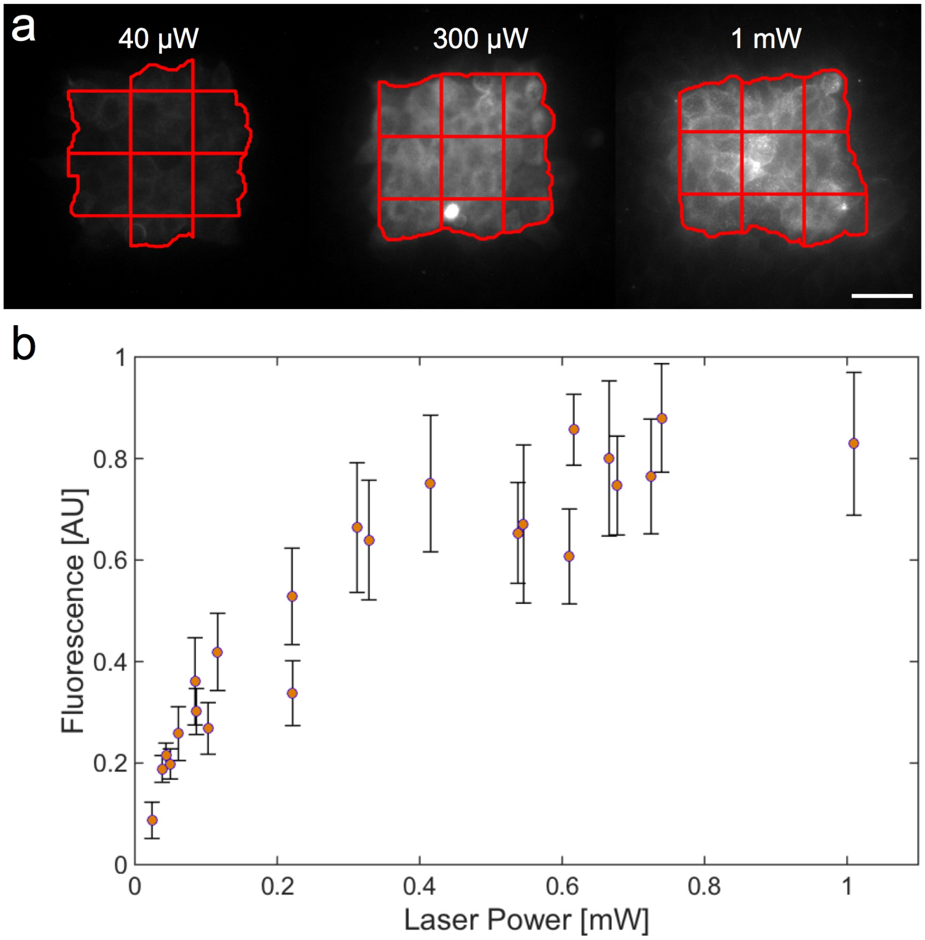

Supplementary Figure 3: Laser power calibration. (a) To calibrate the fluorescence intensity obtained with CLaP, we tagged square regions (100×100 μm^2) of MDCK cells using a 0.4 NA objective, scanning the sample at 170 μm/s, and using different laser intensities in each square: 40 μW, 300 μW and 1 mW. Laser intensity was measured at the objective focal plane and monitored at 10 Hz in order to control for fluctuations. After washing and incubating with Alexa-647-Streptavidin, we imaged each square region using epifluorescence with a 40XNA0.95 objective and 10s exposure time for all square regions. We chose objective magnification and spacing between squares to avoid photobleaching nearby squares during imaging. For similar reasons, the microscope field of view was placed around each square using prior knowledge of the pattern location, and focusing was done using brightfield illumination. Fluorescence intensity quantification of CLaP tagged cells was automated using Matlab (MathWorks) to avoid subjective bias. A binary mask was created using the Otsu[1] algorithm followed by a morphological opening (using a disk shaped structuring element of 5 μm radius), and a hole filling operation. Only the largest object in the binary image was kept. To assess fluorescence fluctuations within each pattern, the mask was subdivided in smaller square regions of 40×40 μm^2 as shown. Subregions smaller than 500μm^2 were discarded from the analysis. Scale bar, 40 μm. (b) Fluorescence within each pattern was characterized as a function of laser power. Markers and bars indicate the median and standard deviation of CLaP fluorescence of square regions.

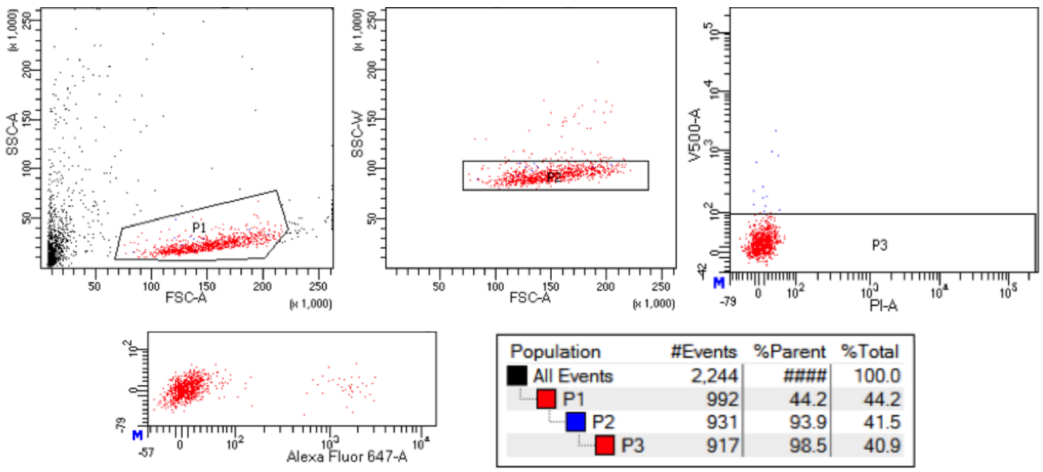

Population	#Events	%Parent	%Total
All Events	2,244	####	100.0
P1	992	44.2	44.2
P2	931	93.9	41.5
P3	917	98.5	40.9

Supplementary Figure 4: Gatings for fluorescence activated cell sorting. Three standard gates were defined to count exclusively events originated from isolated viable cells. Gate P1 was built from the plot of side-scattered light (SSC) peak area vs. forward-scattered light (FSC) peak area, for discriminating whole cells from other particles and debris. A second gate (P2), used to discard doublets and other clusters, was defined using the graph of SSC peak width vs. FSC peak area. Finally, dead cells were filtered out with gate P3, which was defined from the peak area of the v500 channel (v500-A) which measured SYTOX Blue labeling.

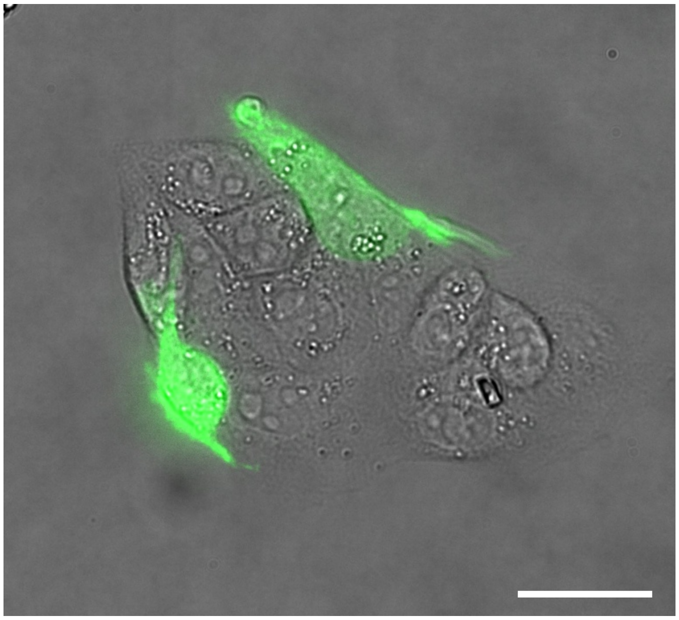

Supplementary Figure 5: mixed cell culture for single-cell microfluidics and capture. 3T3 fibroblasts expressing mNeonGreen (green), and non-fluorescent MDCK cells were co-cultured. Image illustrates mixed cell clusters with cell types that can be visually distinguished. Only MDCK cells (non-fluorescent in the image) were targeted by CLaP in subsequent experiments. Scale bar 20μm.

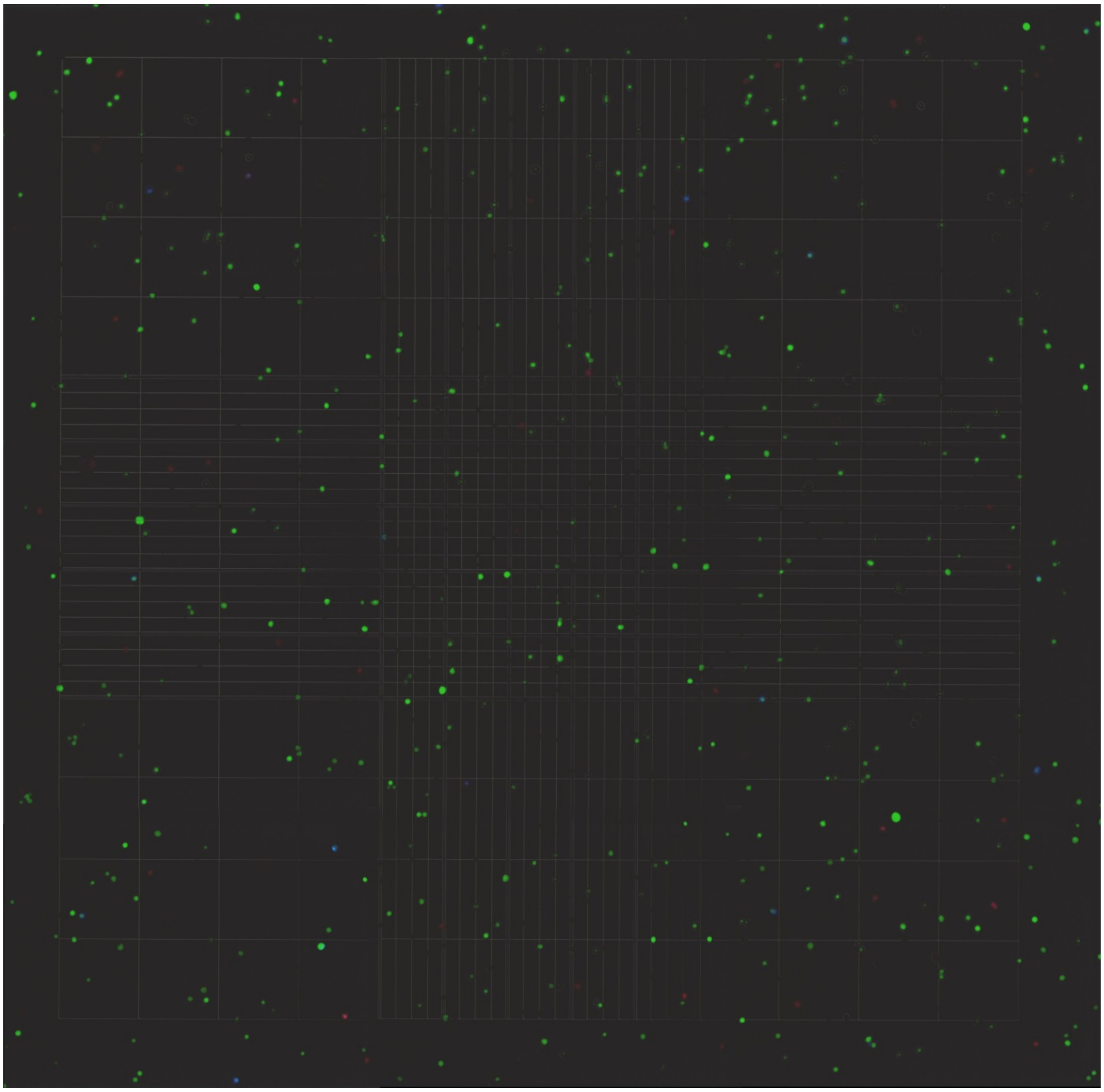

Supplementary Figure 6: Viability assay prior to C1 chip sorting. After CLaP, mixed cells were suspended using EDTA 10 mM and loaded in a C1 Fluidigm chip to isolate CLaP tagged cells from the rest. Live cells appear in green, dead cells appear in red, and alexa-647 streptavidin positive cells appear in blue.

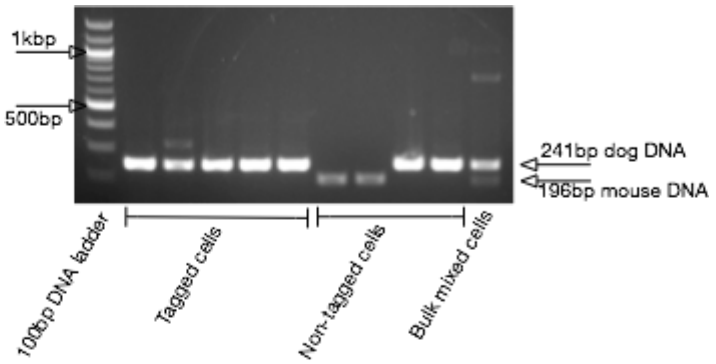

Supplementary Figure 7: Identification of species of origin in co-culture experiment. Cells from different species were co-cultured, tagged, isolated and analyzed by PCR. Here, the complete gel corresponding to figure 3c in the main text is shown, including molecular markers.

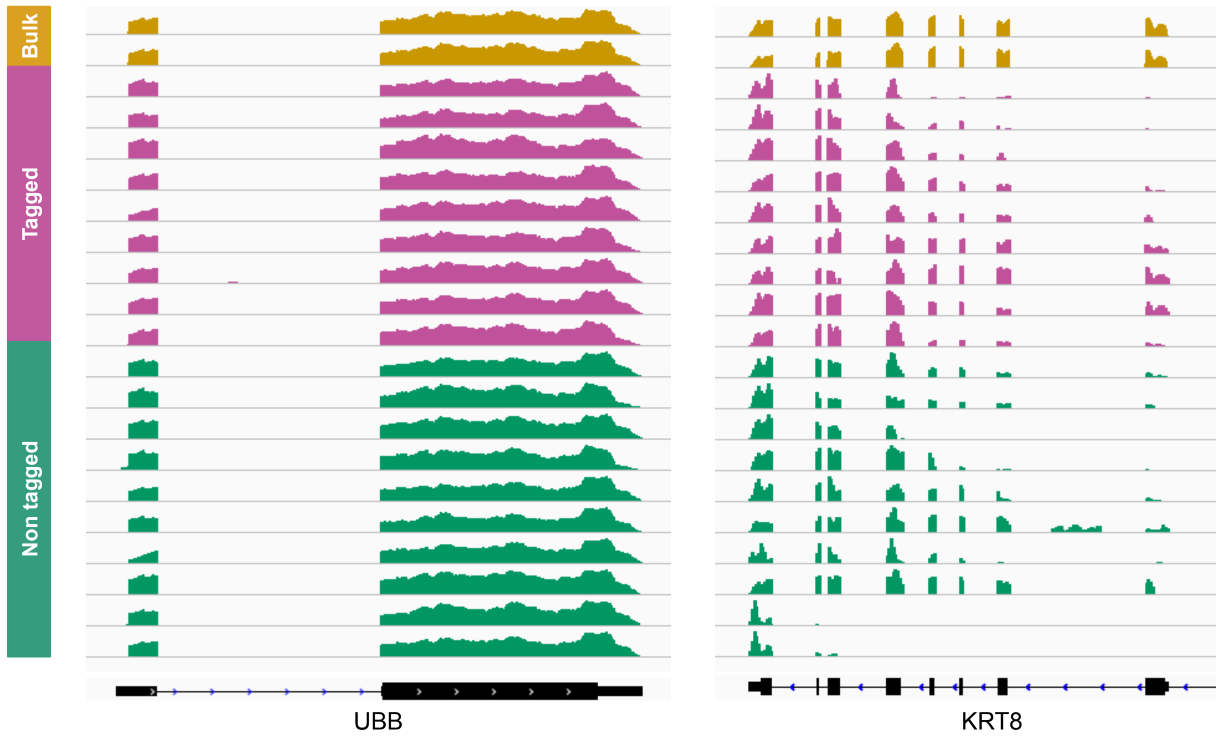

Supplementary Figure 8: Single-cell transcriptomic analysis. RNA-Seq data for one highly expressed gene (*UBB*) and one RPE marker (*KRT8*) from bulk (yellow), tagged single-cells (magenta) and non-tagged single cells (green).

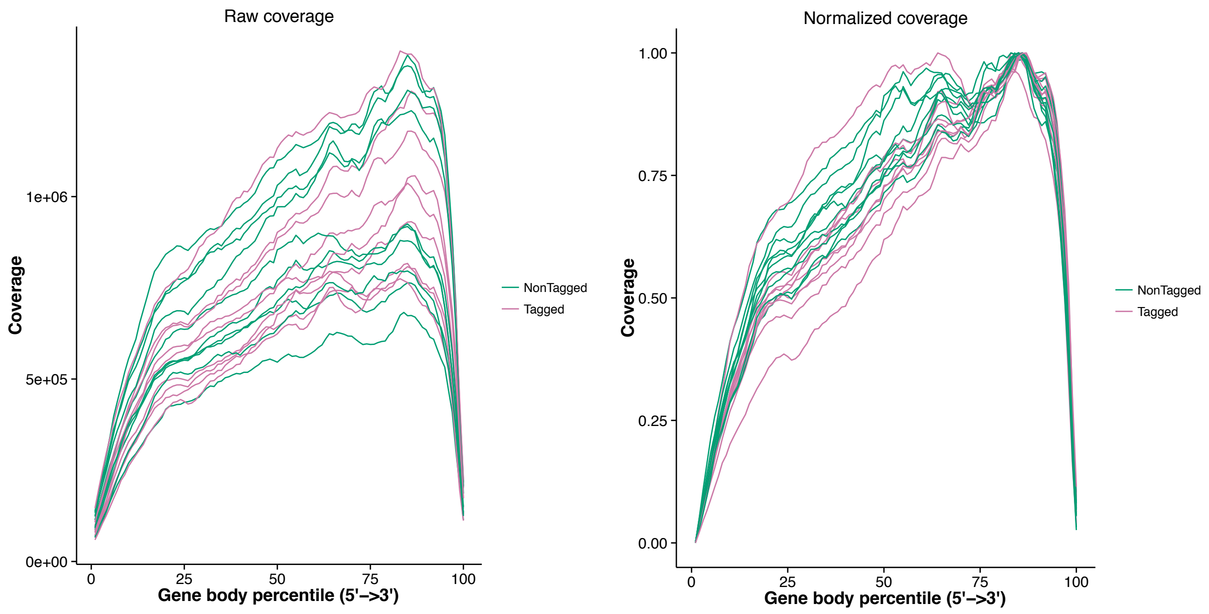

Supplementary Figure 9: Raw and normalized coverage uniformity over gene body. Using RSeQC (Bioinformatics 28 (16:2184-5), 2012), all transcripts were scaled to 100 nucleotides and the number of reads covering each nucleotide position was computed. The slight 3' bias observed is expected, since it has previously been reported for SMARTer Ultra Low RNA kit.

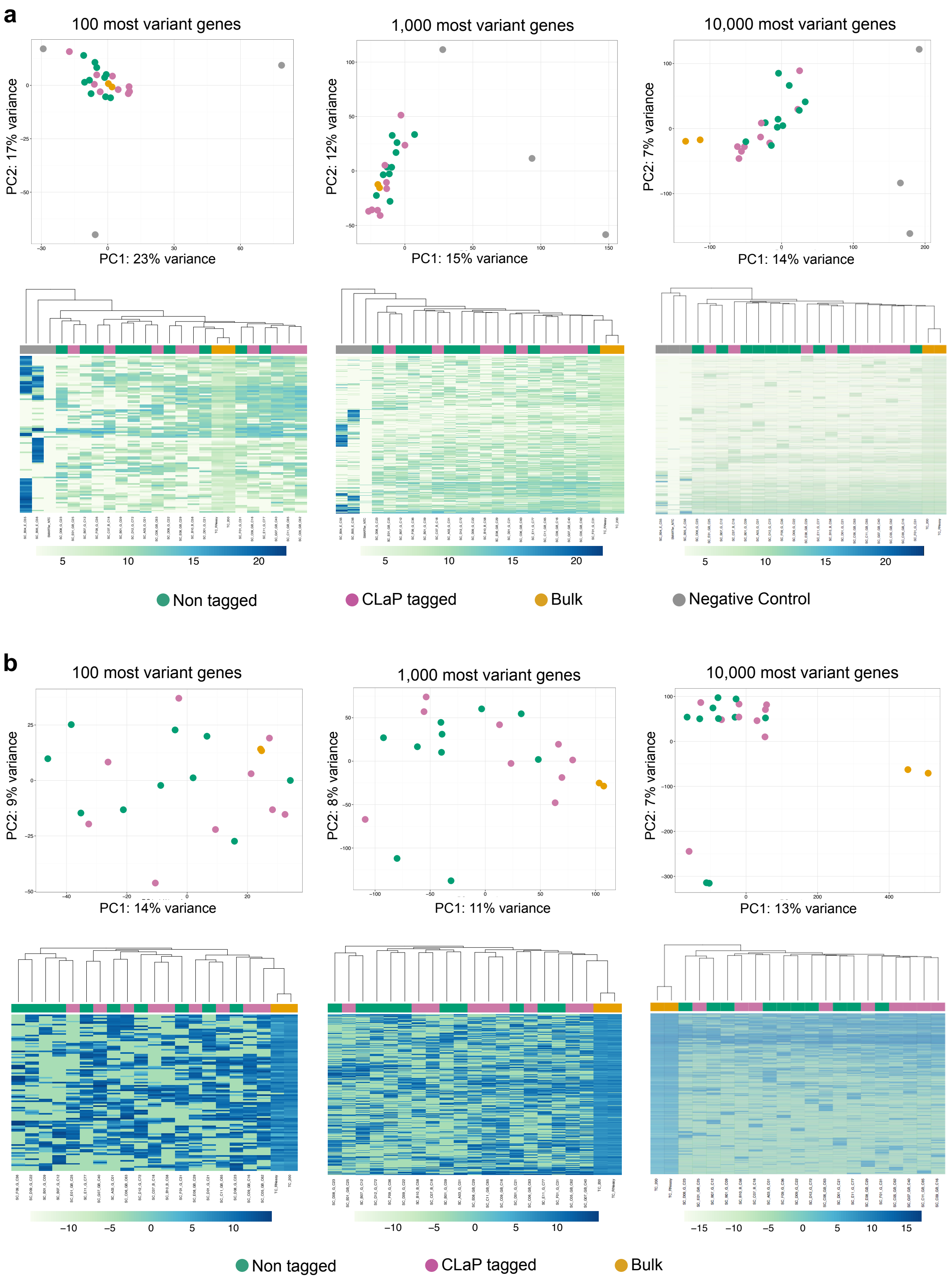

Supplementary Figure 10: Effects of CLaP on gene expression. Global effects of CLaP on cells were evaluated by unsupervised clustering of samples based on expression profiles, using

variable number of genes (from 100 to 10,000). Clustering was performed either including (a) or excluding (b) negative controls. Tagged and untagged cells are consistently clustered together, indicating no major expression changes associated with the procedure.
Bulk: cDNA synthesized from 5 ng of purified total RNA derived from cells before capture in the C1 system, and a bulk cell control derived from approximately 20 lysed cells also before capture in the C1 system.
Negative controls: libraries were prepared from cDNA synthesized from samples corresponding to two empty (no cell captured) positions in the C1 chip, producing low yield of cDNA (<0.5ng), as well as an ERCC spike only cDNA synthesis control.

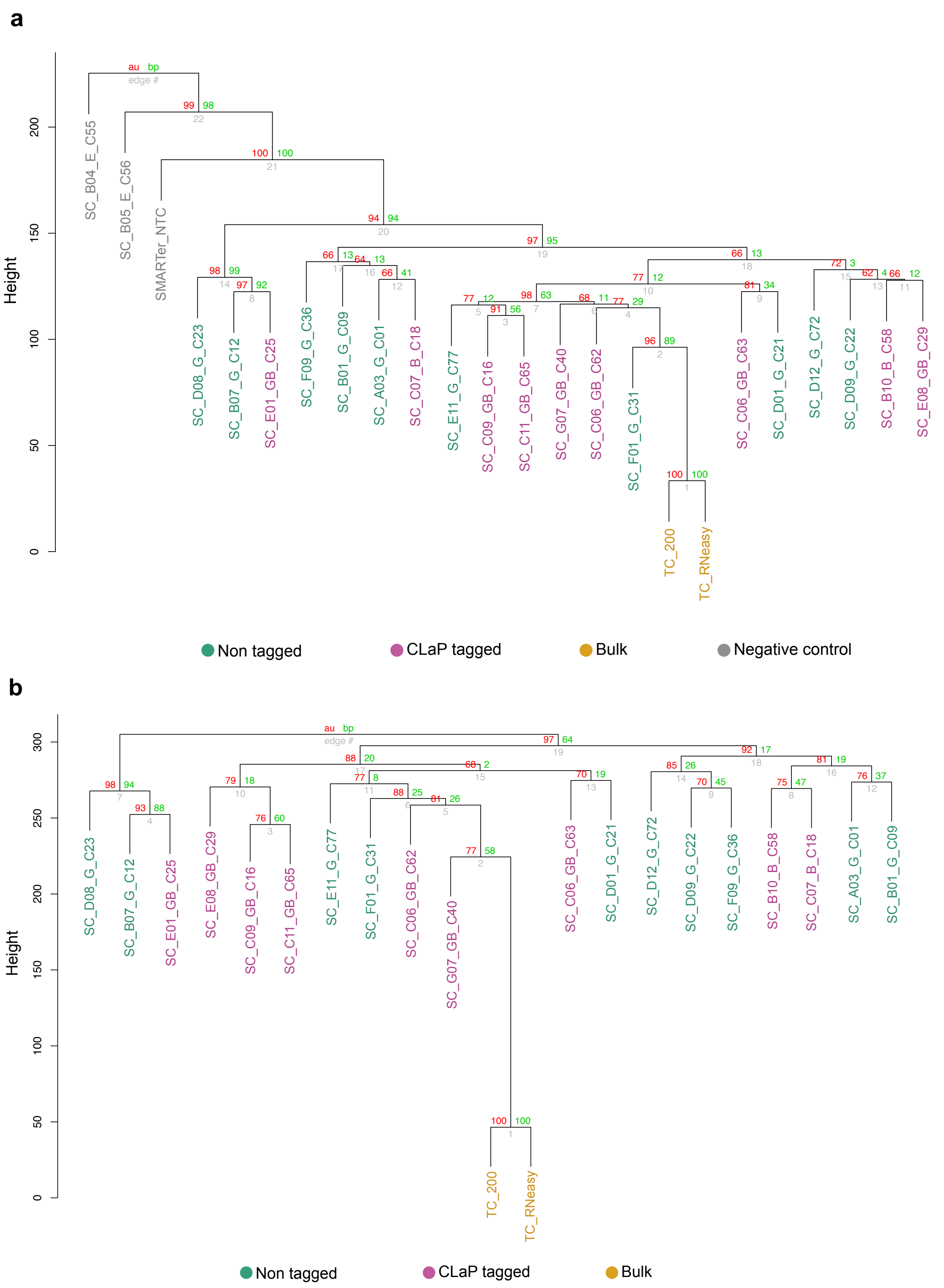

Supplementary Figure 11: Multiscale bootstrapping of gene expression clustering, performed with the R package pvclust (Bioinformatics 22(12:1540-2), 2006). In red, the approximately unbiased (AU) p-value is represented. Bootstrapping was performed using the 1,000 most variant genes, including (a) or excluding (b) negative control samples.

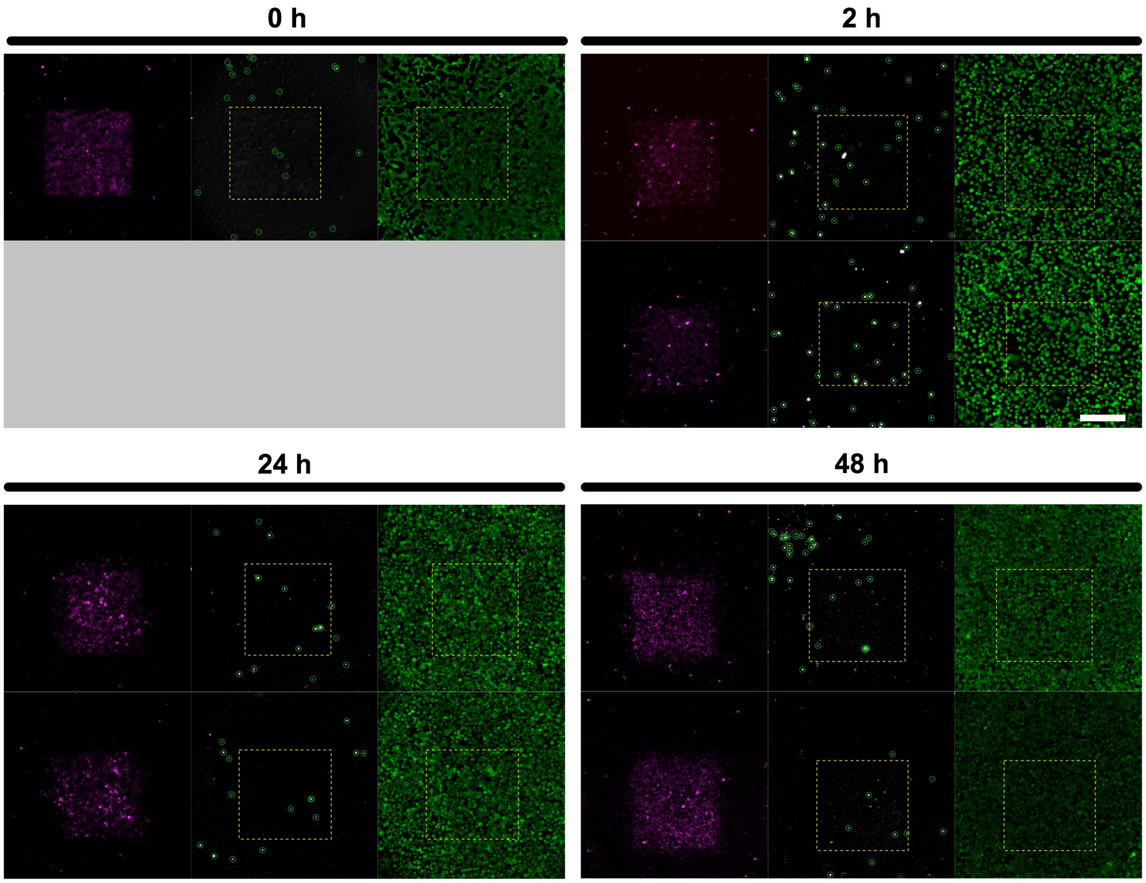

Supplementary Figure 12: Complementary viability analysis. As a complement for the viability study shown in Fig. 2c in the main text, we provide here the complete series of images used for quantification. At each time point, two samples were analyzed, except for 0h. For each sample we show three images (from left to right): Streptavidin-Alexa647 expression, PI expression and detections by the cell-counting algorithm, and Calcein AM expression. The rectangular regions displayed with dotted yellow lines represent the region subjected to CLaP, automatically segmented from the streptavidin-ALexa647 channel. Scale bar: 300μm.

Supplementary Note 1: Photobleaching based crosslinking

As opposed to other studies that require the formation of nitrene groups upon UV illumination[2], crosslinking in CLaP is based on photobleaching fluorescein to create free radicals. The process that leads to the creation of free radicals using fluorescein has been extensively described by Song et al.[3,4]. Briefly, upon excitation by light absorption, molecules reach a singlet-excited state, which decays emitting fluorescence. These singlet-excited molecules can cross to an unstable triplet-excited state, causing photobleaching. When two triplet-sate molecules react, they produce semi oxidized and semi reduced radical forms of fluorescein. Both of these molecules have free electrons and are extremely unstable, reacting with molecules in their close vicinity to form stable compounds. Fluorescein is of particular interest for this specific use as it has a very low resistance to photobleaching and requires low illumination power to obtain free radicals[5,6]. Biotin-4-fluorescein molecule has a short spacer arm between fluorescein and biotin, which allows the biotin to fit the binding pocket of streptavidin despite the presence the fluorescein tag.

Supplementary References

1 Otsu, N. Threshold Selection Method from Gray-Level Histograms. *Ieee T Syst Man Cyb* 9, 62-66 (1979).

2 Brunner, J. New photolabeling and crosslinking methods. *Annual review of biochemistry* 62, 483-514, doi:10.1146/annurev.bi.62.070193.002411 (1993).

3 Song, L., Hennink, E. J., Young, I. T. & Tanke, H. J. Photobleaching kinetics of fluorescein in quantitative fluorescence microscopy. *Biophys J* 68, 2588-2600, doi:10.1016/S0006-3495(95)80442-X (1995).

4 Song, L., Varma, C. A., Verhoeven, J. W. & Tanke, H. J. Influence of the triplet excited state on the photobleaching kinetics of fluorescein in microscopy. *Biophys J* 70, 2959-2968, doi:10.1016/S0006-3495(96)79866-1 (1996).

5 Keppler, A., Arrivoli, C., Sironi, L. & Ellenberg, J. Fluorophores for live cell imaging of AGT fusion proteins across the visible spectrum. *Biotechniques* 41, 167-170, 172, 174-165 (2006).

6 Sobek, J., Aquino, C. & Schlapbach, R. *Analyzing Properties of Fluorescent Dyes Used for Labeling DNA in Microarray Experiments*. Vol. 2.5 (2007).
