# Supplementary material for: Live single-cell laser tag
Source: Nat Commun. 2016 May 20;7:11636. doi: 10.1038/ncomms11636 (PMC4876456; doi:10.1038/ncomms11636)
Supplement: Supplementary Software — Matlab code used for image analysis as well as LabVIEW code for microscope control [file ncomms11636-s3.zip › code/ClapAutomationSoftware/readme.pdf]

ClaP automation Software

We developed software in NI Labview to help performing the most typical ClaP operations:

- Single cell tagging and Imaging
- Bulk tagging

All tasks related with each technique are grouped in libraries (llb files), and there is a separate library (library.llb) that holds shared subVIs. This software has been developed for our specific hardware setup and it is provided here as a guide. It will need heavy modifications to make it work in other platforms.

Single cell ClaP

The software for choosing, tagging, and imaging single cells in several epifluorescence channels is grouped in the library "singleCellClaP.llb".

Single cell Tagging

This is performed by singleCellTag.vi which front panel is shown in Fig. 1.

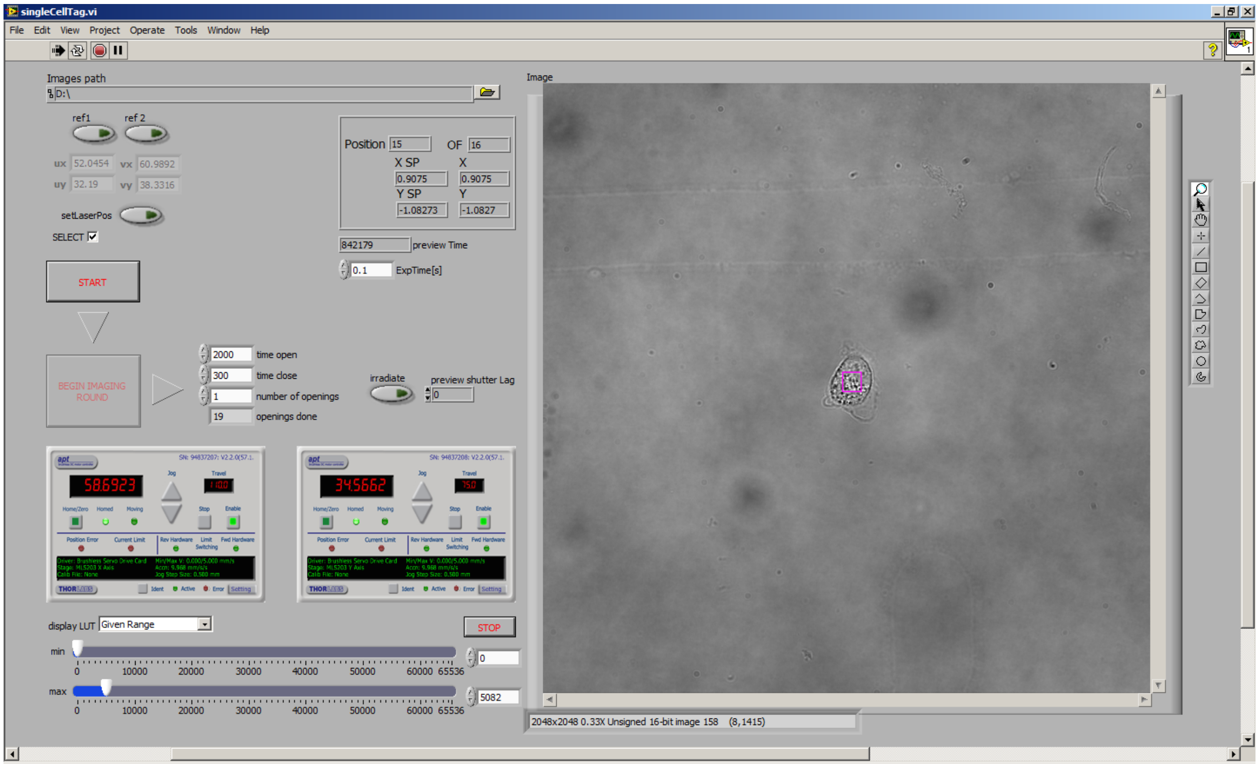

Figure 1. Front panel for single cell tagging VI.

This program controls the microscope camera (Hamamatsu ORCA-flash4.0), the microscope stage motors (Thorlabs APT platform), and the shutter that blocks the tagging laser through a TTL signal generated by a USB Multifunction DAQ (LabJack Corporation). After clicking the Start button, the application allows the user to move around the sample and choose specific cells to tag. To do this a subVI called "Spots selector" (Fig. 2) is displayed.

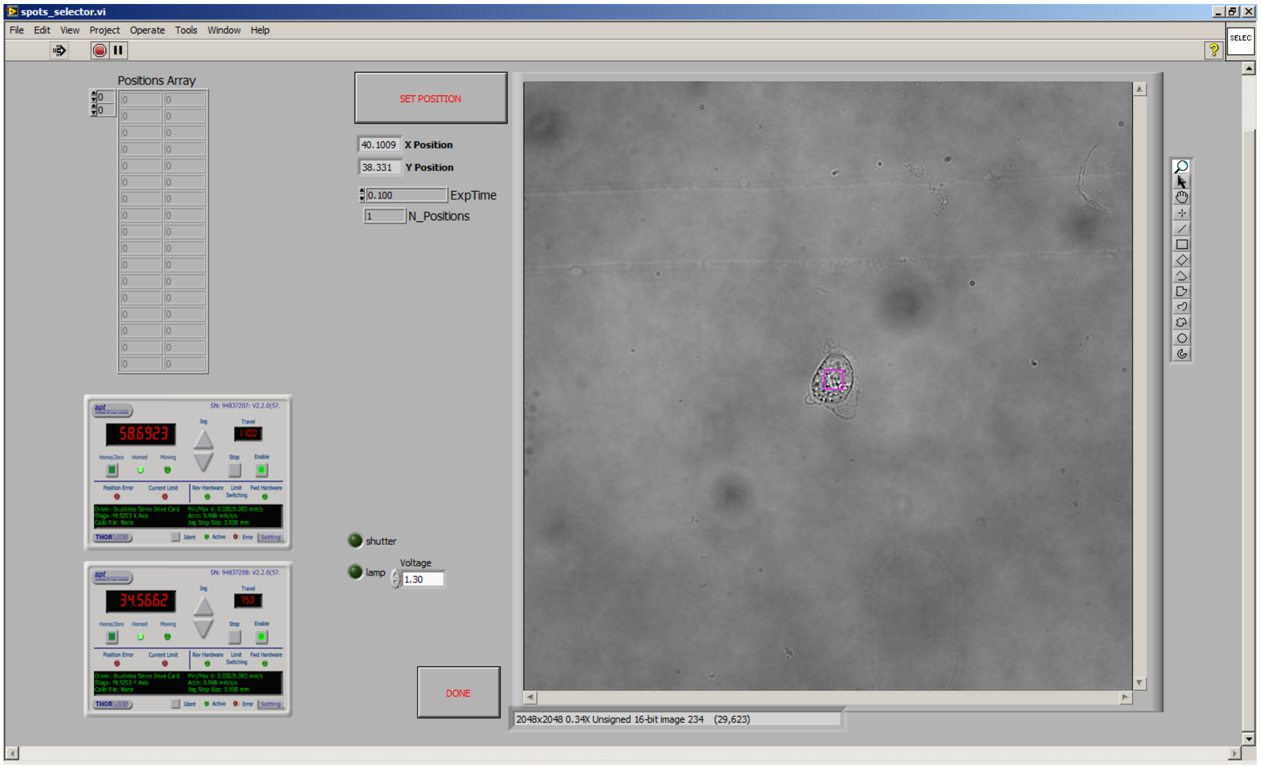

Figure 2. Front panel of the "spots_selector" subVI.

Once on this panel, the user can move the sample until the ROI rectangle in the image is located at the center of the target cell. By clicking the "Set position" button, the location of the sample is stored in the file "positions.txt". After selecting the set of cells to ClaP, the user can exit this panel by pressing the "Done" button, going back to the front panel of singleCellTag.vi. At this stage, the "Begin tagging round" button is enabled, and after clicking on it, the stage moves to place the first target cell to the laser target. There the user is allowed to focus the sample on the desired plane, and click the "irradiate" button. The irradiation conditions can be set on the edit boxes just left to the button. Once the irradiation of the cell has finished, the stage moves to the next position.

The "set laser pos" button is used before clicking start, to register the location of the ROI rectangle on the image. This is typically done by irradiating (and photobleaching) a thin layer of dye, and moving and resizing the rectangle until it overlaps with the region photobleached by the laser. By clicking the "set laser pos" button the coordinates and size of the region is stored in the "roi.xml" file, within the "images path".

Similarly the buttons “ref1” and “ref2” are used to register the stage coordinates of two points fixed to the sample, in order to be able to remove the sample from the stage, and finding the cells position back, at a later time. This is useful for imaging the cells after incubation steps performed outside the microscope. This step is performed before clicking the start button. Practical reference points are two diagonal corners of the coverglass glued to bottom of a MatTek dish.

Imaging cells

This is performed by, either “imagingCells1Channel.vi”, “imagingCells2Channel.vi”, and “imagingCells3Channel.vi” (Front panels not shown), depending on how many channels are needed to image.
This VI works similarly to singleCellTag.vi. Before running, the images path has to be set to the same where the “roi.xml” and “postions.txt” files are stored. The references ref1 and ref2 have to be set before clicking start. After clicking the start button, the stage moves to the first stored cell position, and allows focusing and adjusting the camera parameters. Images are captured by clicking on the corresponding “Image” button, and stored under the “images path”.

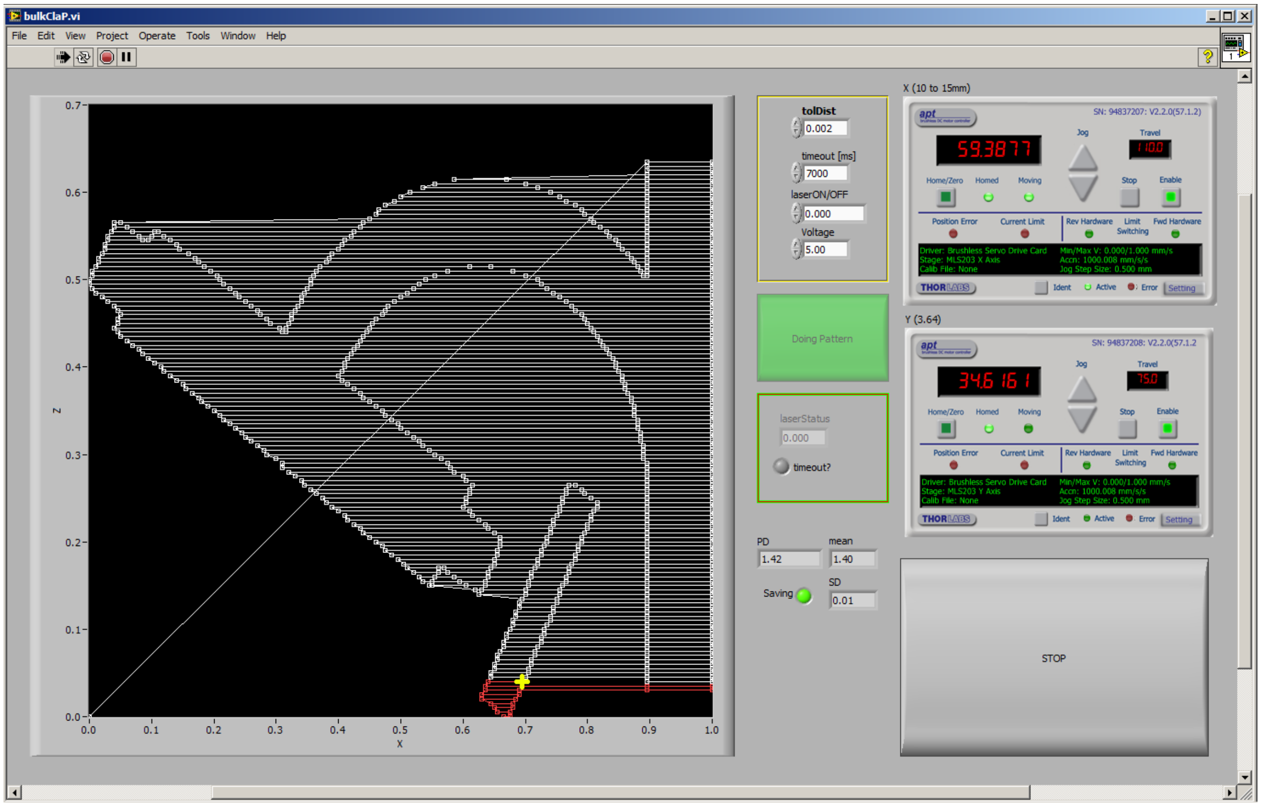

Figure 3. Front panel of the “BulkClap” VI.

Bulk tagging

The software for performing bulk irradiations is grouped in the library “bulkClaP.llb”. The VI “bulkClap.VI”, inside the library, controls the laser shutter and the microscope stage motors. It requires a set of instructions for irradiating lines along the sample, at specific positions and motor speeds. As an example, we provide the instructions to produce the pattern of Fig. 1b of the main manuscript, in the text file “simpleMicroscopeMotors.txt”. Each row in the text file corresponds to a line. The columns correspond to:

X target position	Y target position	Laser TTL	X speed [mm/s]	Y speed [mm/s]

Each time the software runs, the “select file dialog” is displayed for browsing to the instructions file. The pattern is irradiated after clicking the start button. The front panel of this piece of software is shown in Fig. 3.
