# Supplementary figures and images for: Live single-cell laser tag
Source: Nat Commun. 2016 May 20;7:11636. doi: 10.1038/ncomms11636 (PMC4876456; doi:10.1038/ncomms11636)

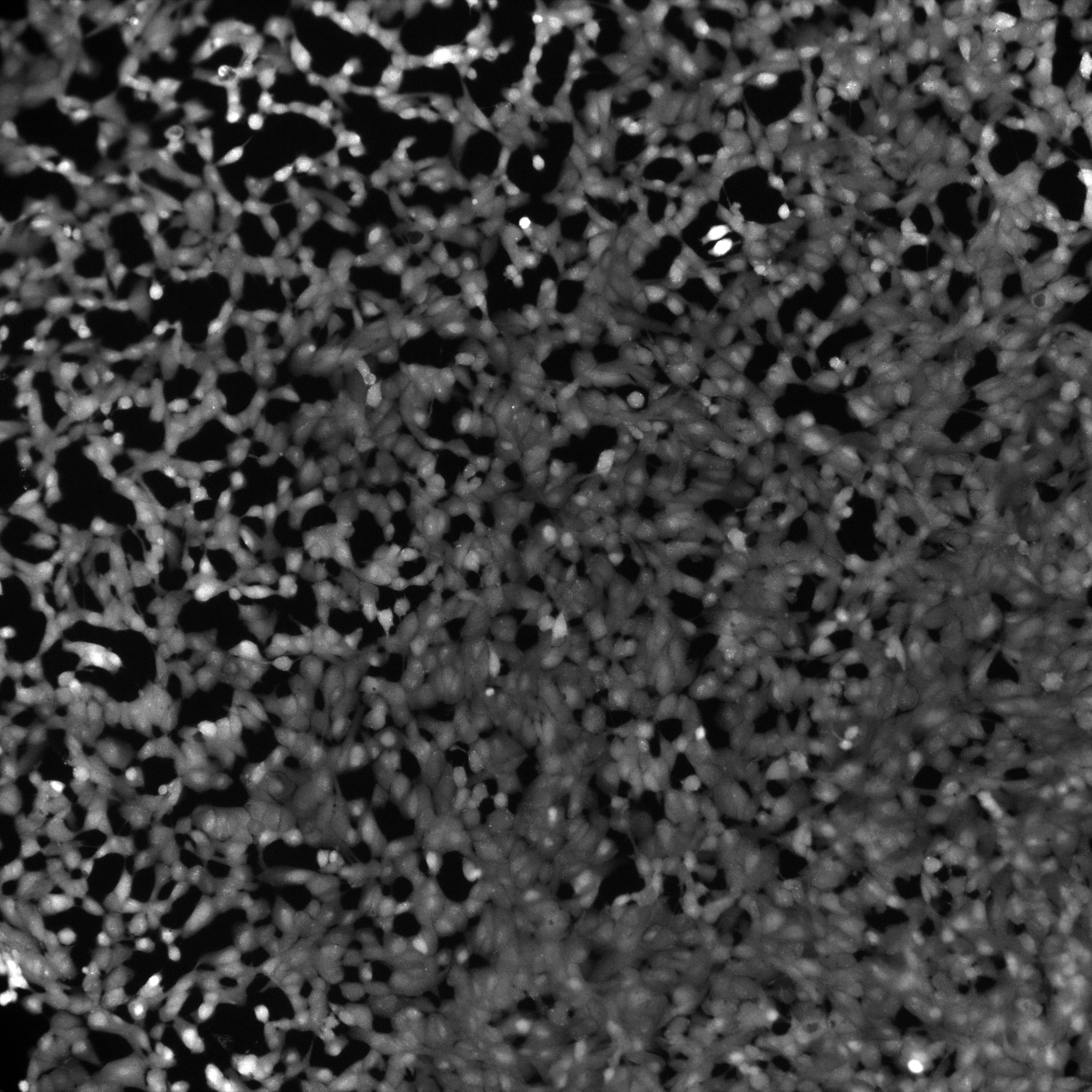

Supplement: Supplementary Software — Matlab code used for image analysis as well as LabVIEW code for microscope control [file ncomms11636-s3.zip › code/Viability/images/TIME00H_FOV0_CAL.tif]

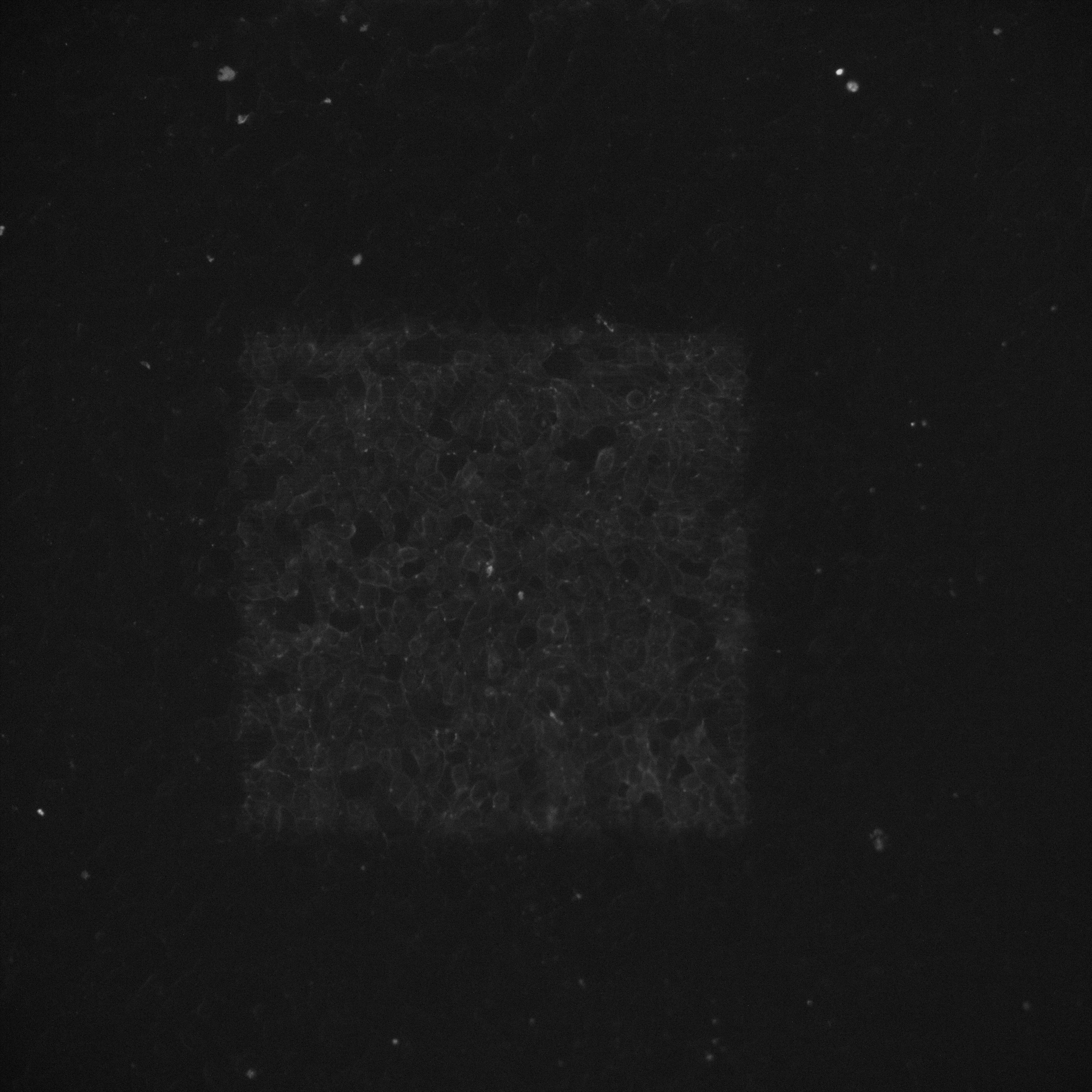

Supplement: Supplementary Software — Matlab code used for image analysis as well as LabVIEW code for microscope control [file ncomms11636-s3.zip › code/Viability/images/TIME00H_FOV0_CY5.tif]

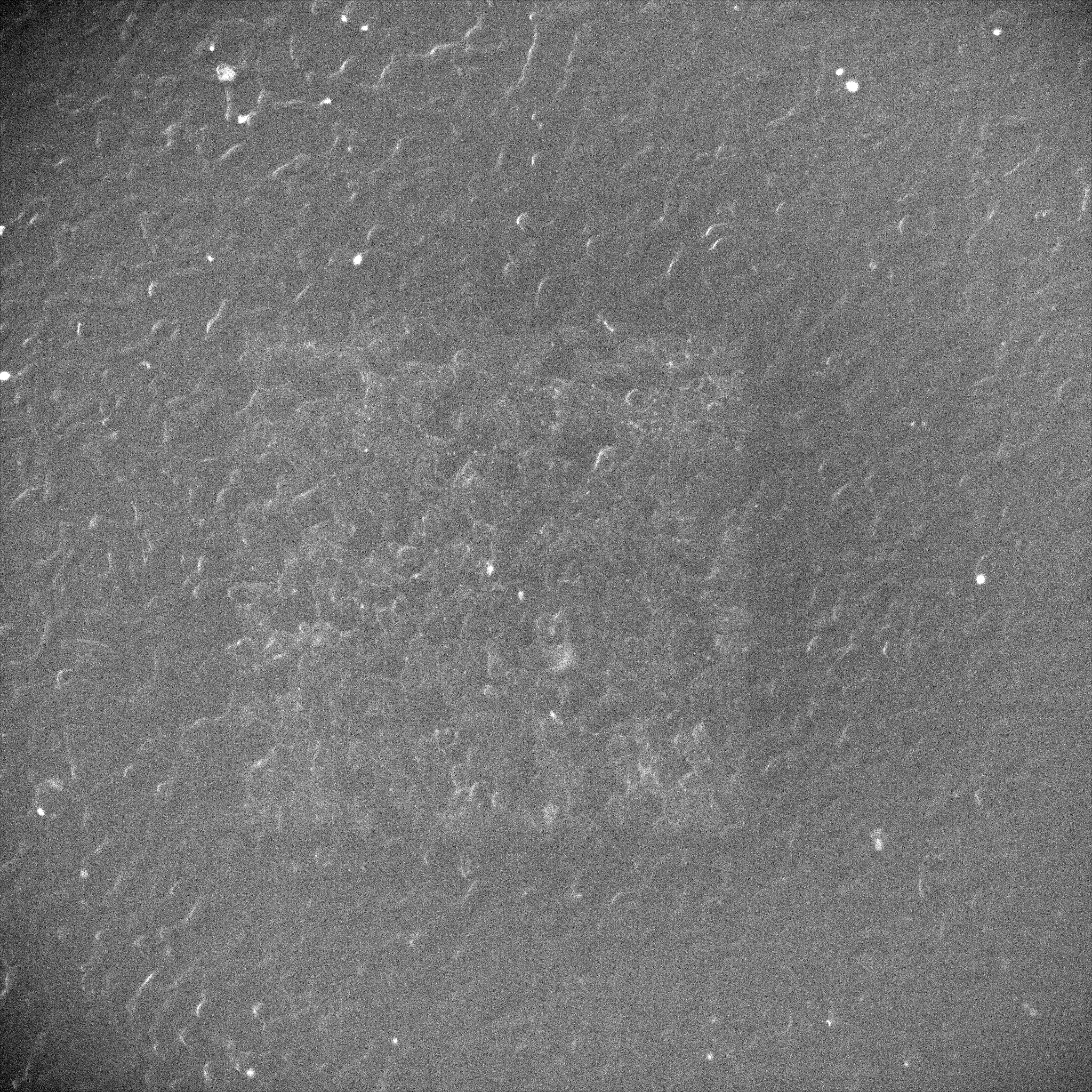

Supplement: Supplementary Software — Matlab code used for image analysis as well as LabVIEW code for microscope control [file ncomms11636-s3.zip › code/Viability/images/TIME00H_FOV0_PI.tif]

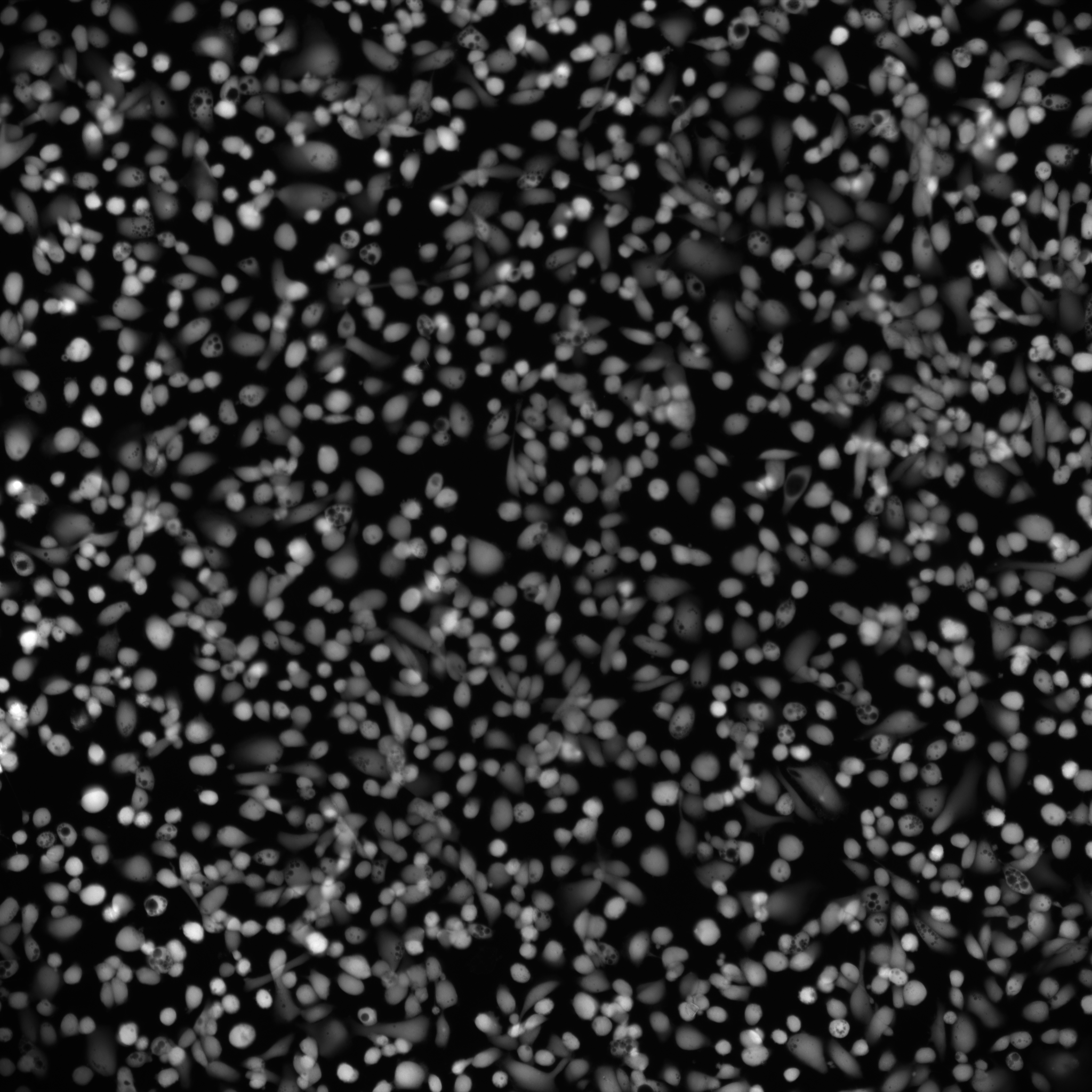

Supplement: Supplementary Software — Matlab code used for image analysis as well as LabVIEW code for microscope control [file ncomms11636-s3.zip › code/Viability/images/TIME02H_FOV0_CAL.tif]

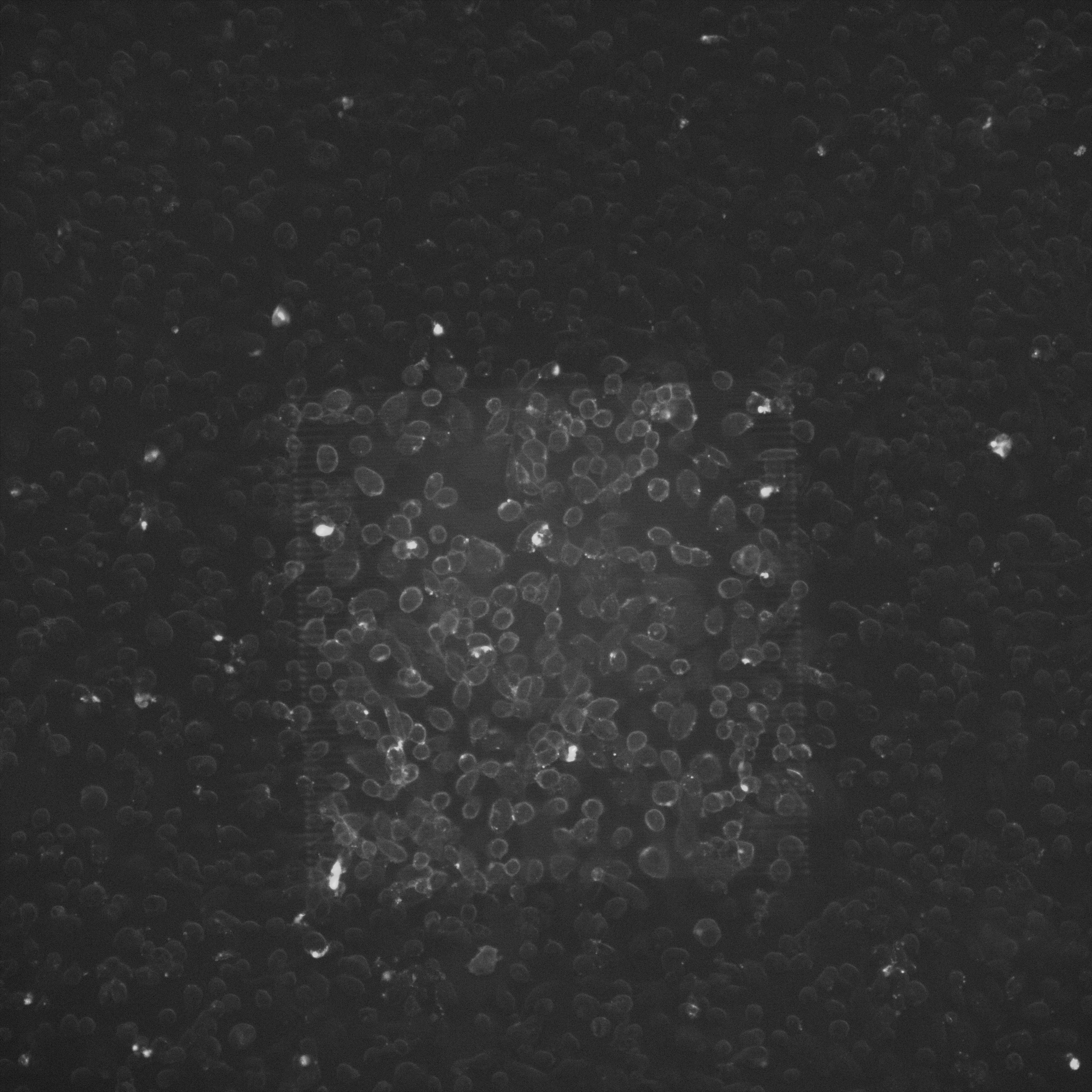

Supplement: Supplementary Software — Matlab code used for image analysis as well as LabVIEW code for microscope control [file ncomms11636-s3.zip › code/Viability/images/TIME02H_FOV0_CY5.tif]

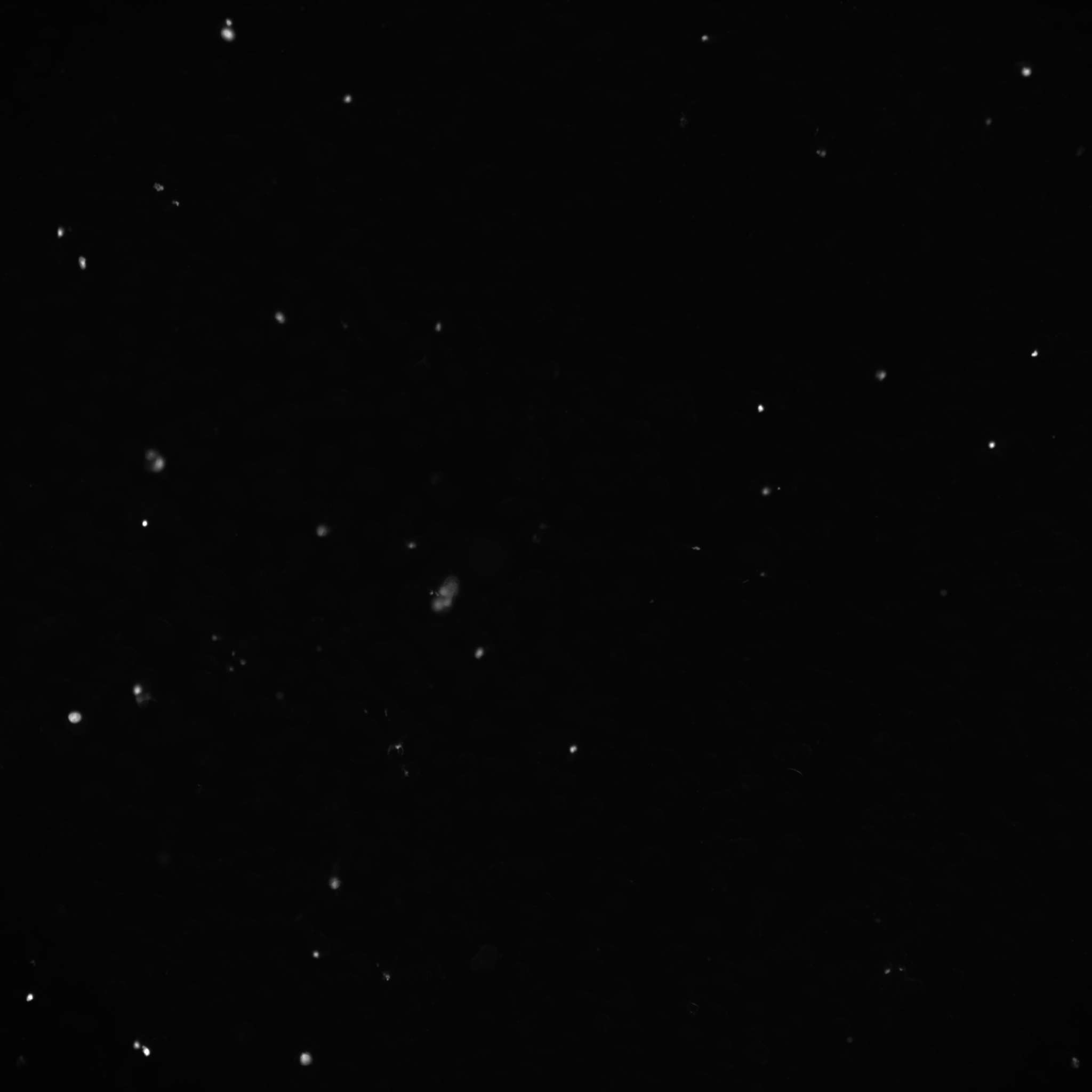

Supplement: Supplementary Software — Matlab code used for image analysis as well as LabVIEW code for microscope control [file ncomms11636-s3.zip › code/Viability/images/TIME02H_FOV0_PI.tif]

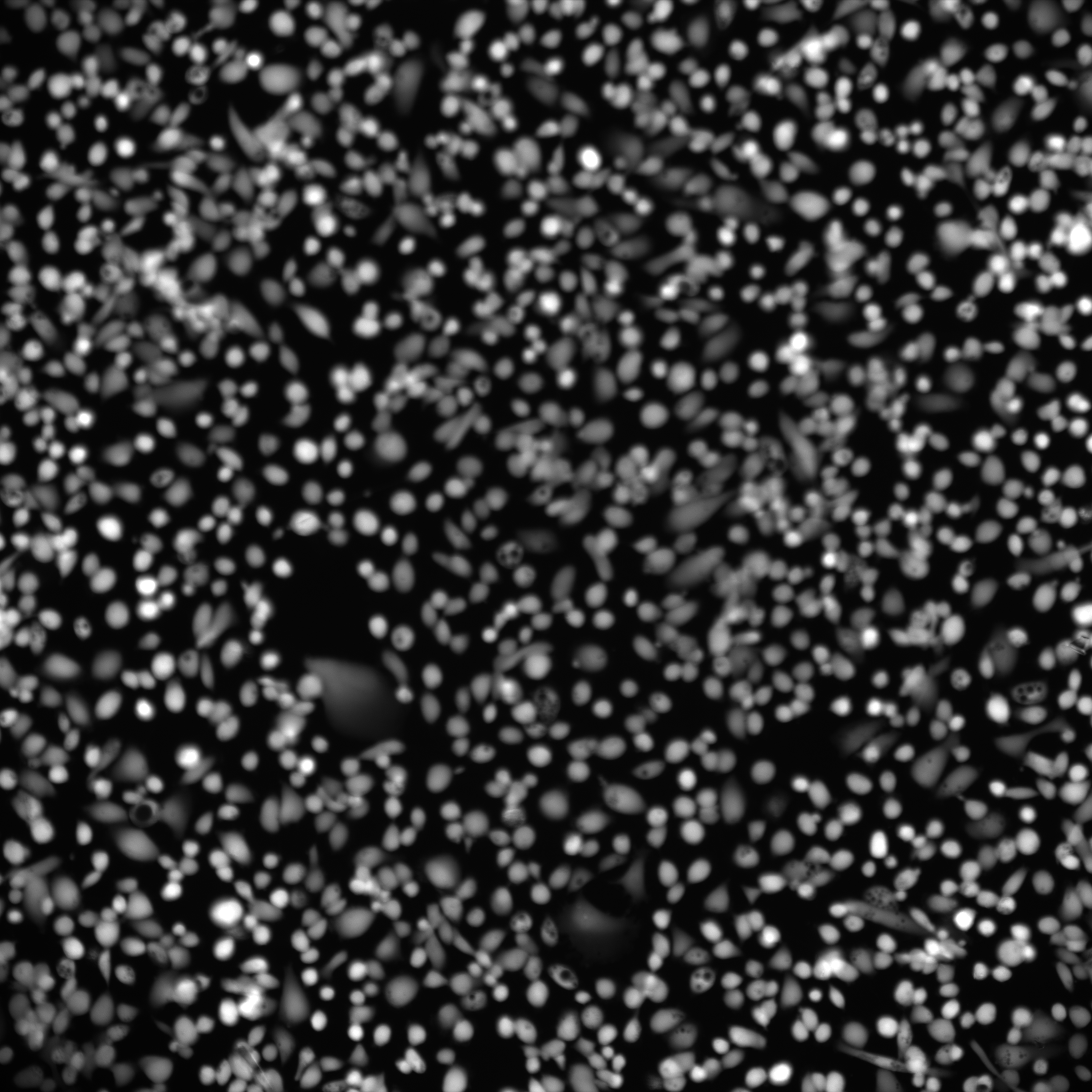

Supplement: Supplementary Software — Matlab code used for image analysis as well as LabVIEW code for microscope control [file ncomms11636-s3.zip › code/Viability/images/TIME02H_FOV1_CAL.tif]

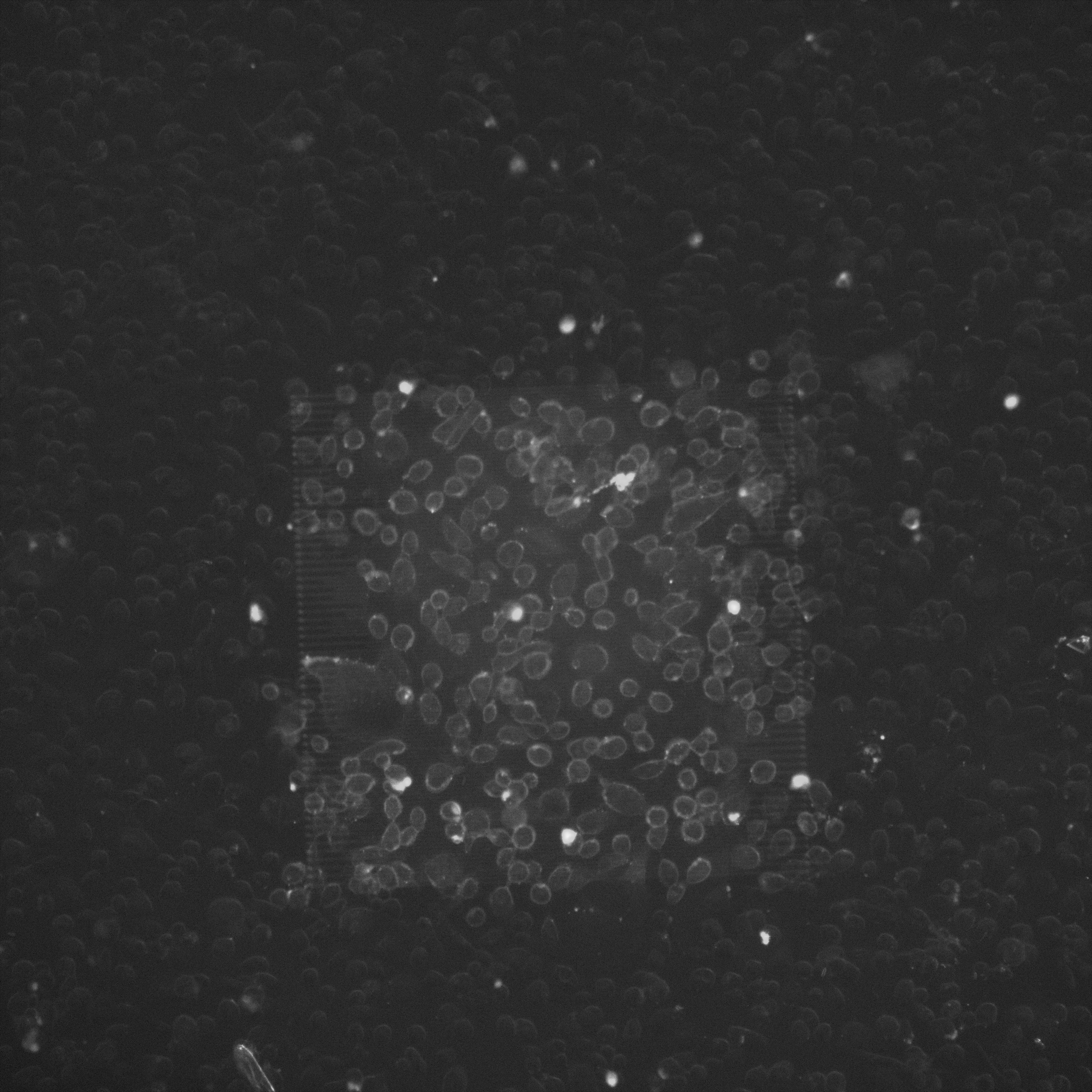

Supplement: Supplementary Software — Matlab code used for image analysis as well as LabVIEW code for microscope control [file ncomms11636-s3.zip › code/Viability/images/TIME02H_FOV1_CY5.tif]

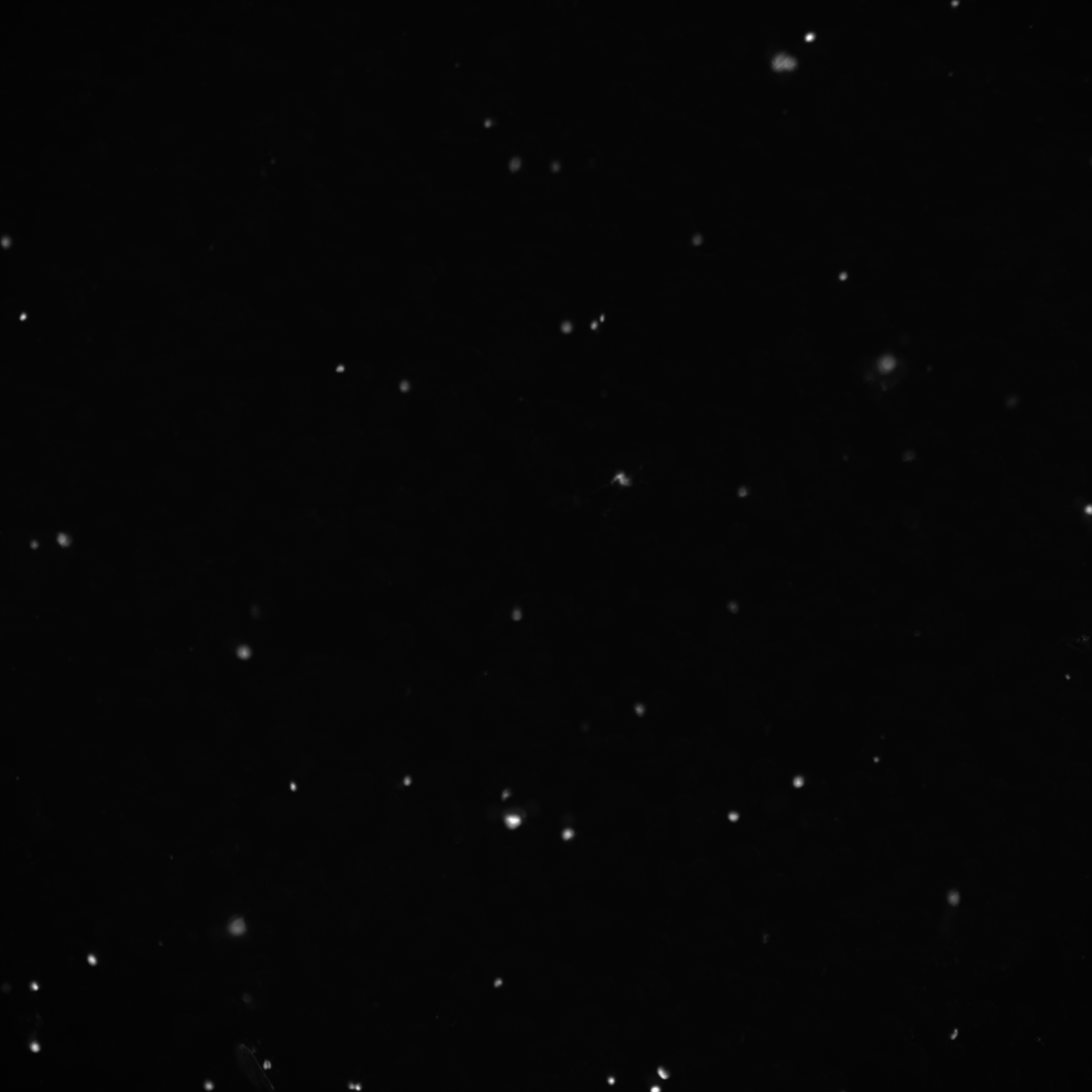

Supplement: Supplementary Software — Matlab code used for image analysis as well as LabVIEW code for microscope control [file ncomms11636-s3.zip › code/Viability/images/TIME02H_FOV1_PI.tif]

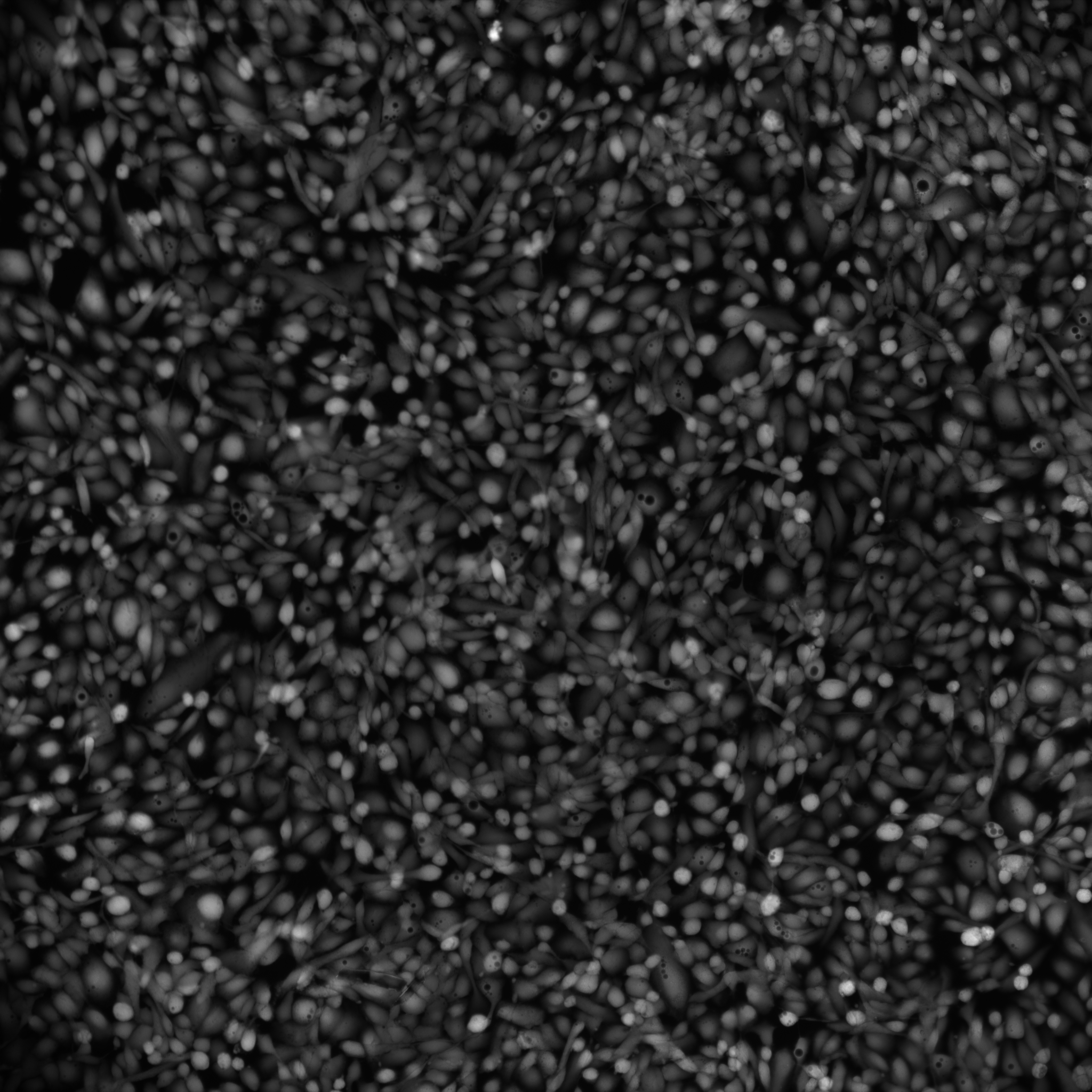

Supplement: Supplementary Software — Matlab code used for image analysis as well as LabVIEW code for microscope control [file ncomms11636-s3.zip › code/Viability/images/TIME24H_FOV0_CAL.tif]

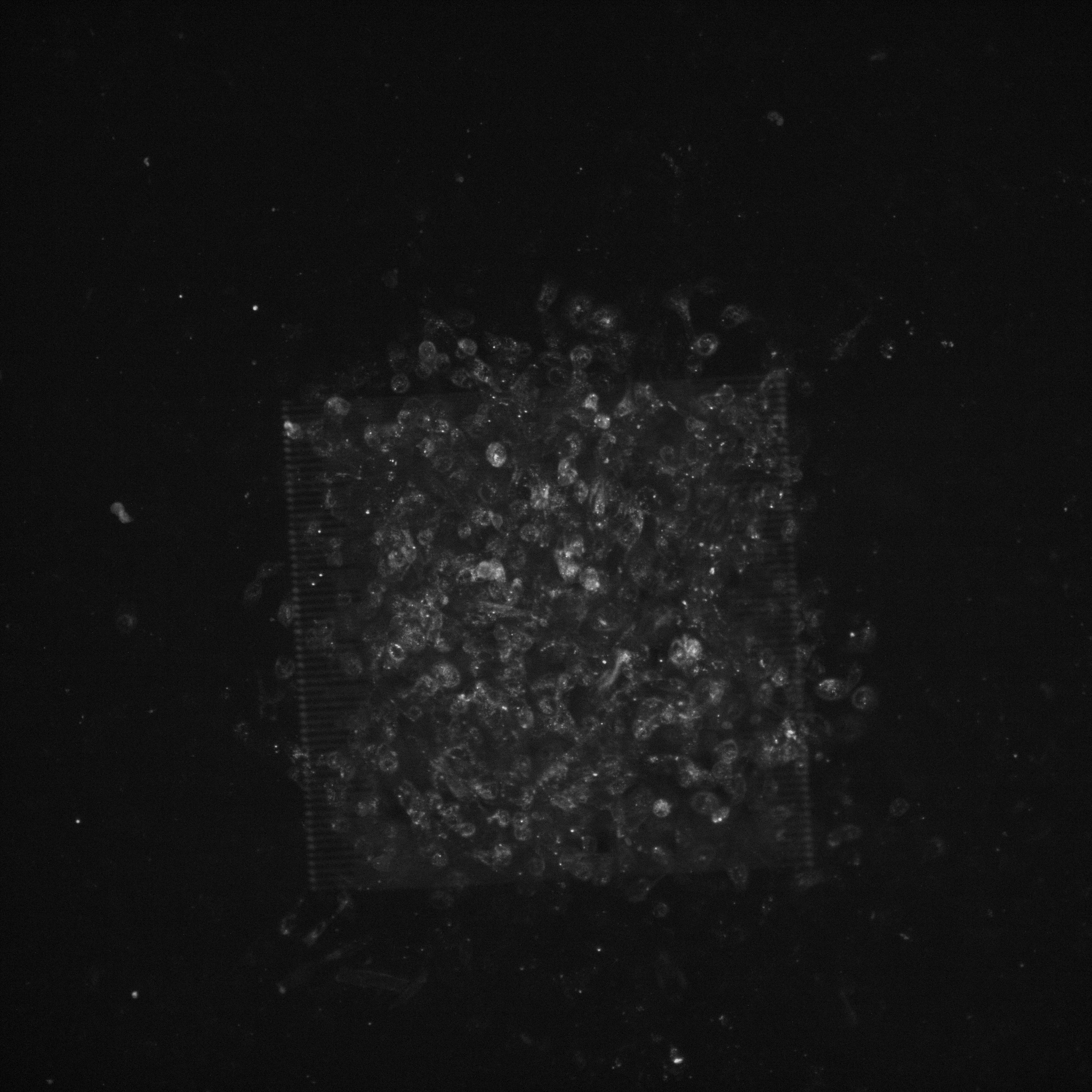

Supplement: Supplementary Software — Matlab code used for image analysis as well as LabVIEW code for microscope control [file ncomms11636-s3.zip › code/Viability/images/TIME24H_FOV0_CY5.tif]

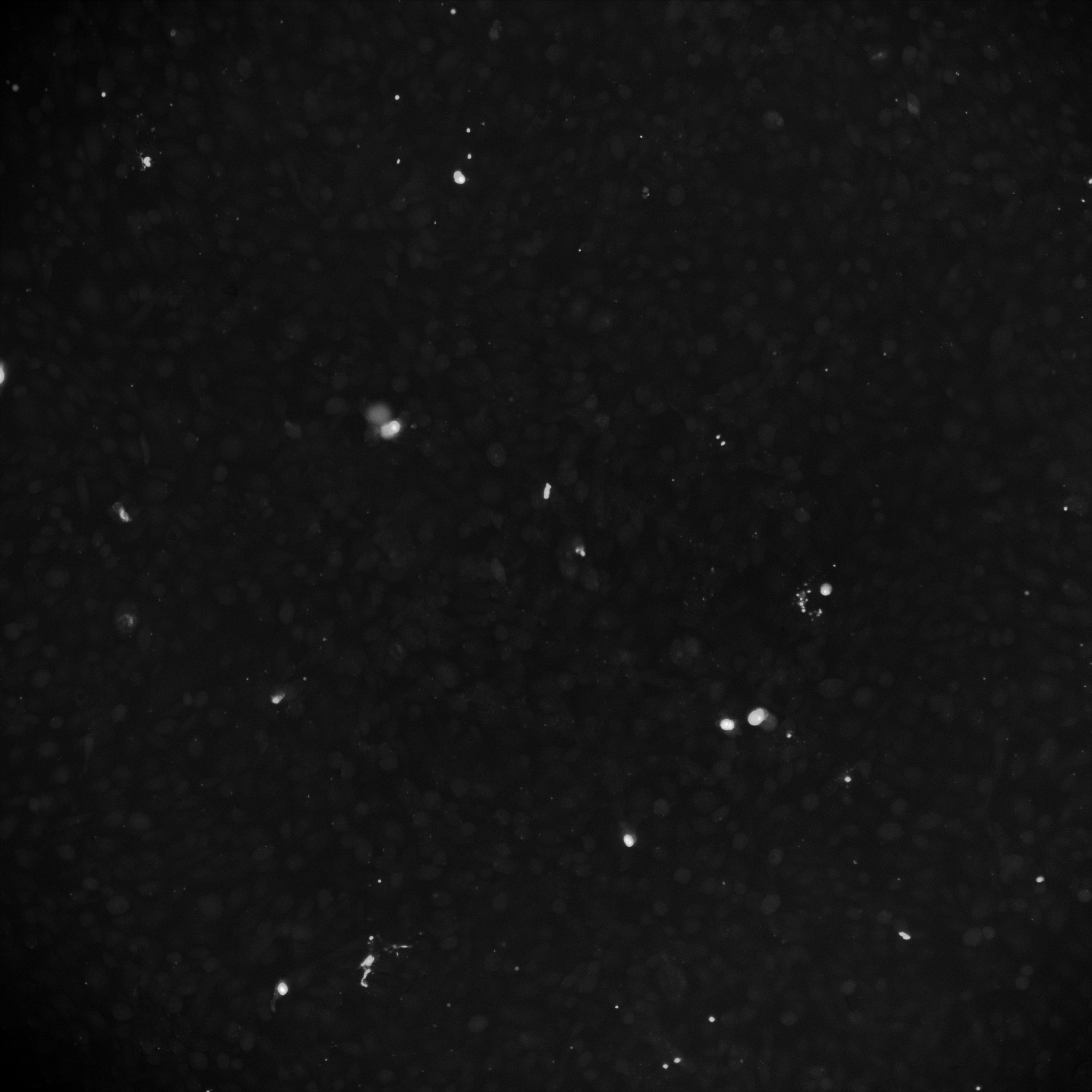

Supplement: Supplementary Software — Matlab code used for image analysis as well as LabVIEW code for microscope control [file ncomms11636-s3.zip › code/Viability/images/TIME24H_FOV0_PI.tif]

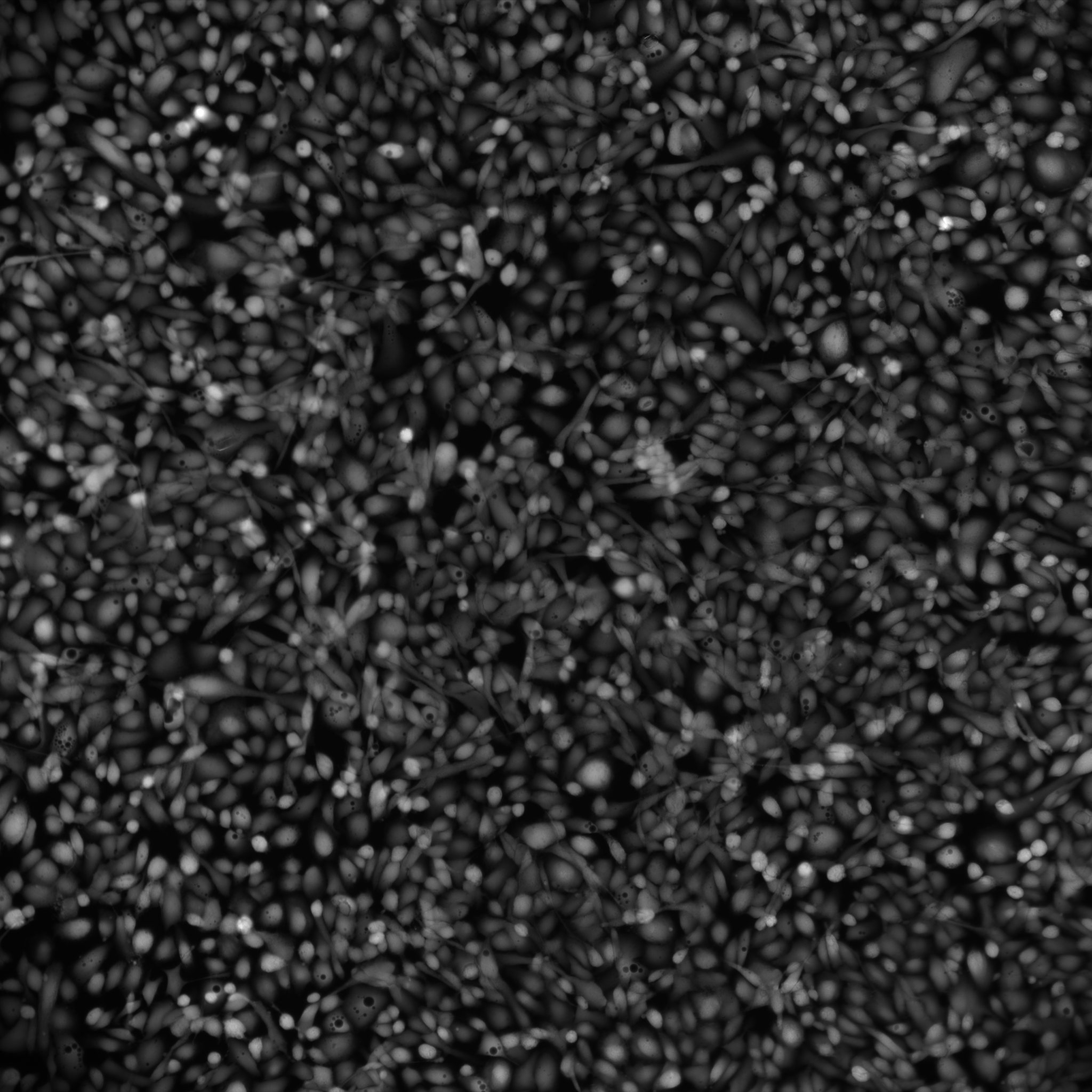

Supplement: Supplementary Software — Matlab code used for image analysis as well as LabVIEW code for microscope control [file ncomms11636-s3.zip › code/Viability/images/TIME24H_FOV1_CAL.tif]

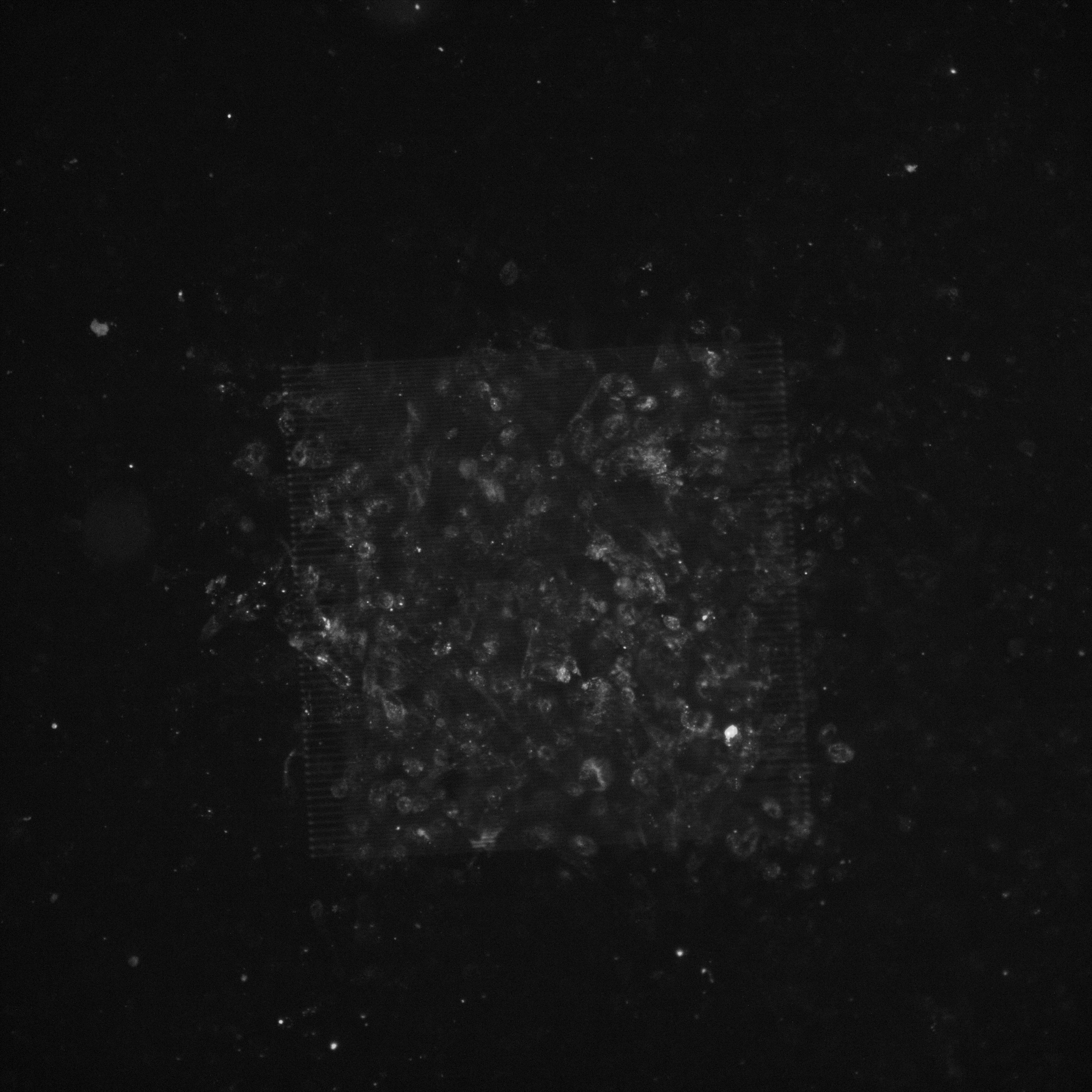

Supplement: Supplementary Software — Matlab code used for image analysis as well as LabVIEW code for microscope control [file ncomms11636-s3.zip › code/Viability/images/TIME24H_FOV1_CY5.tif]

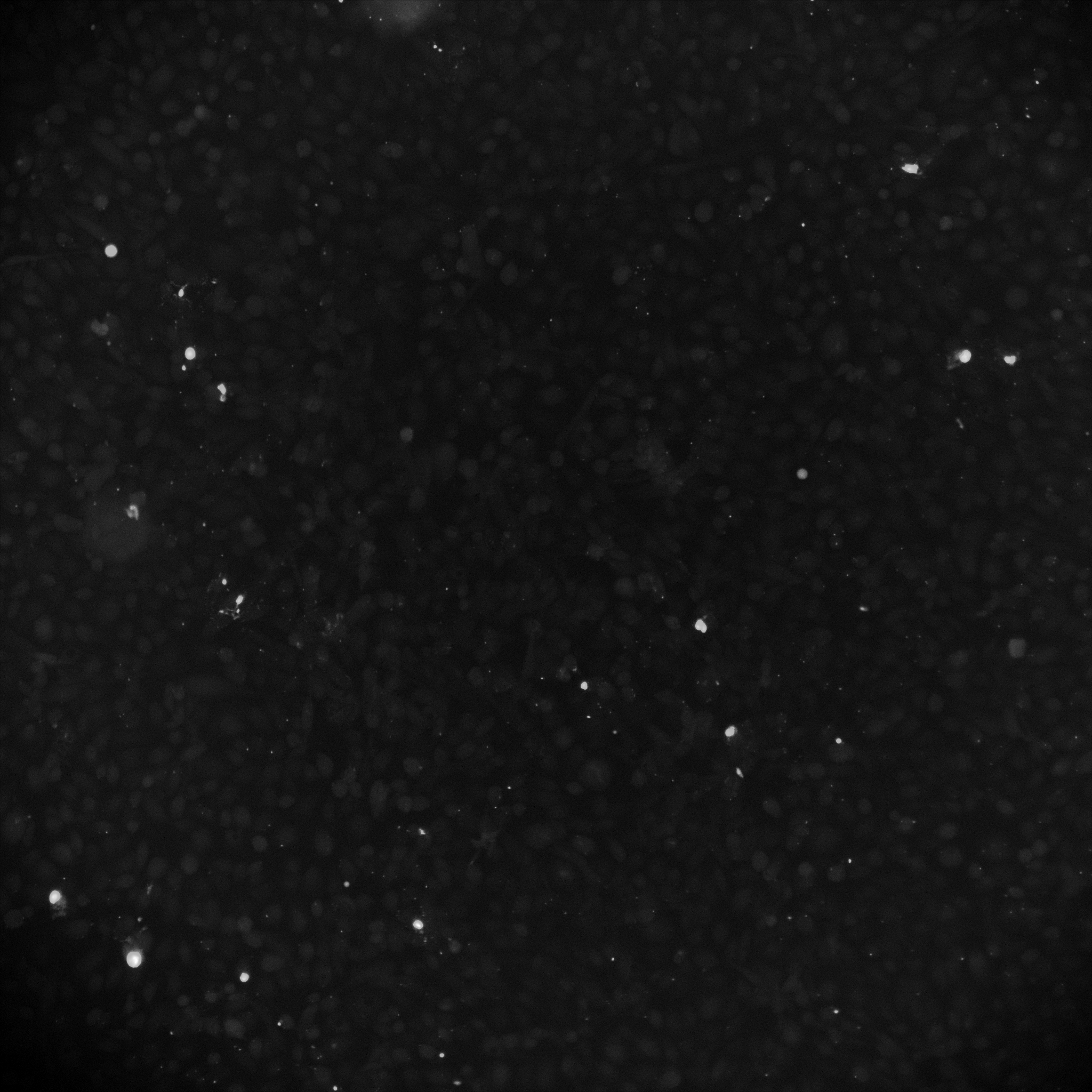

Supplement: Supplementary Software — Matlab code used for image analysis as well as LabVIEW code for microscope control [file ncomms11636-s3.zip › code/Viability/images/TIME24H_FOV1_PI.tif]

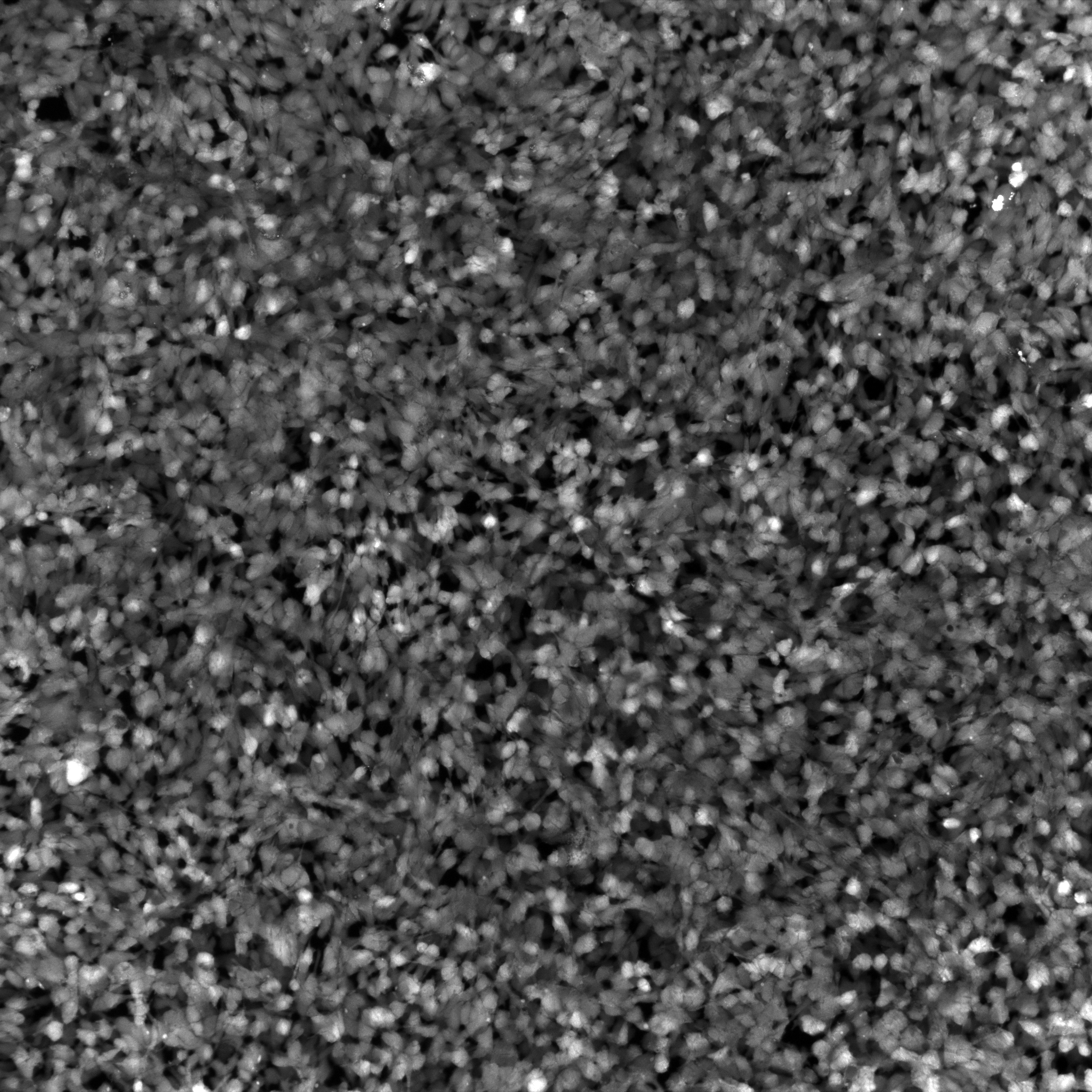

Supplement: Supplementary Software — Matlab code used for image analysis as well as LabVIEW code for microscope control [file ncomms11636-s3.zip › code/Viability/images/TIME48H_FOV0_CAL.tif]

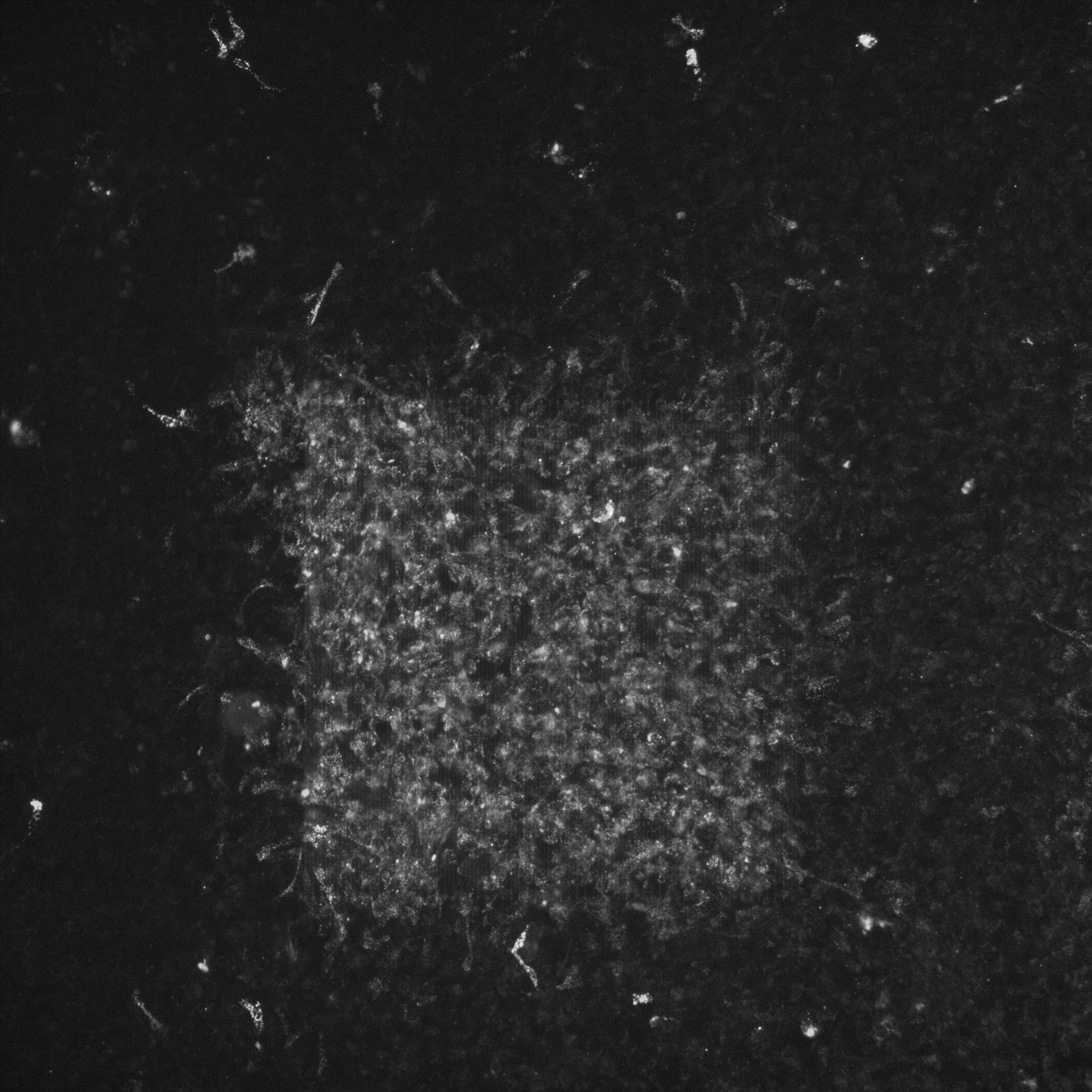

Supplement: Supplementary Software — Matlab code used for image analysis as well as LabVIEW code for microscope control [file ncomms11636-s3.zip › code/Viability/images/TIME48H_FOV0_CY5.tif]

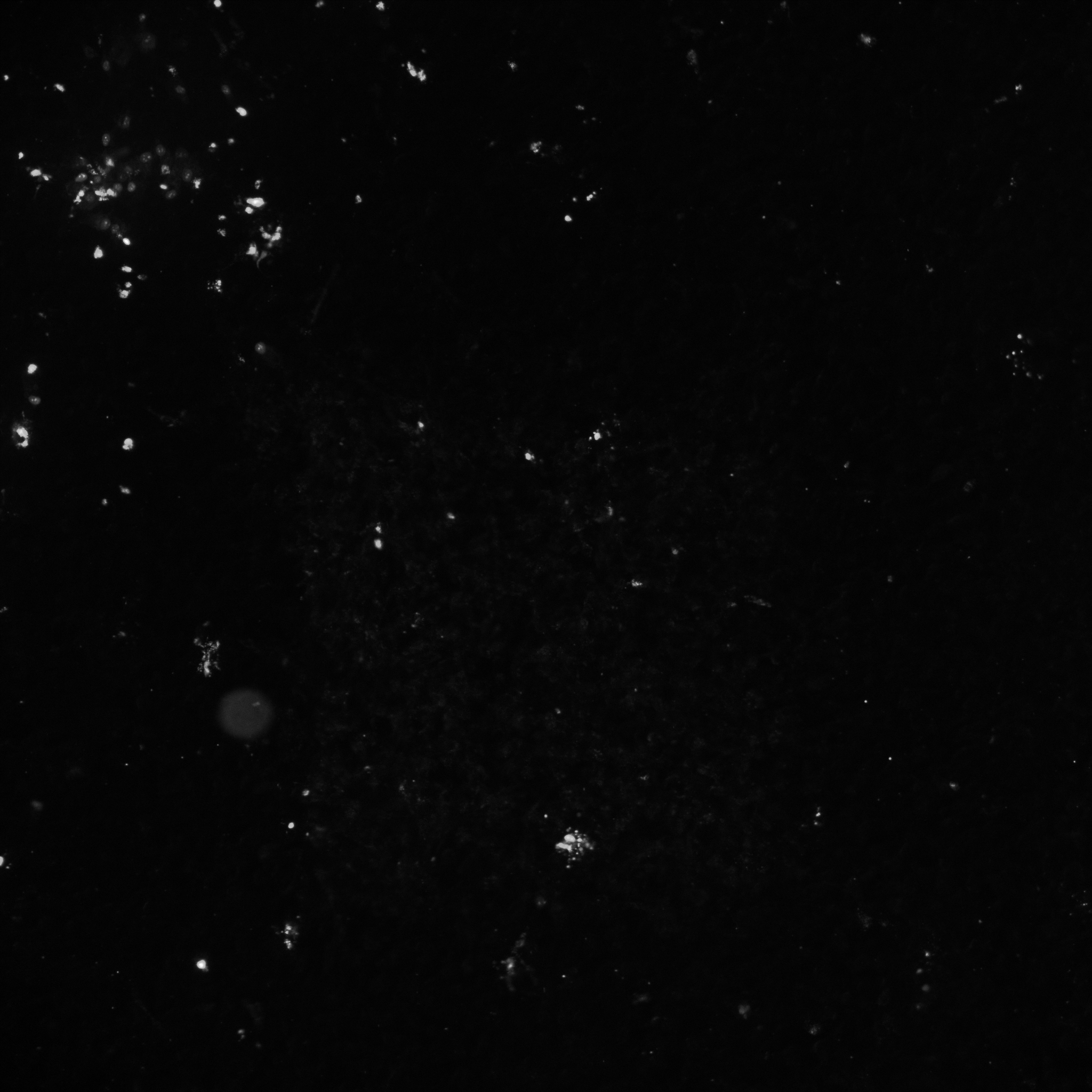

Supplement: Supplementary Software — Matlab code used for image analysis as well as LabVIEW code for microscope control [file ncomms11636-s3.zip › code/Viability/images/TIME48H_FOV0_PI.tif]

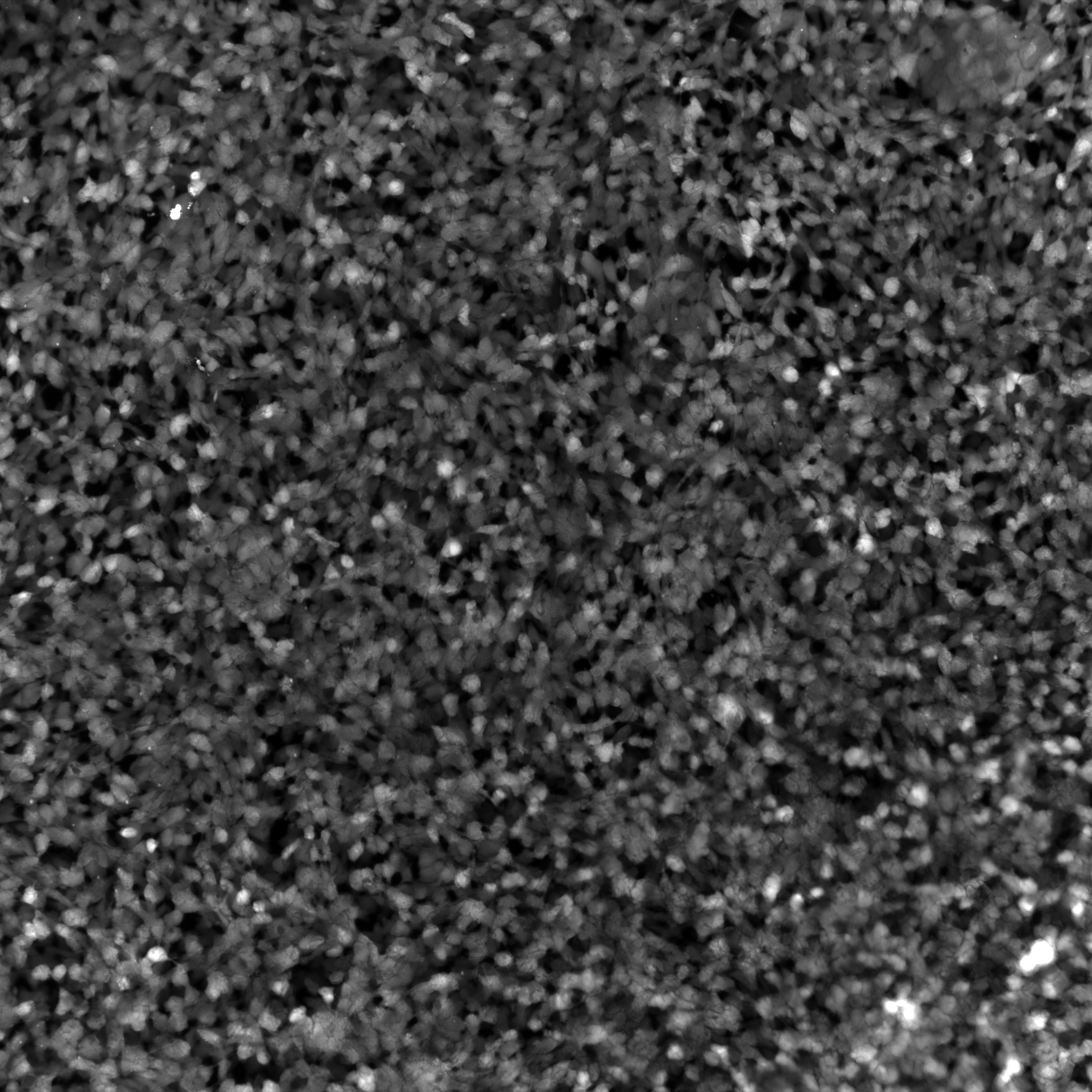

Supplement: Supplementary Software — Matlab code used for image analysis as well as LabVIEW code for microscope control [file ncomms11636-s3.zip › code/Viability/images/TIME48H_FOV1_CAL.tif]

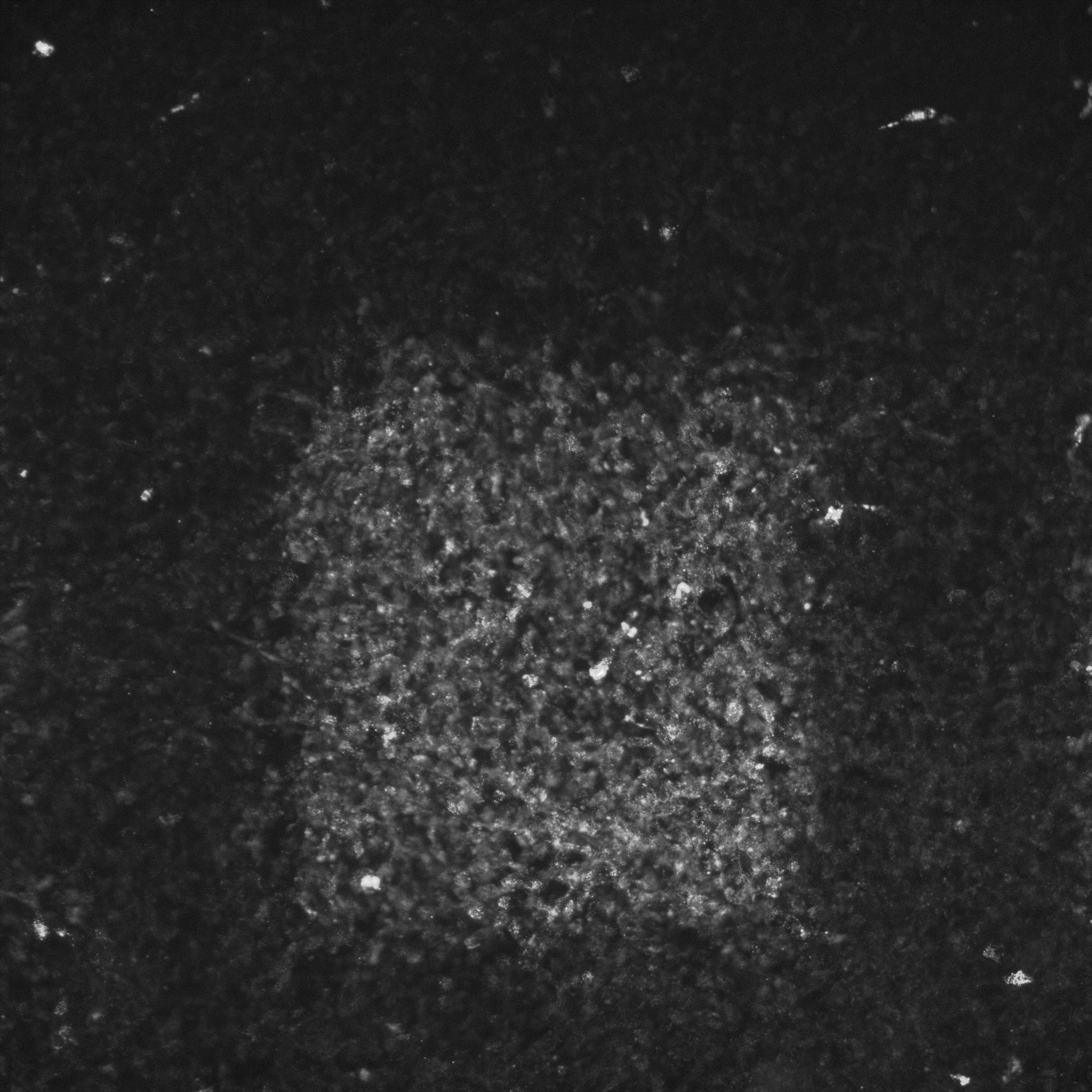

Supplement: Supplementary Software — Matlab code used for image analysis as well as LabVIEW code for microscope control [file ncomms11636-s3.zip › code/Viability/images/TIME48H_FOV1_CY5.tif]

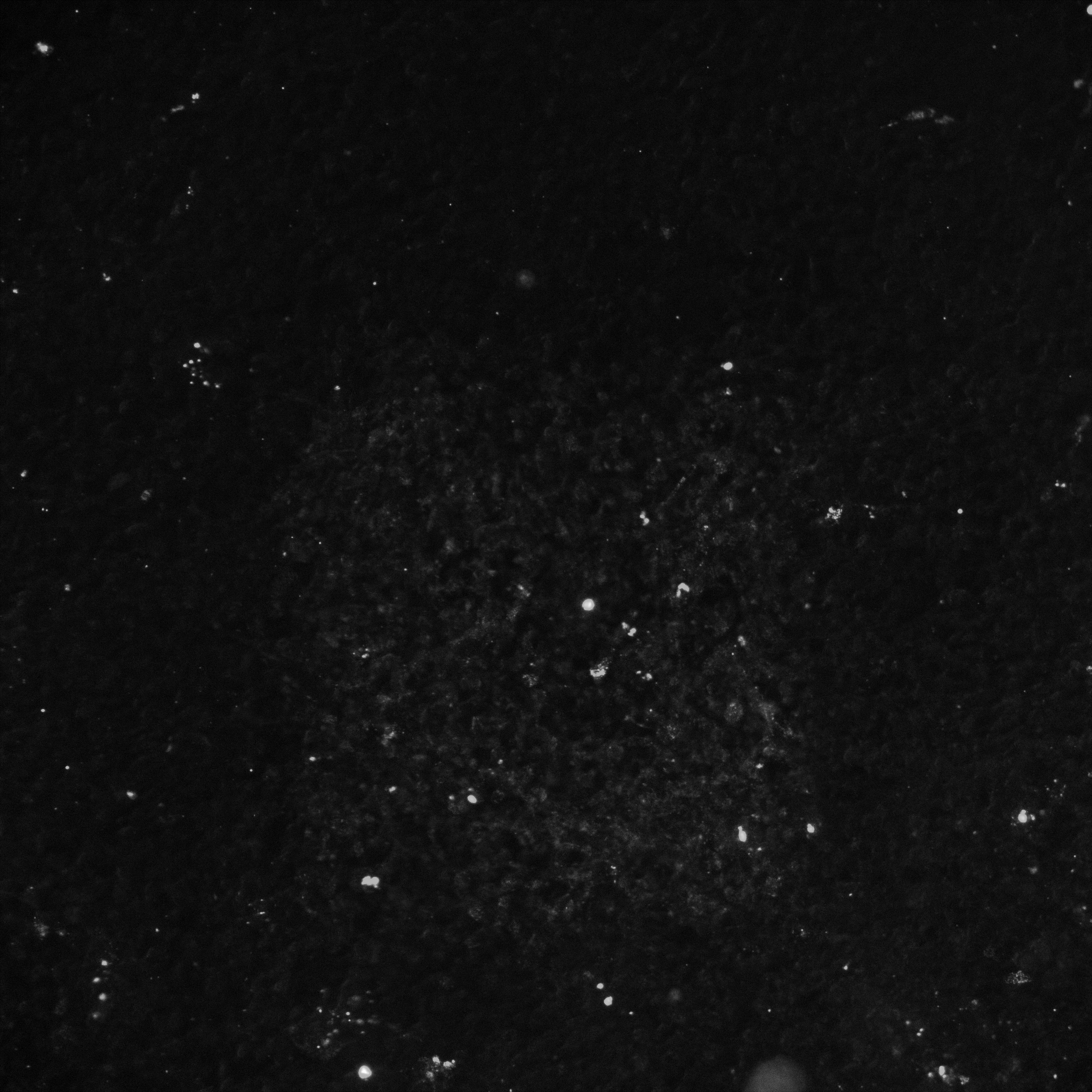

Supplement: Supplementary Software — Matlab code used for image analysis as well as LabVIEW code for microscope control [file ncomms11636-s3.zip › code/Viability/images/TIME48H_FOV1_PI.tif]

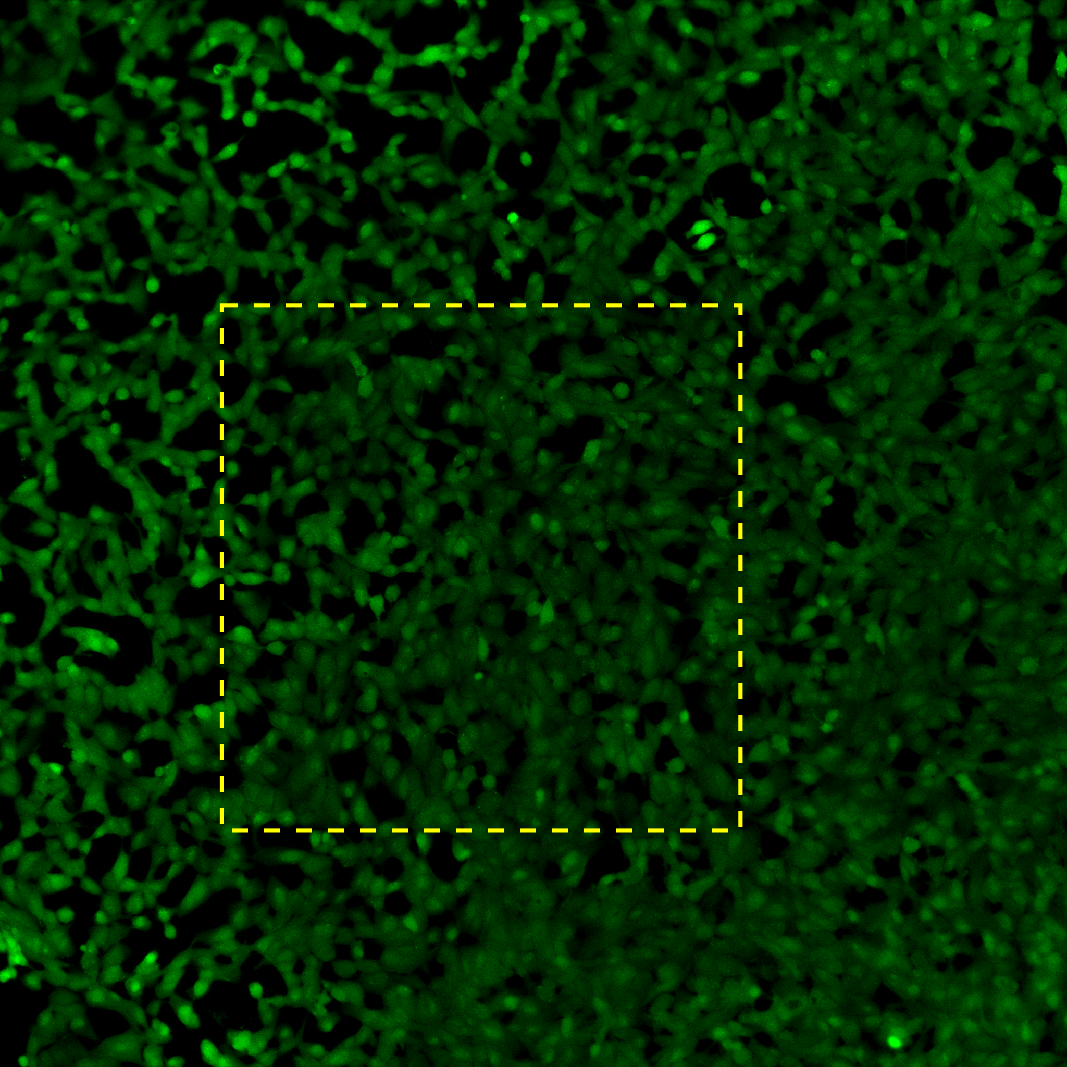

Supplement: Supplementary Software — Matlab code used for image analysis as well as LabVIEW code for microscope control [file ncomms11636-s3.zip › code/Viability/results/TIME00H_FOV0_CAL.png]

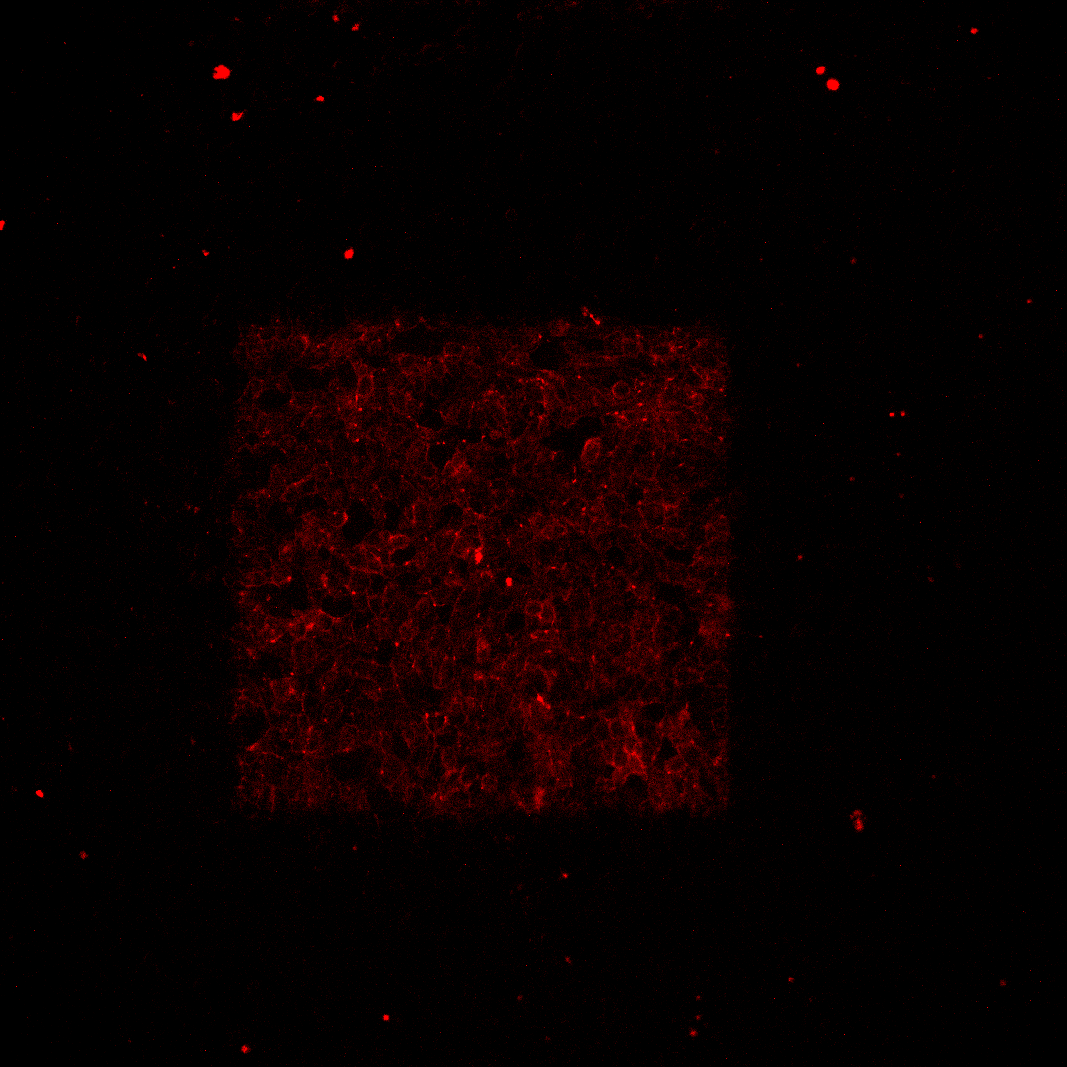

Supplement: Supplementary Software — Matlab code used for image analysis as well as LabVIEW code for microscope control [file ncomms11636-s3.zip › code/Viability/results/TIME00H_FOV0_CY5.png]

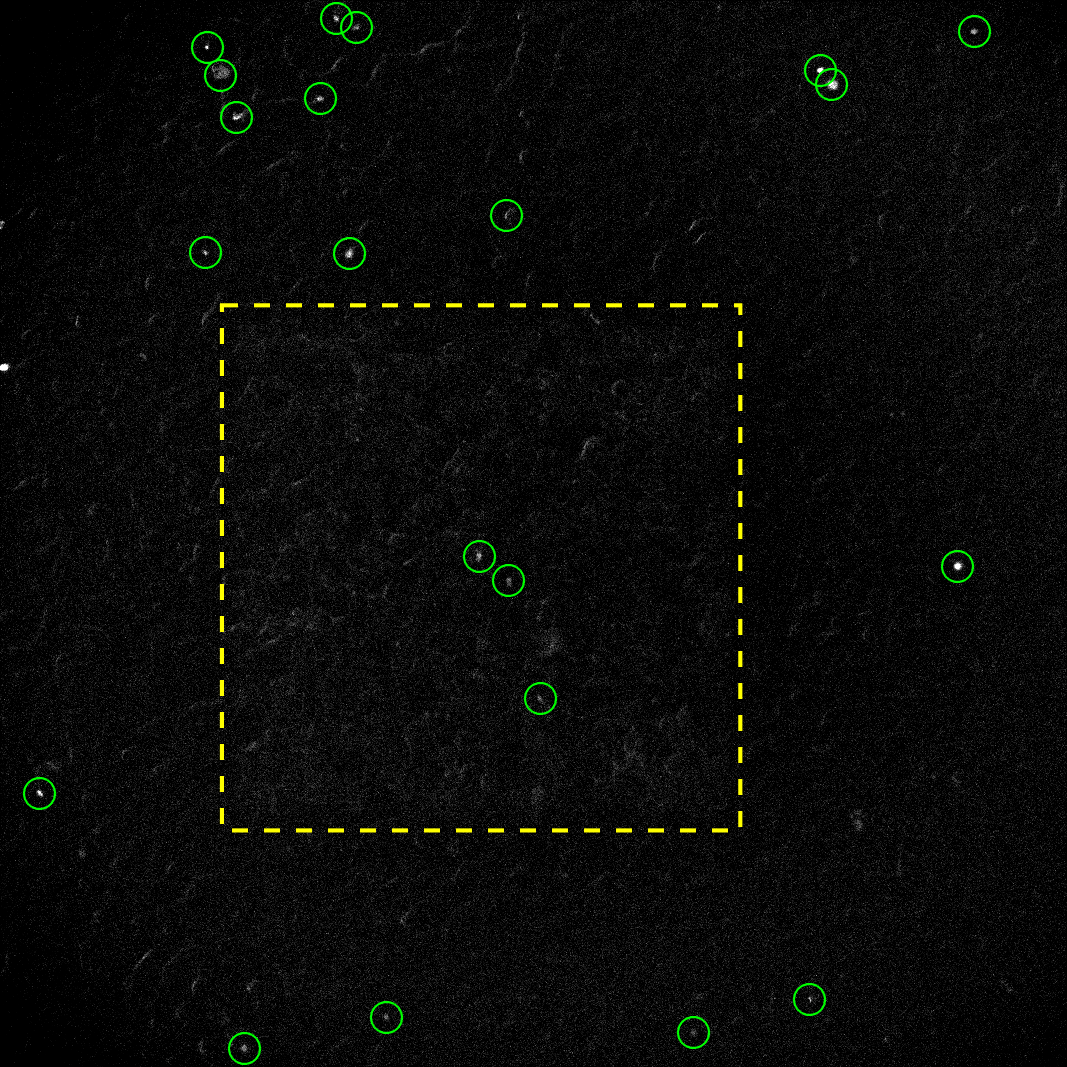

Supplement: Supplementary Software — Matlab code used for image analysis as well as LabVIEW code for microscope control [file ncomms11636-s3.zip › code/Viability/results/TIME00H_FOV0_PI.png]

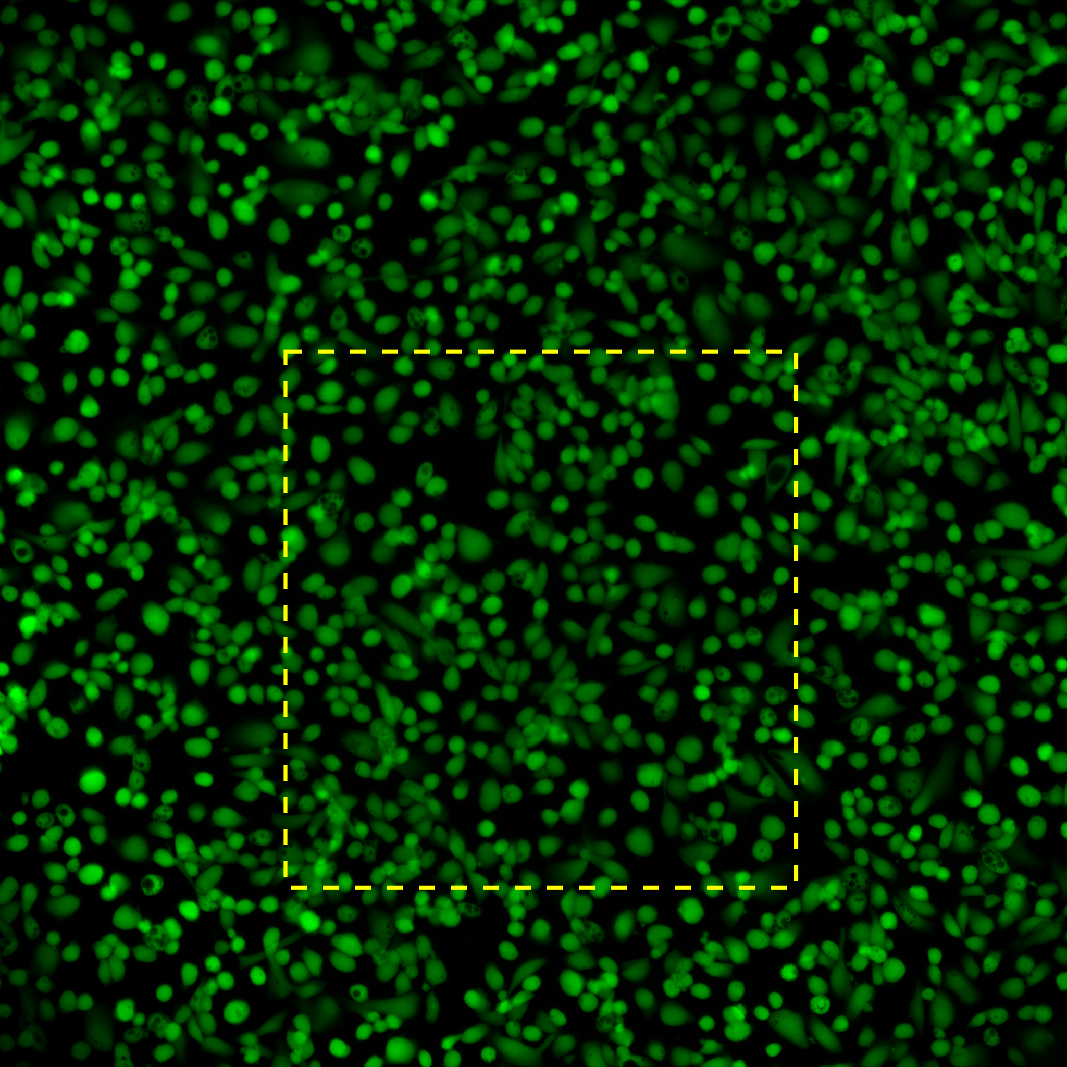

Supplement: Supplementary Software — Matlab code used for image analysis as well as LabVIEW code for microscope control [file ncomms11636-s3.zip › code/Viability/results/TIME02H_FOV0_CAL.png]

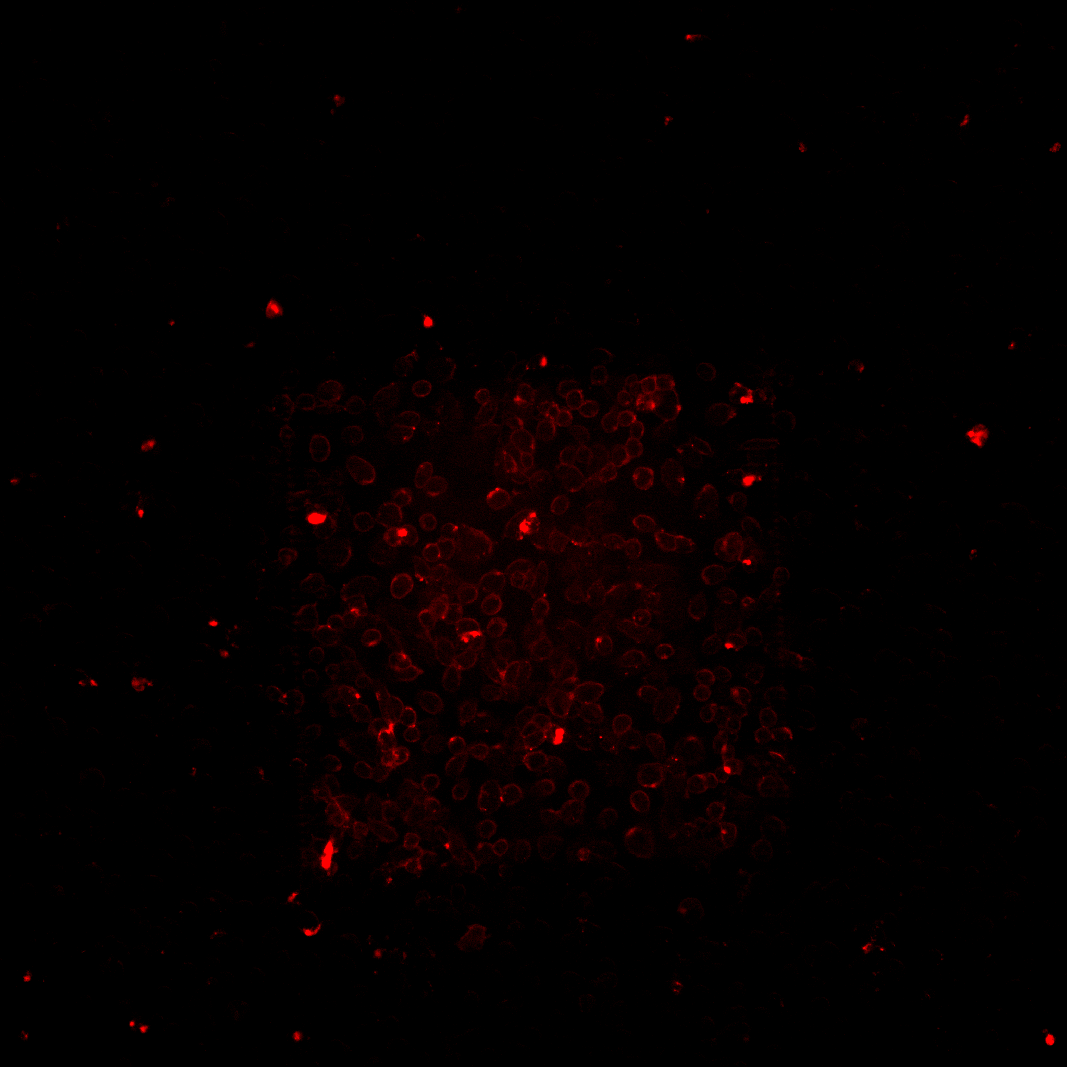

Supplement: Supplementary Software — Matlab code used for image analysis as well as LabVIEW code for microscope control [file ncomms11636-s3.zip › code/Viability/results/TIME02H_FOV0_CY5.png]

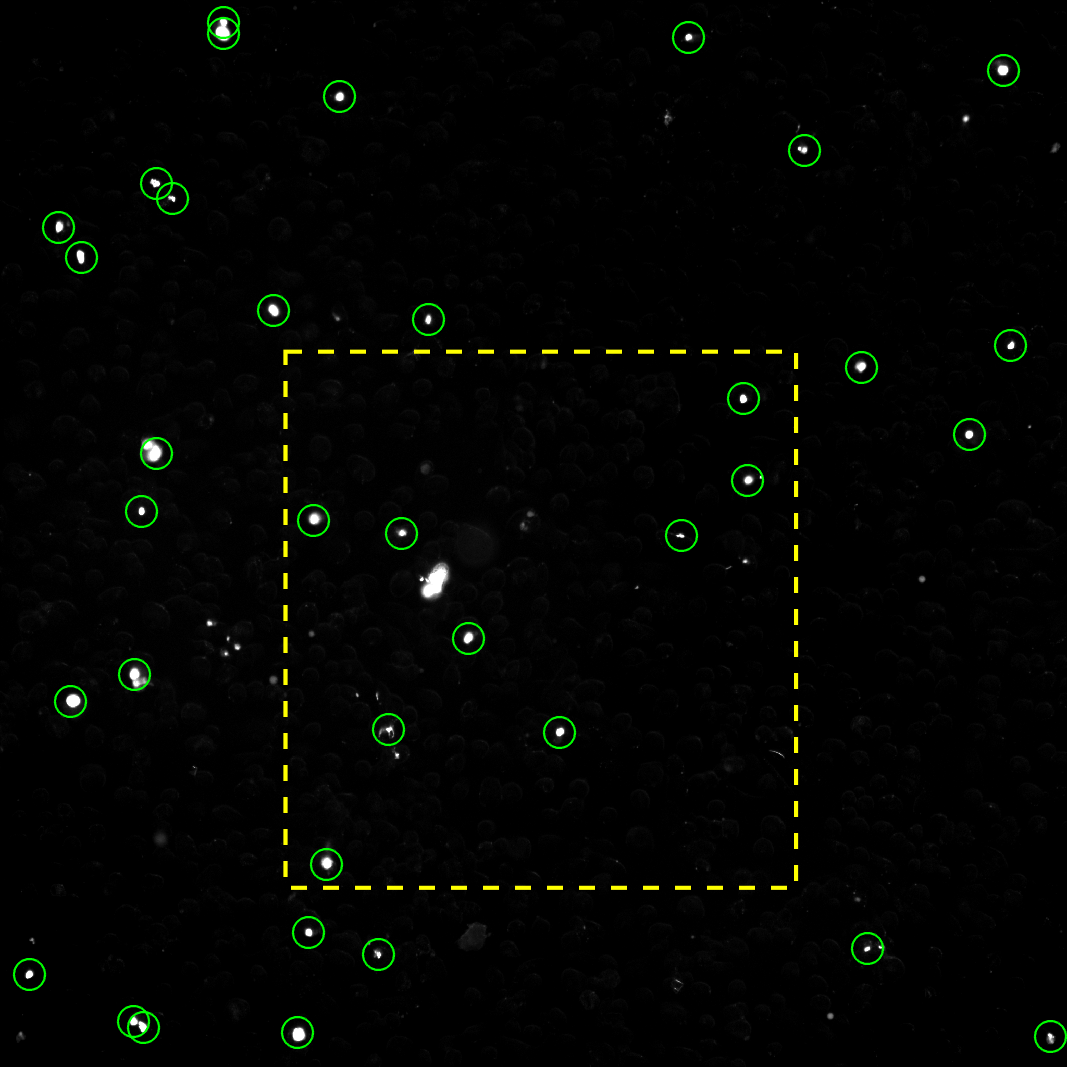

Supplement: Supplementary Software — Matlab code used for image analysis as well as LabVIEW code for microscope control [file ncomms11636-s3.zip › code/Viability/results/TIME02H_FOV0_PI.png]

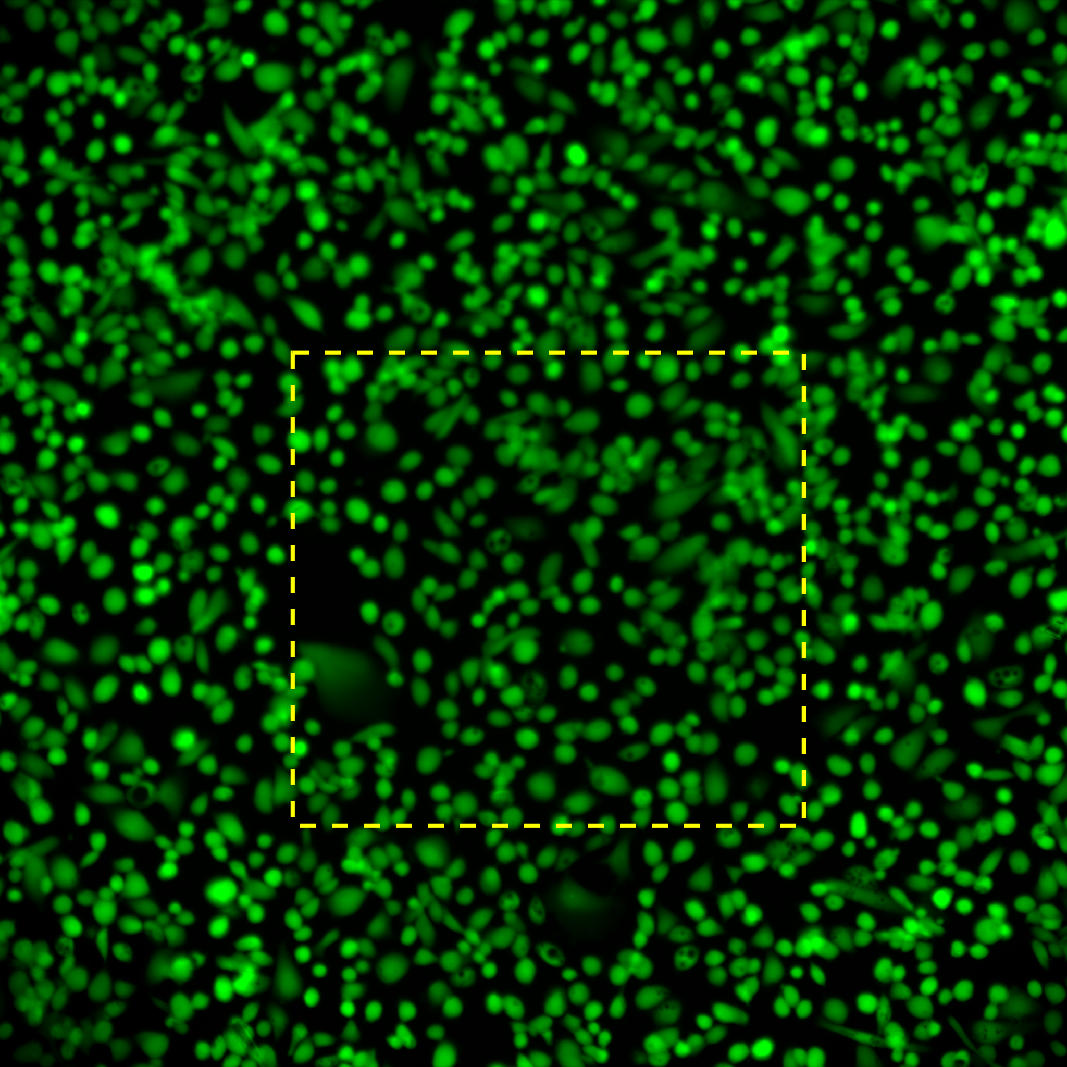

Supplement: Supplementary Software — Matlab code used for image analysis as well as LabVIEW code for microscope control [file ncomms11636-s3.zip › code/Viability/results/TIME02H_FOV1_CAL.png]

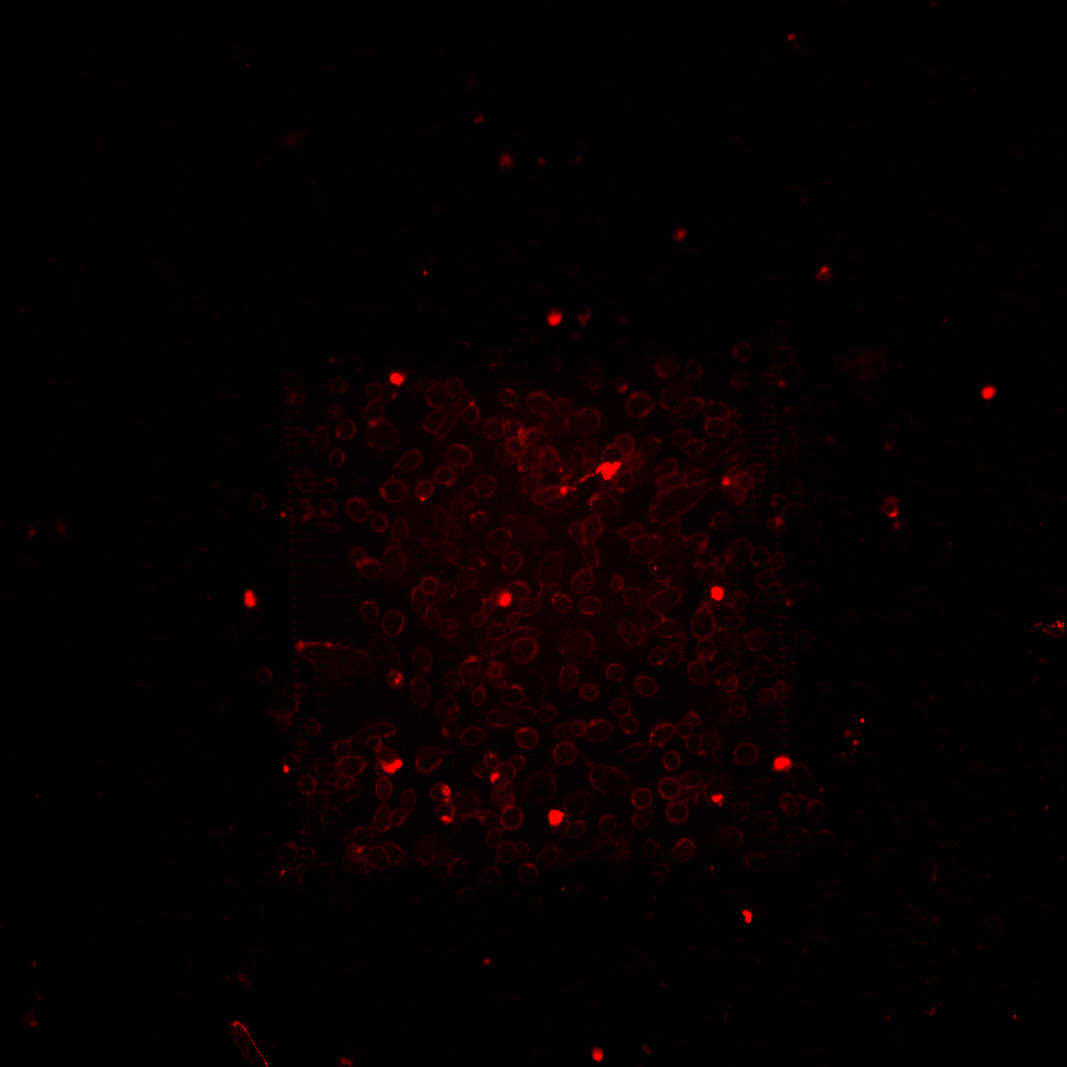

Supplement: Supplementary Software — Matlab code used for image analysis as well as LabVIEW code for microscope control [file ncomms11636-s3.zip › code/Viability/results/TIME02H_FOV1_CY5.png]

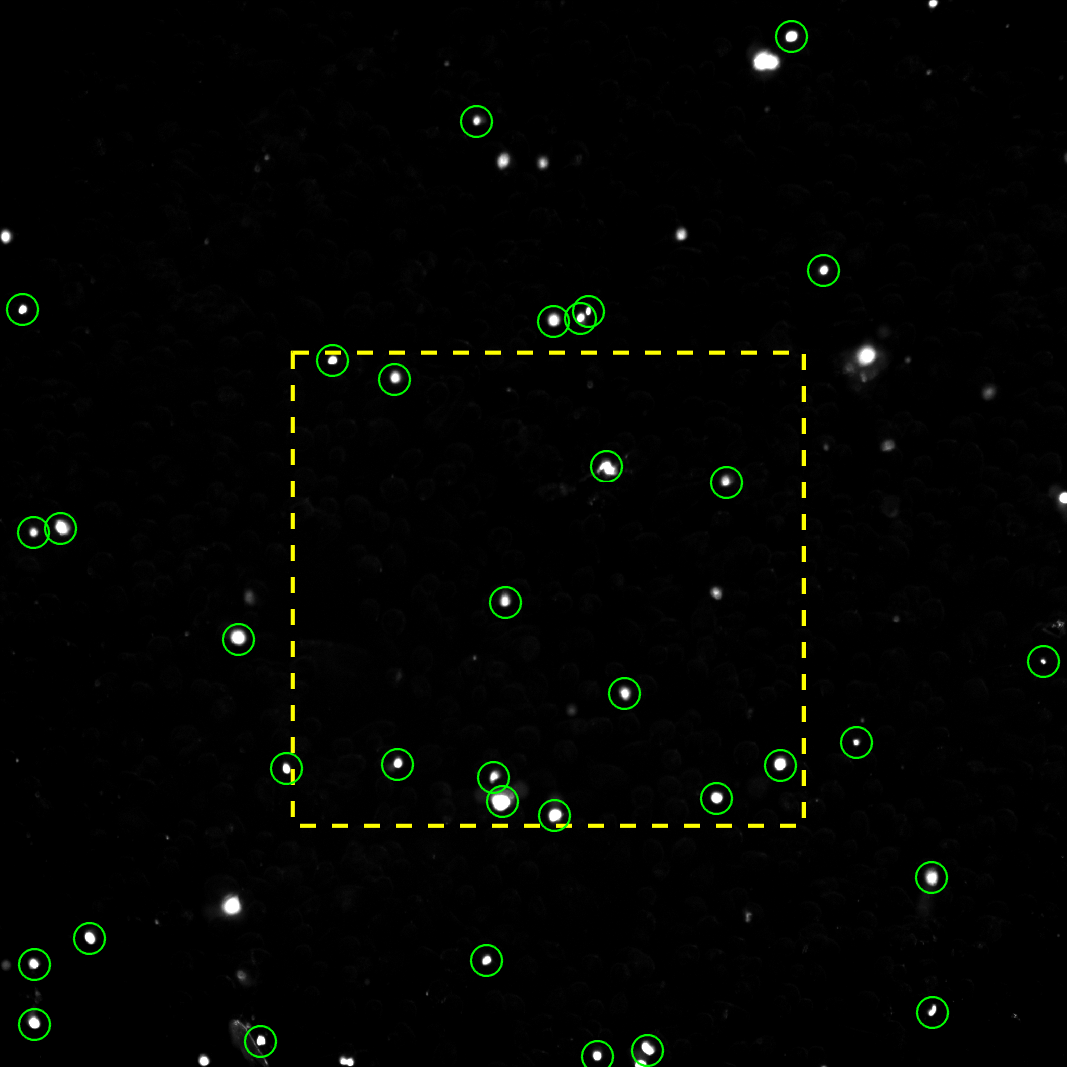

Supplement: Supplementary Software — Matlab code used for image analysis as well as LabVIEW code for microscope control [file ncomms11636-s3.zip › code/Viability/results/TIME02H_FOV1_PI.png]

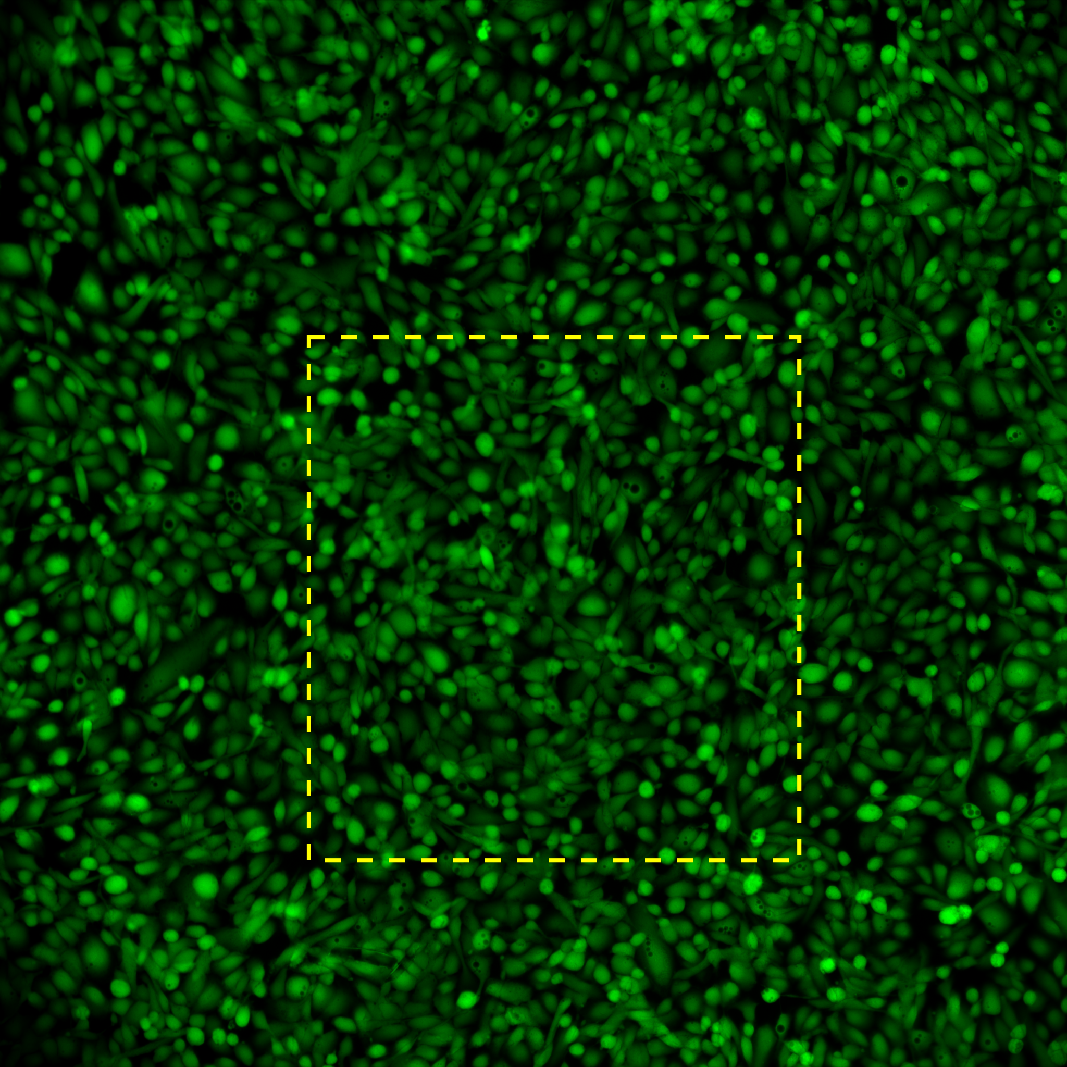

Supplement: Supplementary Software — Matlab code used for image analysis as well as LabVIEW code for microscope control [file ncomms11636-s3.zip › code/Viability/results/TIME24H_FOV0_CAL.png]

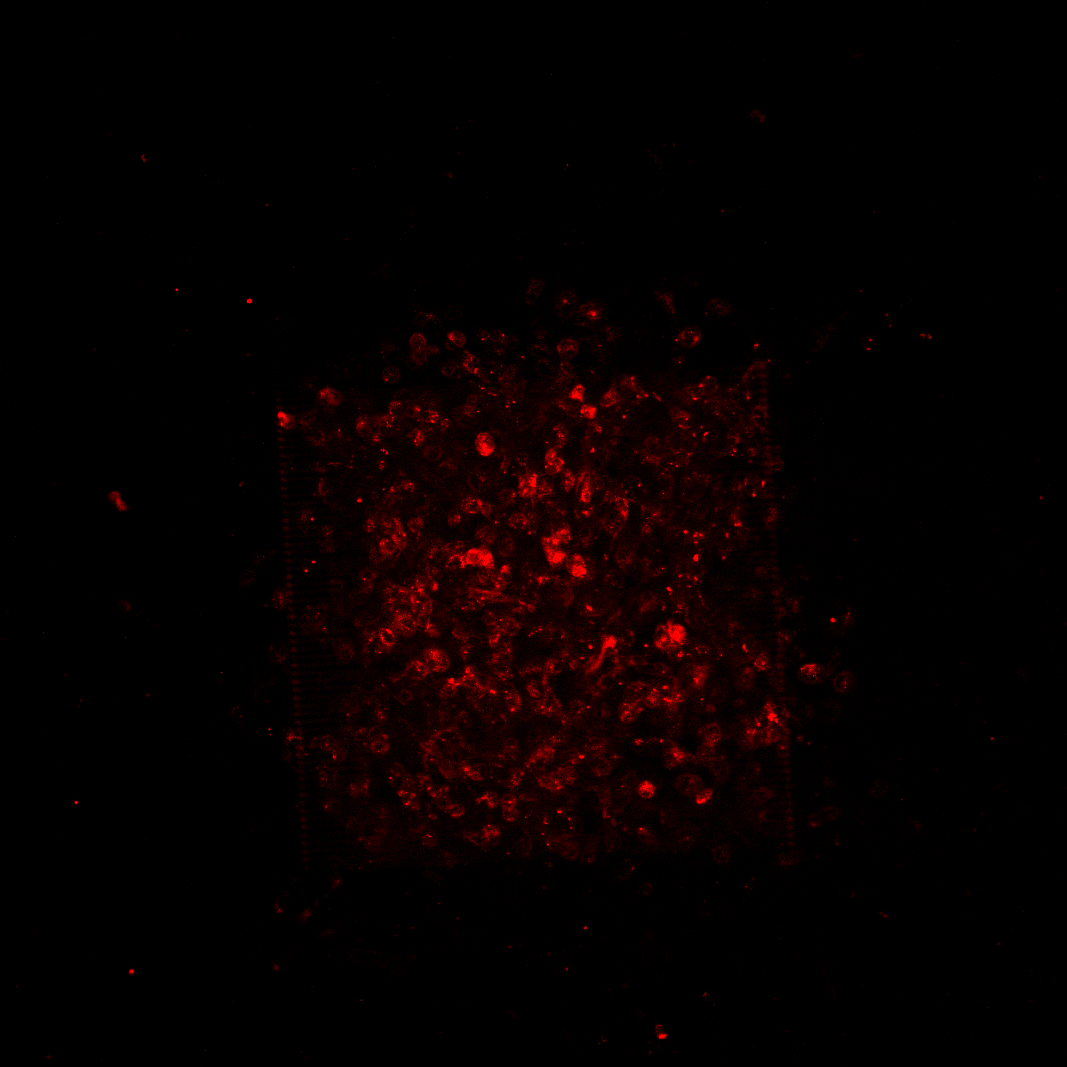

Supplement: Supplementary Software — Matlab code used for image analysis as well as LabVIEW code for microscope control [file ncomms11636-s3.zip › code/Viability/results/TIME24H_FOV0_CY5.png]

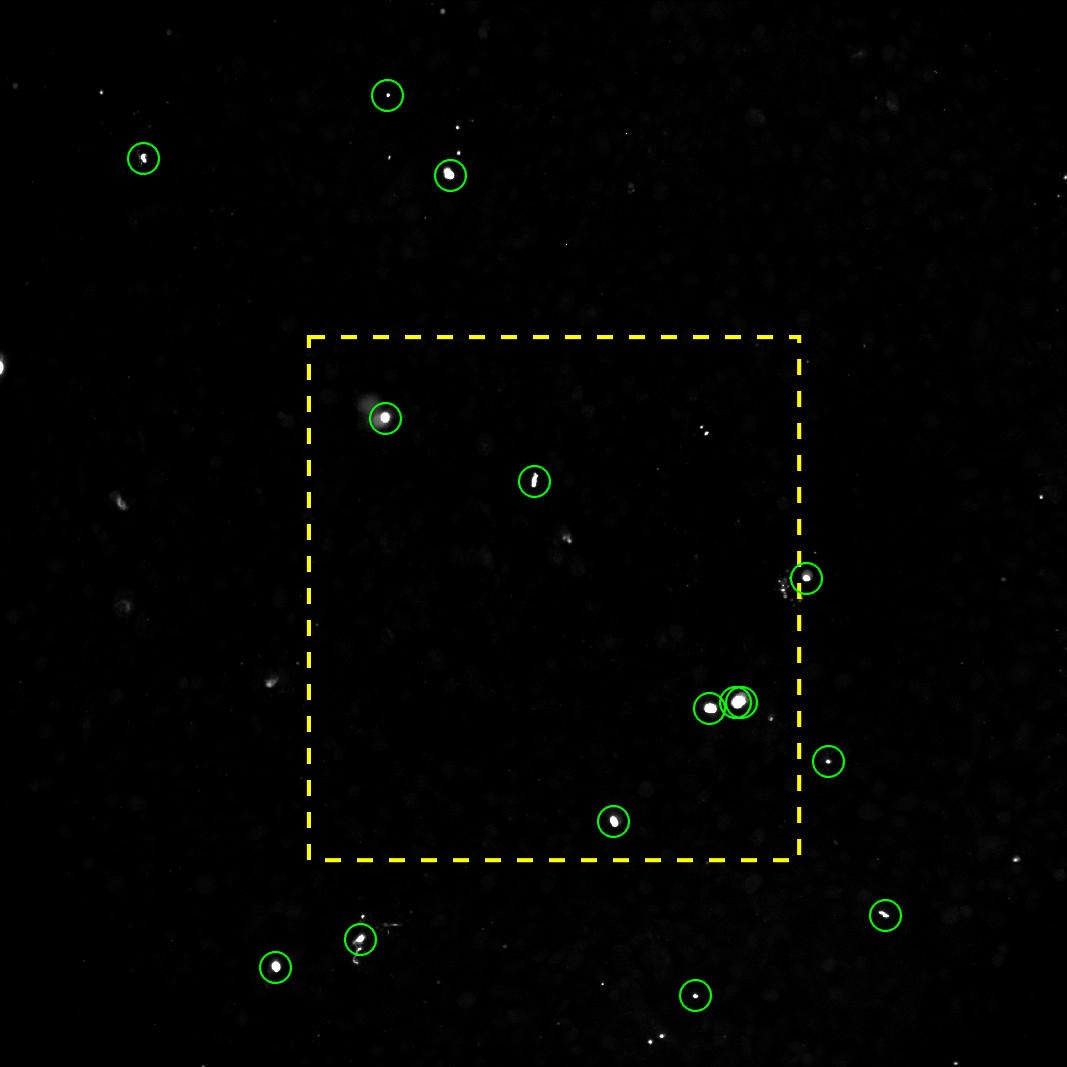

Supplement: Supplementary Software — Matlab code used for image analysis as well as LabVIEW code for microscope control [file ncomms11636-s3.zip › code/Viability/results/TIME24H_FOV0_PI.png]

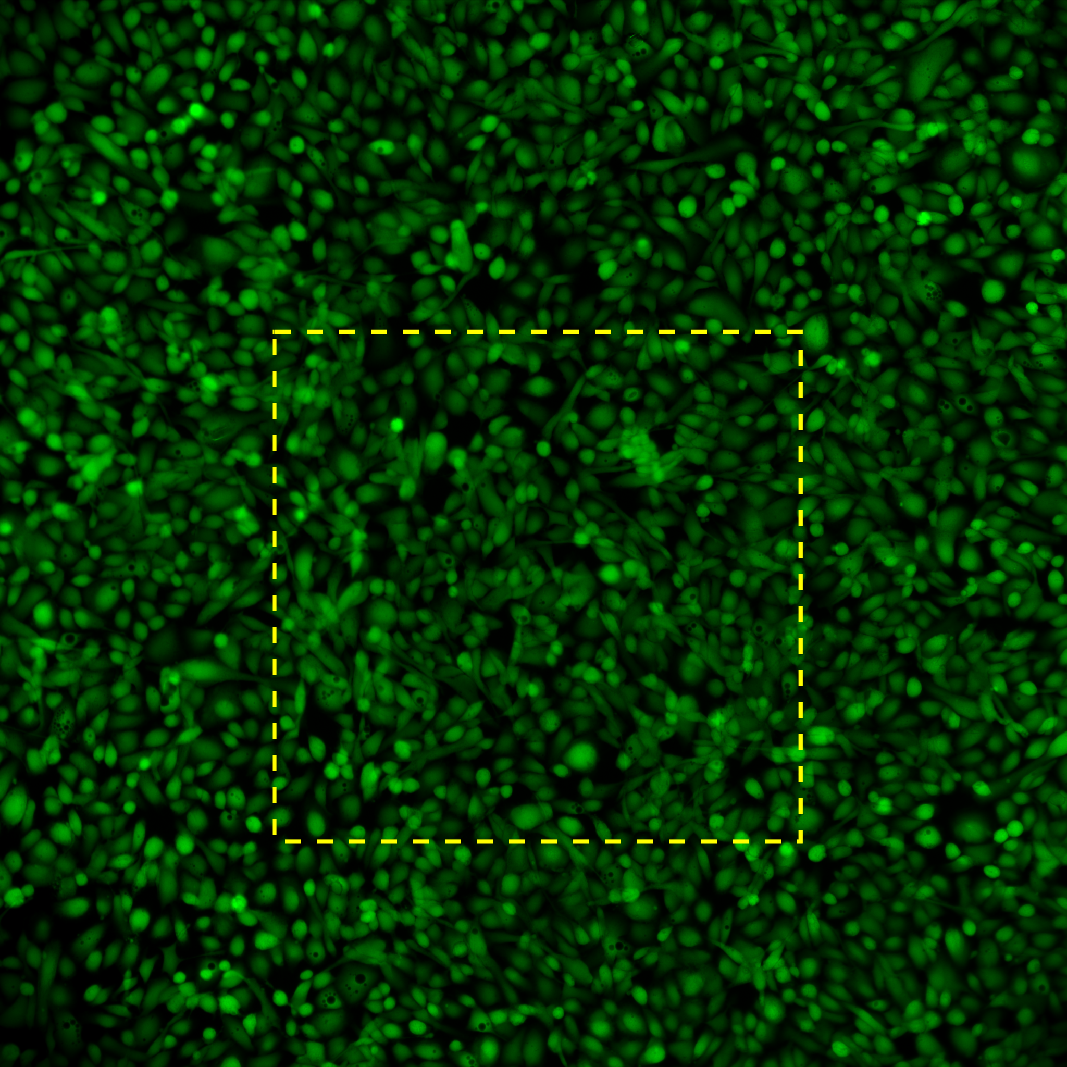

Supplement: Supplementary Software — Matlab code used for image analysis as well as LabVIEW code for microscope control [file ncomms11636-s3.zip › code/Viability/results/TIME24H_FOV1_CAL.png]

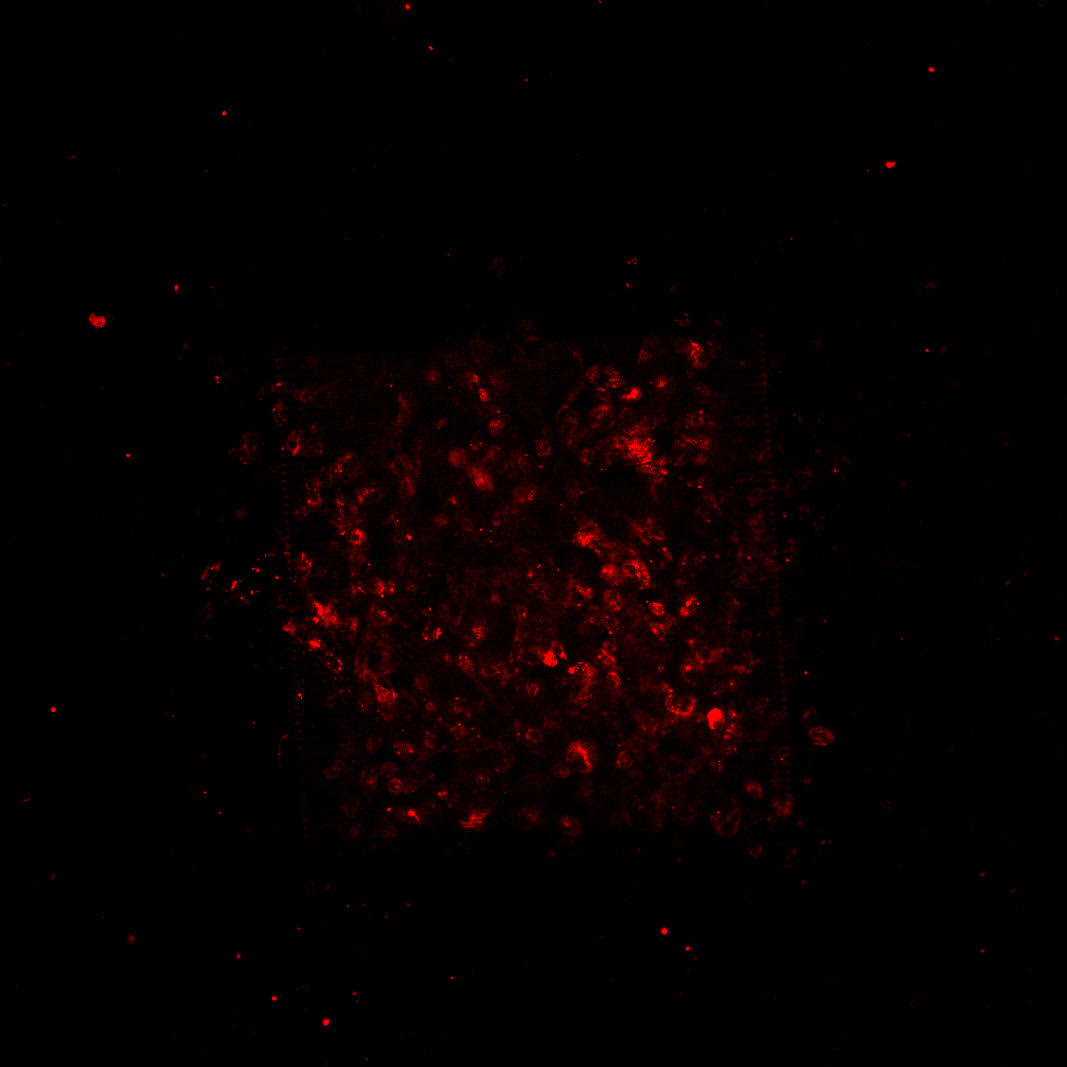

Supplement: Supplementary Software — Matlab code used for image analysis as well as LabVIEW code for microscope control [file ncomms11636-s3.zip › code/Viability/results/TIME24H_FOV1_CY5.png]

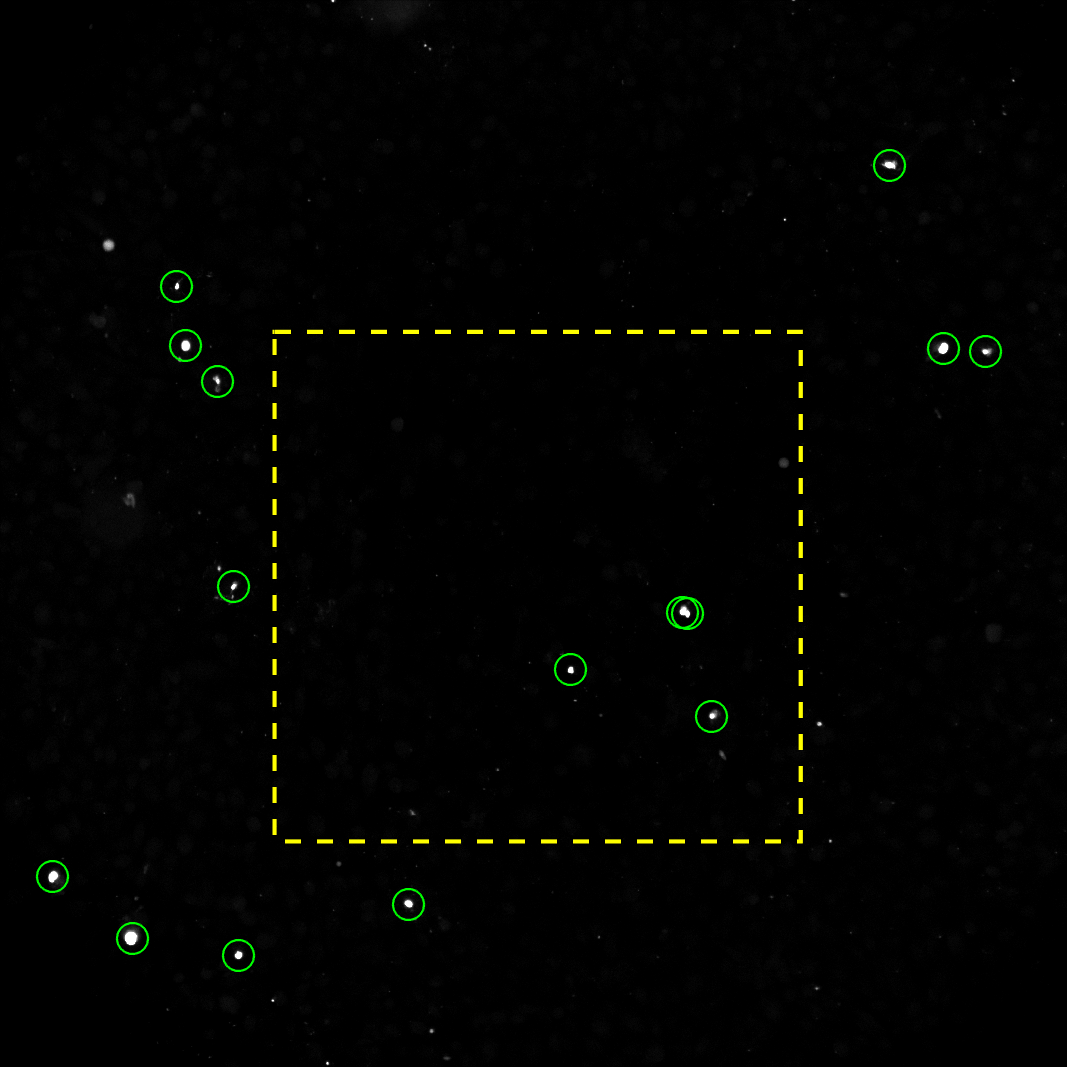

Supplement: Supplementary Software — Matlab code used for image analysis as well as LabVIEW code for microscope control [file ncomms11636-s3.zip › code/Viability/results/TIME24H_FOV1_PI.png]

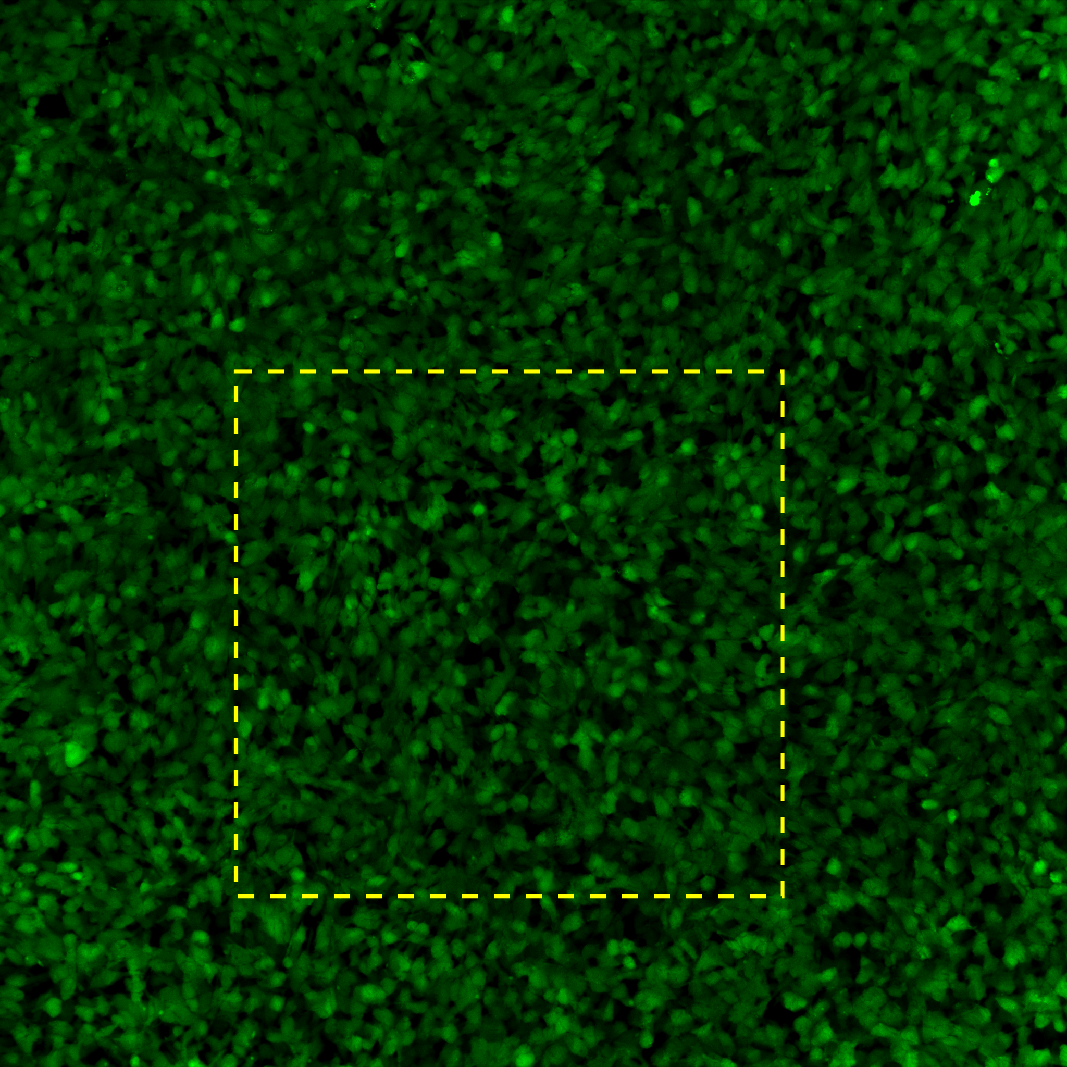

Supplement: Supplementary Software — Matlab code used for image analysis as well as LabVIEW code for microscope control [file ncomms11636-s3.zip › code/Viability/results/TIME48H_FOV0_CAL.png]

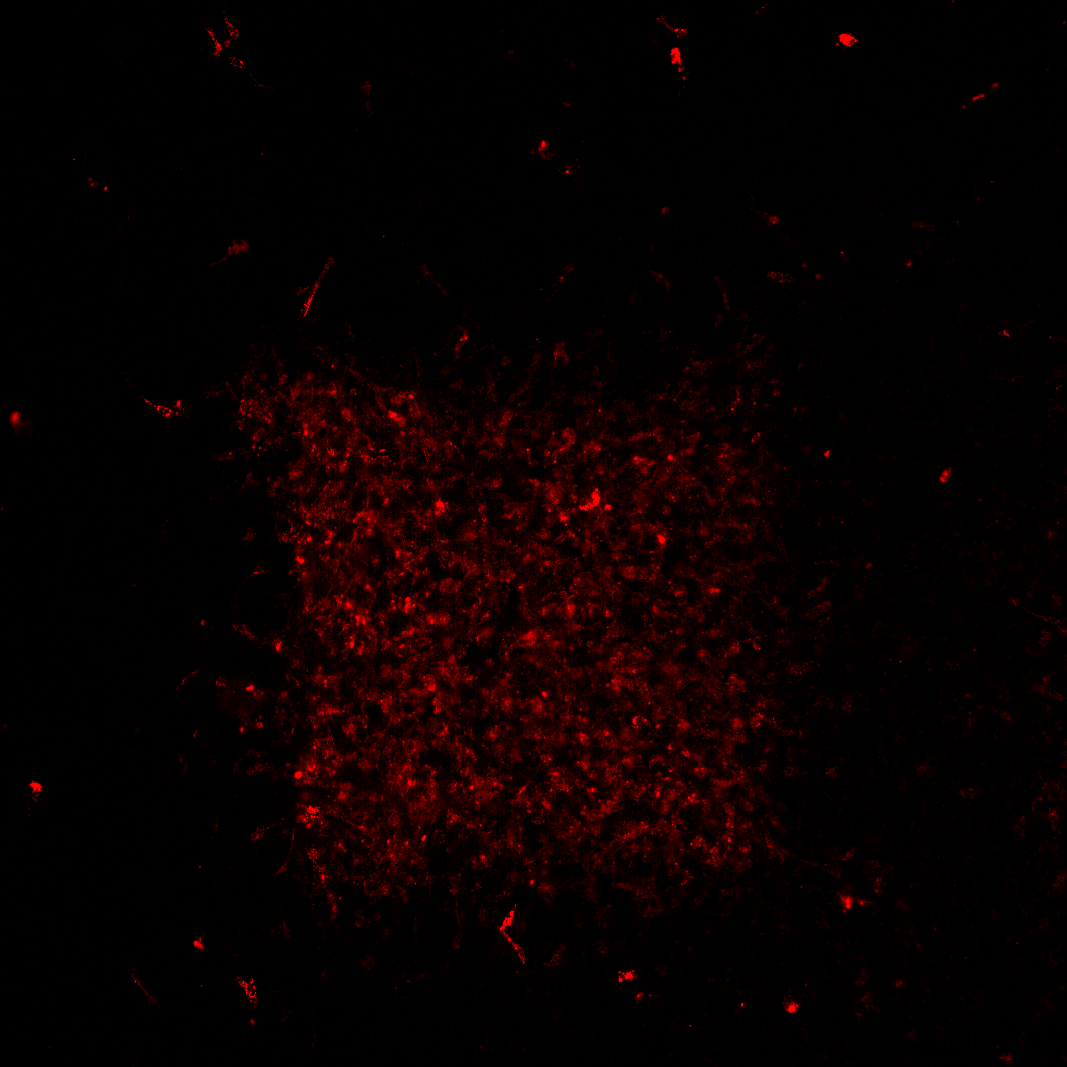

Supplement: Supplementary Software — Matlab code used for image analysis as well as LabVIEW code for microscope control [file ncomms11636-s3.zip › code/Viability/results/TIME48H_FOV0_CY5.png]

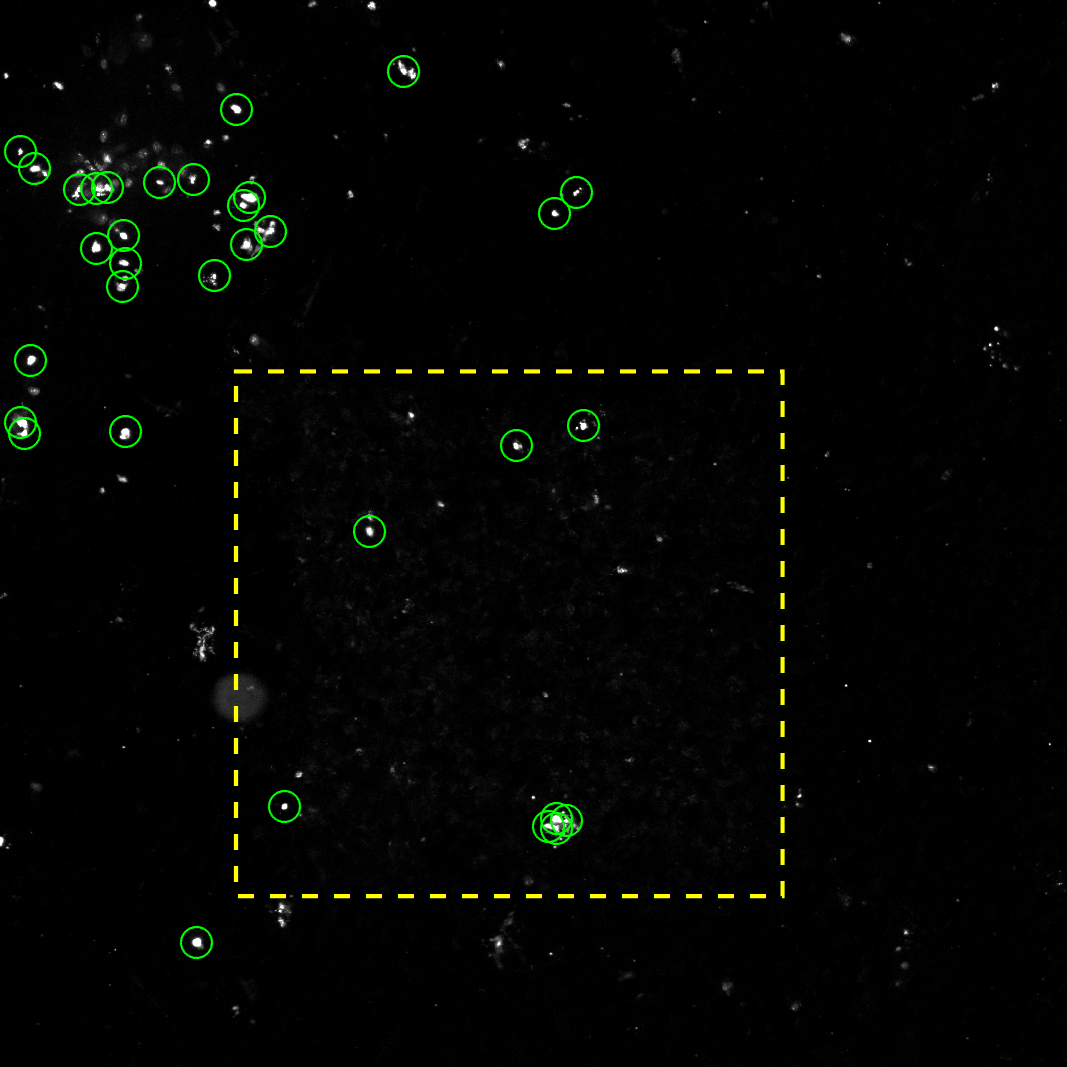

Supplement: Supplementary Software — Matlab code used for image analysis as well as LabVIEW code for microscope control [file ncomms11636-s3.zip › code/Viability/results/TIME48H_FOV0_PI.png]

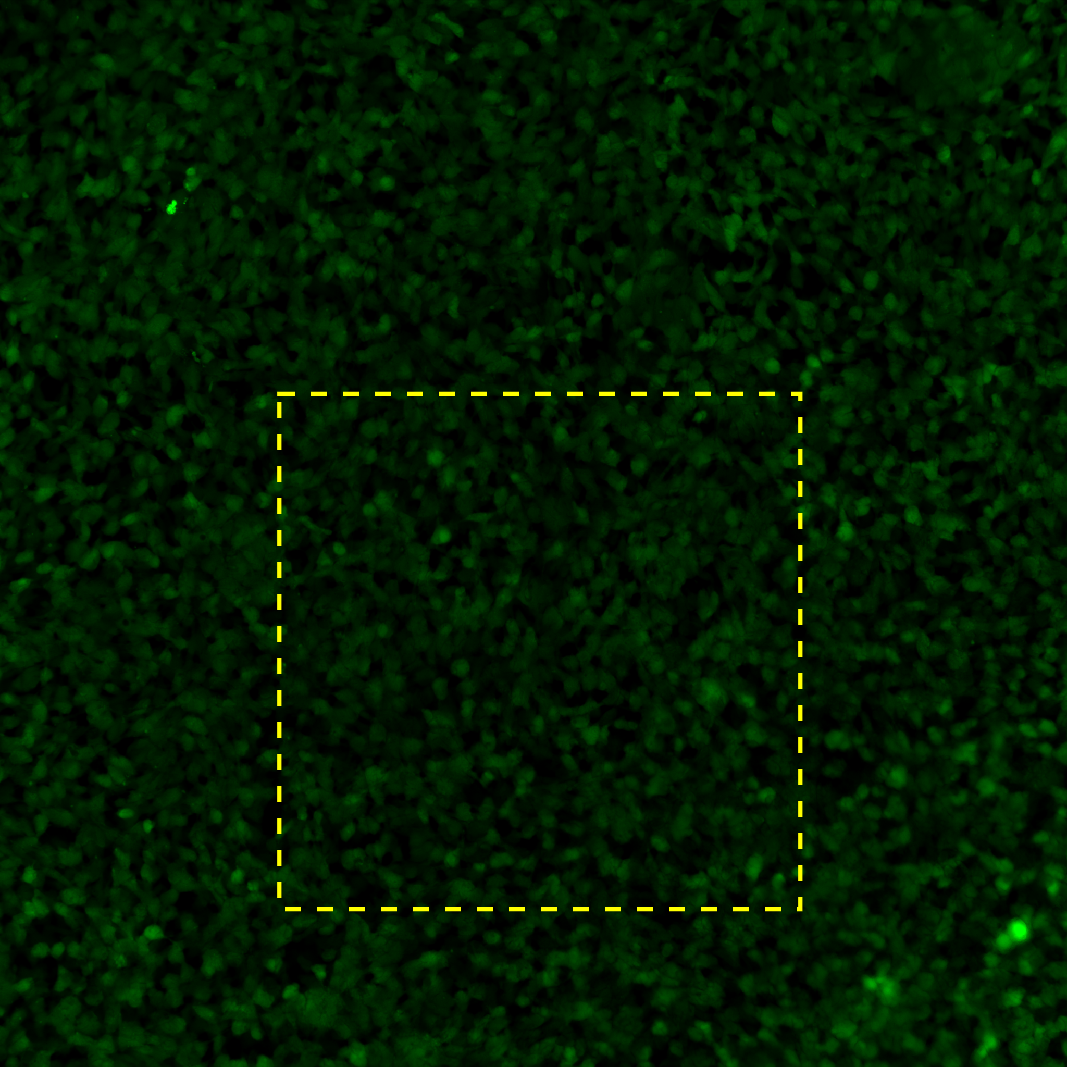

Supplement: Supplementary Software — Matlab code used for image analysis as well as LabVIEW code for microscope control [file ncomms11636-s3.zip › code/Viability/results/TIME48H_FOV1_CAL.png]

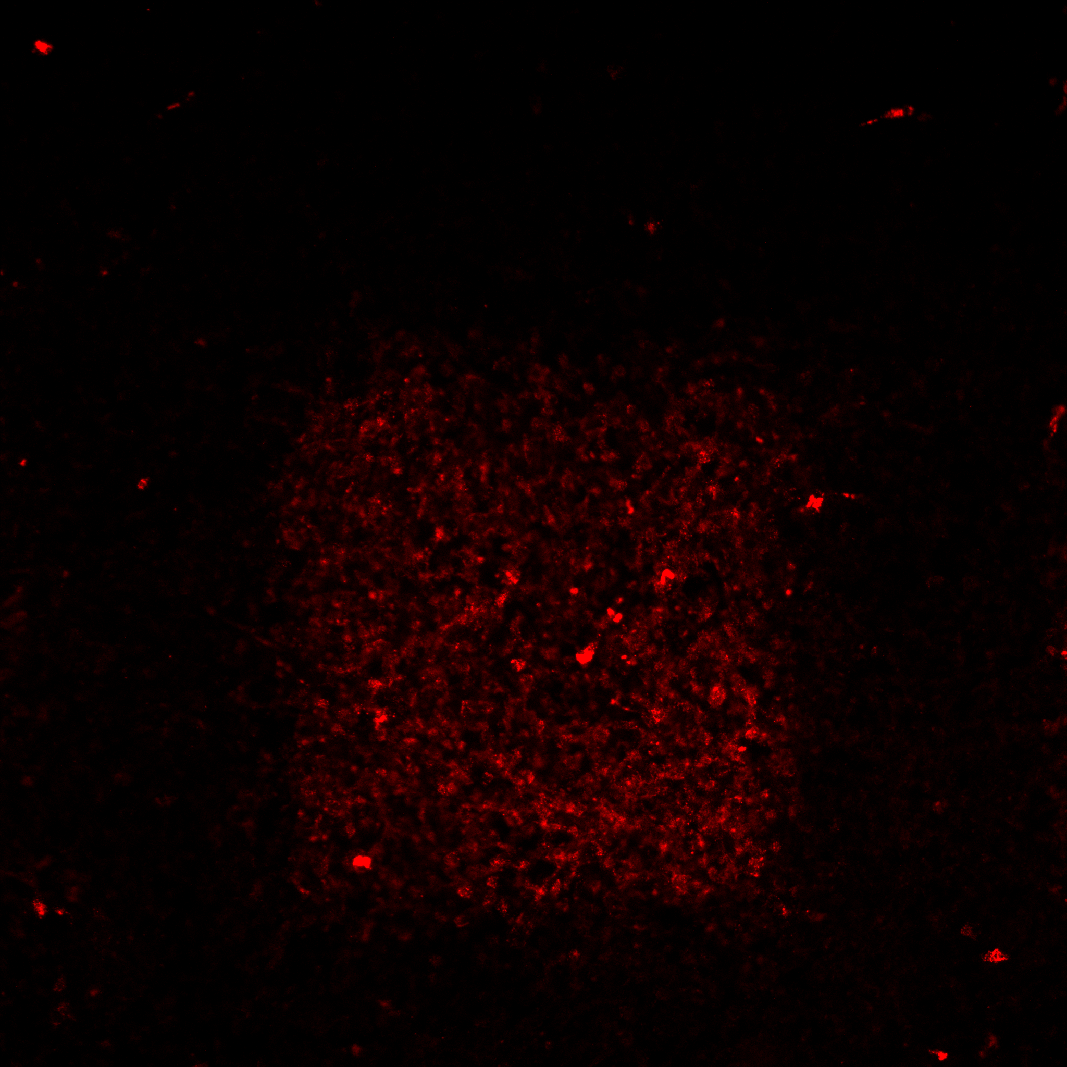

Supplement: Supplementary Software — Matlab code used for image analysis as well as LabVIEW code for microscope control [file ncomms11636-s3.zip › code/Viability/results/TIME48H_FOV1_CY5.png]

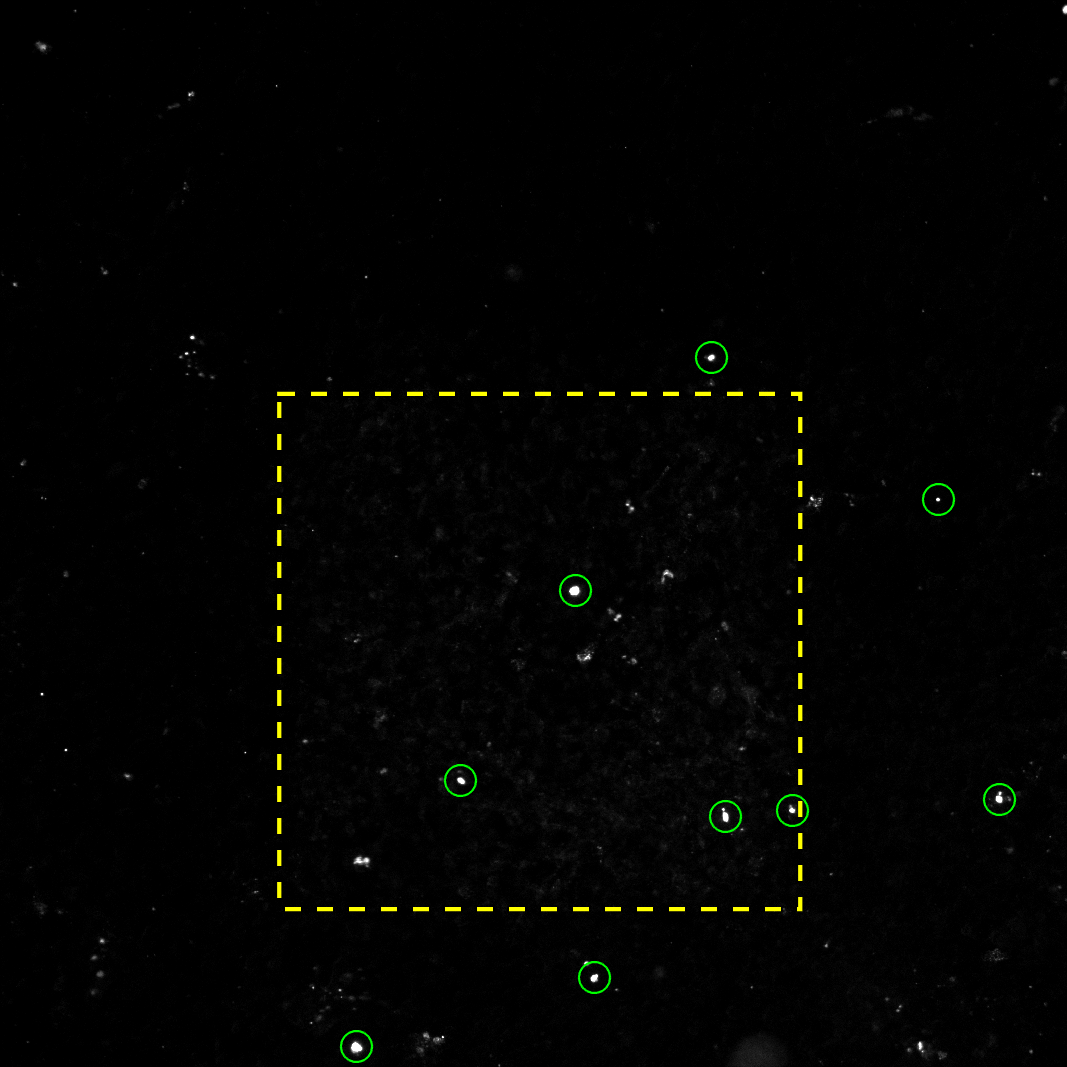

Supplement: Supplementary Software — Matlab code used for image analysis as well as LabVIEW code for microscope control [file ncomms11636-s3.zip › code/Viability/results/TIME48H_FOV1_PI.png]
